# Enhancing shear strength predictions of UHPC beams through hybrid machine learning approaches

**DOI:** 10.1038/s41598-025-13444-y

**Published:** 2025-08-02

**Authors:** Sanjog Chhetri Sapkota, Ajad Shrestha, Moinul Haq, Satish Paudel, Waiching Tang, Hesam Kamyab, Daniele Rocchio

**Affiliations:** 1 Department of Civil Engineering, Sharda University, Greater Noida, 201310 India; 2 Centre for Infrastructure Engineering and Safety, School of Civil and Environmental Engineering, The University of New South Wales, Kensington, Sydney, NSW 2052 Australia; 3 Interdisciplinary Research Center for Construction and Building Materials, Research Institute, King Fahd University of Petroleum & Minerals, Dhahran, 31261 Saudi Arabia; 4 Department of Civil and Environmental Engineering, University of Nevada, Reno, 1664 N Virginia St., Reno, NV 89557 USA; 5 School of Architecture and Built Environment, University of Newcastle, Callaghan, NSW 2308 Australia; 6 Department of Biomaterials, Saveetha Dental College and Hospital, Saveetha Institute of Medical and Technical Sciences, Chennai, 600077 India; 7 The KU-KIST Graduate School of Energy and Environment, Korea University, 145 Anam-ro, Seongbuk-gu, Seoul, 02841 Republic of Korea; 8 Universidad UTE, Quito, 170527 Ecuador; 9 LL Liminal Lab Investigation Group, Architecture Department, Faculty of Architecture and Urbanism, UTE University, Quito, 170527 Ecuador

**Keywords:** Ultra high-performance concrete (UHPC), Extreme gradient boosting (XGB), Giant armadillo optimization (GOA), Spotted hyena optimization (SHO), Leopard seal optimization (LSA), Shapley additive explanations (SHAP), Engineering, Materials science

## Abstract

Ultra-high-performance concrete (UHPC) beam shear strength prediction is a complicated process due to the involvement of numerous parameters. The accuracy needed for precise predictions is frequently lacking in current empirical equations and traditional machine learning (ML) techniques. This study proposes hybrid ML models that integrate three nature inspired metaheuristic algorithms—Giant Armadillo Optimization (GOA), Spotted Hyena Optimization (SHO) and Leopard seal optimization (LSA)- Extreme Gradient Boosting (XGB) to predict the shear strength of UHPC beams. A comprehensive dataset was created from extensive literature reviews and trained and tested on the models using multiple input parameters that affect UHPC’s shear capacity. For model assessment, performance metrics, such as coefficient of determination (R^2^), root mean square error (RMSE), mean absolute error (MAE), and variance accounted for (VAF), were utilized. Results showcased high accuracy, with R^2^ values approaching 0.9912 in training and 0.9802 in testing phases using the LSA-XGB algorithm, indicating excellent model fit and predictive reliability. To improve the model’s transparency and interpretability, the study also incorporates shapely additive explanations (SHAP), which reveal how each dataset attribute affects the predictive results. The LSA-XGB algorithm performed better than prior studies and empirical equations in predicting the shear strength of UHPC beams. More sophisticated machine learning techniques that improve the precision of predicting the shear capacity of UHPC beams are demonstrated in the study. Further, the use of a graphical user interface (GUI) helps researchers and engineers to make quick, well-informed decisions in real-time. These findings offer a reliable, interpretable, and accessible approach to predicting shear strength in UHPC beams, contributing to safer structural engineering practices.

## Introduction

Although conventional concrete has been popular in the civil engineering industry, the increasing development activities have led to a higher demand for improved performance of construction materials^1^. Due to the superior mechanical properties of ultra-high-performance concrete (UHPC), it has received significant attention in the civil engineering industry^2^. It has a superior compressive strength extending from 120 MPa to 150 at 28 days due to the dense packing of solid particles, improved hydration, and pozzolanic reactions^3–6^. This high strength reduces the structural member sizes constructed utilizing UHPC, resulting in better material utilization^7,8^. Compared to conventional concrete structural members, UHPC exhibits higher ductility, durability, and resistance to external environmental factors. UHPC has been used to construct bridges, high-rise buildings, and underground structures^9,10^. This application has further revealed the need to carry out more research on UHPC structural elements. Previous research on UHPC has worked on developing design philosophies, construction methods, and the capacity of UHPC members^11–13^. However, the ability to accurately predict the shear capacity of UHPC members remains challenging due to the material’s complexity and the numerous parameters involved^14–17^.

Due to the brittle nature of shear failure, it has always interested various scholars and researchers^18,19^. Structural members like deep beams and beam-column joints are commonly used in civil engineering applications and are dominated by shear failure^20–22^. Various experimental, numerical, and theoretical studies have been carried out to understand the shear behaviour of UHPC beams^23,24^. All these studies have given insights into shear behaviour. However, the prediction still needs to be precise since a limited number of parameters have been studied in these studies. These findings were mostly partial and based on specific experiments, making it difficult to generalize the shear behavior.

There is a lack of understanding of the shear mechanism of UHPC members, which has made it challenging to develop a suitable prediction model. Parameters like longitudinal reinforcement ratio, transverse reinforcement ratio, fiber content, shear span ratio, compressive strength, and cross-sectional area have significant impacts on the shear behaviour of UHPC beams^25^. Although various empirical models exist to predict the shear capacity of UHPC beams, they have been developed with a focus on the specific experimental results^14^. Due to this, the existing empirical model cannot predict the shear capacity of UHPC beams. Various theoretical models like the plasticity model, strut-and-tie model, truss-arch model, mesoscale fiber-matrix discrete model, and modified compression field theory also exist^26–29^. These models are based on some assumptions and utilize a limited number of parameters for prediction. The coefficients of these models are taken from regression analysis of a limited database, which results in an unsatisfactory prediction of the shear capacity of UHPC beams^15,30–32^. Also, the empirical and theoretical models provide high safety factors for the shear capacity of UHPC beams, which makes the design uneconomical and results in material utilization inefficiency^24^. With an increase in UHPC structural members in the real world, it is significantly necessary to precisely predict the shear capacity of UHPC structural members, which requires the exploration of new and advanced methods.

Rapid development of artificial intelligence (AI) provides an alternative solution to developing a prediction model. Machine learning (ML), a subset of AI, involves training algorithms to learn from and carry out data-based predictions^33^. Initially, traditional algorithms, including decision trees (DT), support vector machines (SVM), k-nearest neighbours (kNN), and artificial neural networks (ANN), were employed to predict the behaviour of materials and structures^34–40^. Similarly, more advanced machine learning models such as random forest (RF), gradient boosting machine (GBM), light gradient boosting machine (LightGBM), and extreme gradient boosting (XGB) have been employed, which have a better performance compared to the traditional machine learning models^38,41–44^. Compared to the empirical and theoretical models of shear beams, the ML model can predict the strength and performance of structural members^14^^,^^45^. Despite this precise prediction, it is hard to employ and understand this model due to the uncertainty of the interrelation between input and output features. Shapely additive explanations (SHAP) have been broadly used to interpret ML models. It has been utilized to predict the shear strength of slabs, interface, strength of concrete, and squat NC walls^43,44,46^.

Developing a reliable ML model provides good predictions and can be utilized to optimize the designs. Although ML models were developed long ago, very few works have been done to predict the shear capacity of UHPC beams using them. Solhmirzaei et al.^47^ utilized traditional machine learning algorithms such as SVM, ANN, and KNN to determine the failure modes of UHPC beams. Additionally, an equation was proposed to predict the shear strength of UHPC beams. Ni et al.^48^ utilized traditional machine learning and ensemble learning algorithms to predict the shear strength of UHPC beams and achieved a coefficient of regression (R^2^) of 0.90. Ye et al.^14^ utilized ensemble machine models to determine shear capacity. CatBoost, a part of the ensemble learning algorithm, performed better than other models. This research also utilized SHAP to understand the effect of input parameters on output parameters. The CatBoost model achieved an R^2^ of 0.94, which is better than previous research results. The literature indicates that utilizing ML models can precisely predict the shear strength of UHPC beams. Further, it was observed that the use of ensemble models has high prediction accuracy in comparison to traditional ML models. Such computing models substantially enhance the prediction accuracy and reliability of the shear strength of UHPC beams.

Previous research has primarily relied on traditional machine learning algorithms^49–51^, but this study introduces metaheuristic algorithms to enhance prediction accuracy in UHPC shear strength. This research employs machine learning methods to predict the shear capacity of UHPC beams and compares the results with existing codes. The gradient-based optimization of a comprehensive loss function makes XGB a powerful and accurate algorithm, overcome the weaknesses of sequential decision trees. More optimization techniques, including the Giant Armadillo Algorithm (GOA)^52^, Spotted Hyena Optimization (SHO)^53^, and Leopard Seal Optimization (LSA)^54^, that has been utilized in this study, enhance the model parameters in the course of its optimization process based on natural instincts. Effective data-driven modelling hinges on selecting the most suitable ML models, which directly affects how well the models fit the dataset and generate meaningful insights. While ML techniques are widely employed, there remains a critical gap in the utilization of innovative models capable of predicting the shear strength of UHPC beams.

Hyperparameter optimization is an essential yet challenging aspect due to the sheer number of possible combinations. Traditional techniques such as grid search, random search, and Bayesian optimization, though generally used, can be inefficient, computationally expensive and time-consuming. A more effective option is provided by a new highly efficient nature-inspired algorithm (NIA), which streamlines the search for ideal parameters, simplifies the procedure, and improves model performance. Furthermore, the model utilized in this research enables explainability of its behaviour, a capability not achievable with traditional models. Furthermore, practical challenges, such as the requirement for additional software and the lack of an intuitive graphical user interface (GUI), hinder the widespread adoption of advanced tools, such as models for estimating the shear strength of UHPC beams. By converting the GUI into an easily executable standalone file, researchers and engineers would be able to directly access the model’s estimation capabilities without the need for other tools. This strategy would be effective in making a quick and well-informed decision for predicting the shear strength of UHPC beams.

Shear behaviour is a complex phenomenon due to the involvement of numerous influential parameters, including beam geometry, material properties, reinforcement ratios and fiber aspect ratio. Current design codes are inefficient in predicting the shear strength due to these complexities. This limitation emphasizes the need for ML models to achieve optimized designs and enhance structural reliability. This current study compares the performance of the adopted model with the existing design codes, highlighting its potential for practical real-world applications.

The novelty of this study lies in utilizing hybrid ML models by combining NIA with XGB. This method of hyperparameter tuning has never been used before for UHPC shear prediction. Also, the model transparency measurement is achieved via SHAP, providing global and local interpretability on how the individual input parameter affects the results, narrowing the gap between predictive accuracy and explanations. Lastly graphical user interface (GUI) was developed to facilitate the ease of carrying out real time prediction of shear strength to be used by engineers and researchers to make swift yet informed structural design judgments. These contributions enhance the overall understanding of UHPC shear strength models by providing more accurate, intuitive, and accessible representations.

## Database description

Building effective ML models relies heavily on gathering a comprehensive and trustworthy dataset. For creating highly accurate predictive models, it is crucial to use a large-scale dataset where the sample size substantially surpasses the number of independent parameters. Research suggests that having a sample size 10 to 20 times greater than the number of independent parameters can be adequate for developing reliable and well-generalized ML models. This study uses a dataset of 549 records of UHPC beam shear capacities from extensive literature reviews to align with this goal^14,25,55^ (*refer to* Supplementary Material-1). The dataset consists of twenty input parameters such as geometric dimensions, longitudinal reinforcement ratio, yield strength of longitudinal reinforcement and stirrups, ratio of prestressing tendons, normal stress at the neutral axis, stirrups ratio, spacing of stirrup, material properties of UHPC and shear span to depth ratio as detailed in Table 1.

**Table 1:** Experimental database and features adopted in the current study.

Variables	Symbols	Category	Min.	Max.	Mean	Median	S.D.
Height	h	Input	76	1092.00	363.38	330.00	194.74
Breadth	b	Input	20	250.00	105.98	100.00	53.29
Top flange width	bf1	Input	0	737.00	146.21	0.00	184.65
Top flange thickness	tf1	Input	0	190.00	33.15	0.00	39.06
Bottom flange width	bf2	Input	0	737.00	97.02	0.00	150.53
Bottom flange thickness	tf2	Input	0	228.00	43.12	0.00	60.64
Cross-sectional area	Ac	Input	8400	276,939.38	54,286.28	43,400.00	43,082.82
Longitudinal reinforcement ratio	pl	Input	0	0.22	0.05	0.04	0.05
Yield strength of longitudinal reinforcement	fsy	Input	0	1100.00	444.33	491.20	247.65
Ratio of prestressing tendons	pp	Input	0	0.07	0.01	0.00	0.02
Normal stress at the neutral axis	sp	Input	0	27.07	2.97	0.00	6.14
Stirrup ratio	s	Input	0	500.00	57.34	0.00	97.82
Yield strength of stirrups	psv	Input	0	0.02	0.00	0.00	0.00
Spacing of stirrups	fsv	Input	0	731.00	162.93	0.00	231.50
Compressive strength	fc	Input	78	224.00	143.39	138.10	26.47
Fiber content	pf	Input	0	5.00	1.50	1.60	0.81
Fiber length	lf	Input	0	60.00	14.45	13.00	9.83
Fiber diameter	df	Input	0	1.00	0.22	0.20	0.16
Reinforcing index	λf	Input	0	4.74	1.01	1.18	0.56
Shear span-to-depth ratio	m	Input	0.554	8.00	2.61	2.50	1.04
Shear strength	Vu	Output	18.2	3052.98	430.64	356.00	402.34

**Fig. 1:**
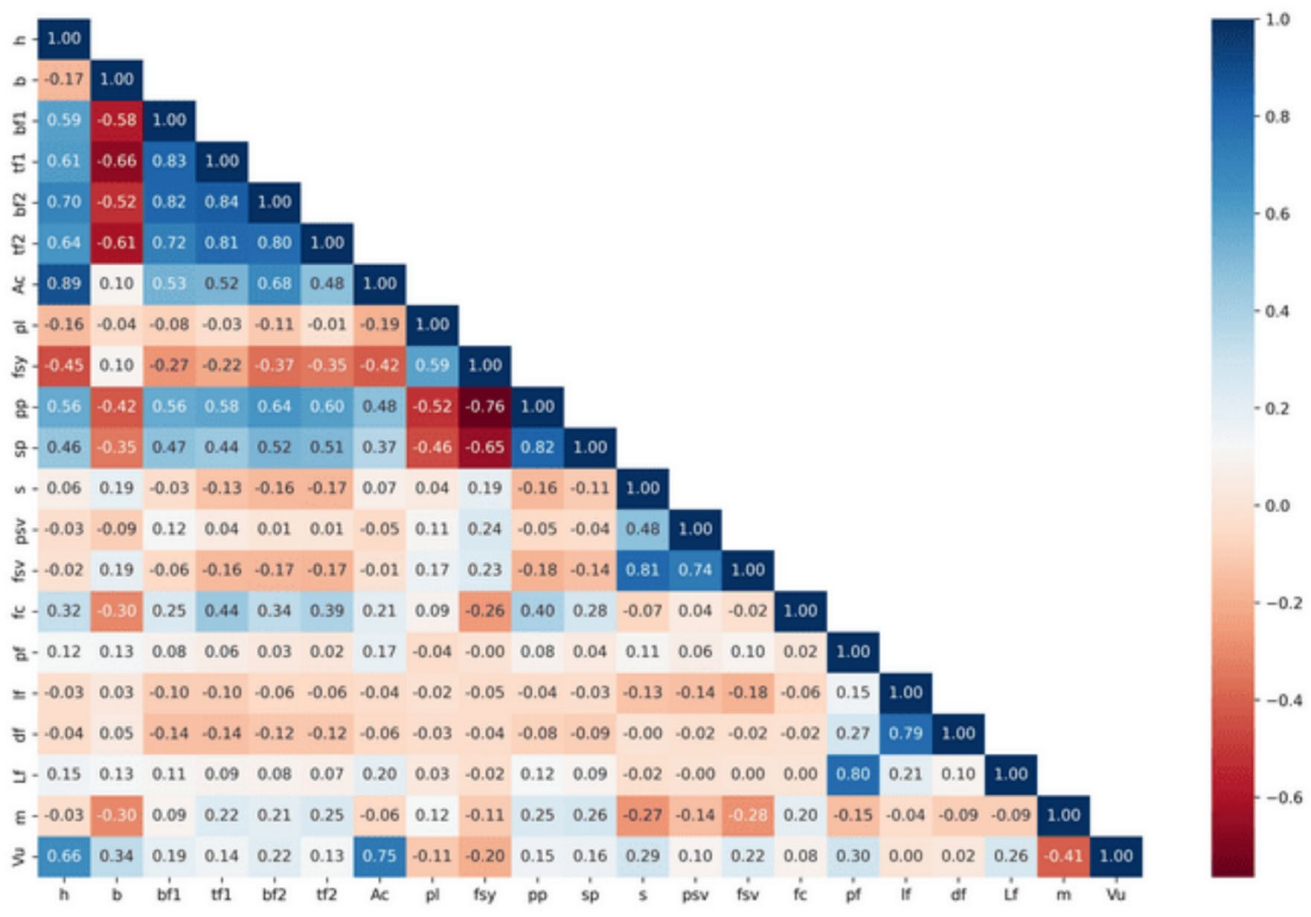
Correlation between input and output parameters.

A heatmap of the Pearson correlation coefficient is presented in Fig. 1, which shows a linear correlation among pairs of variables in the dataset. The coefficients are between − 1.0 and 1.0, − 1.0 being a strong negative correlation, 1.0 being a strong positive correlation, and 0 being little or no linear correlation. Strong positive correlations of up to 0.84 and strong negative correlations down to -0.66 are notable. The underlying meaning of these values is that some pairs of features are highly correlated, and this can affect the model prediction, as well as the outcome interpretation.

**Fig. 2:**
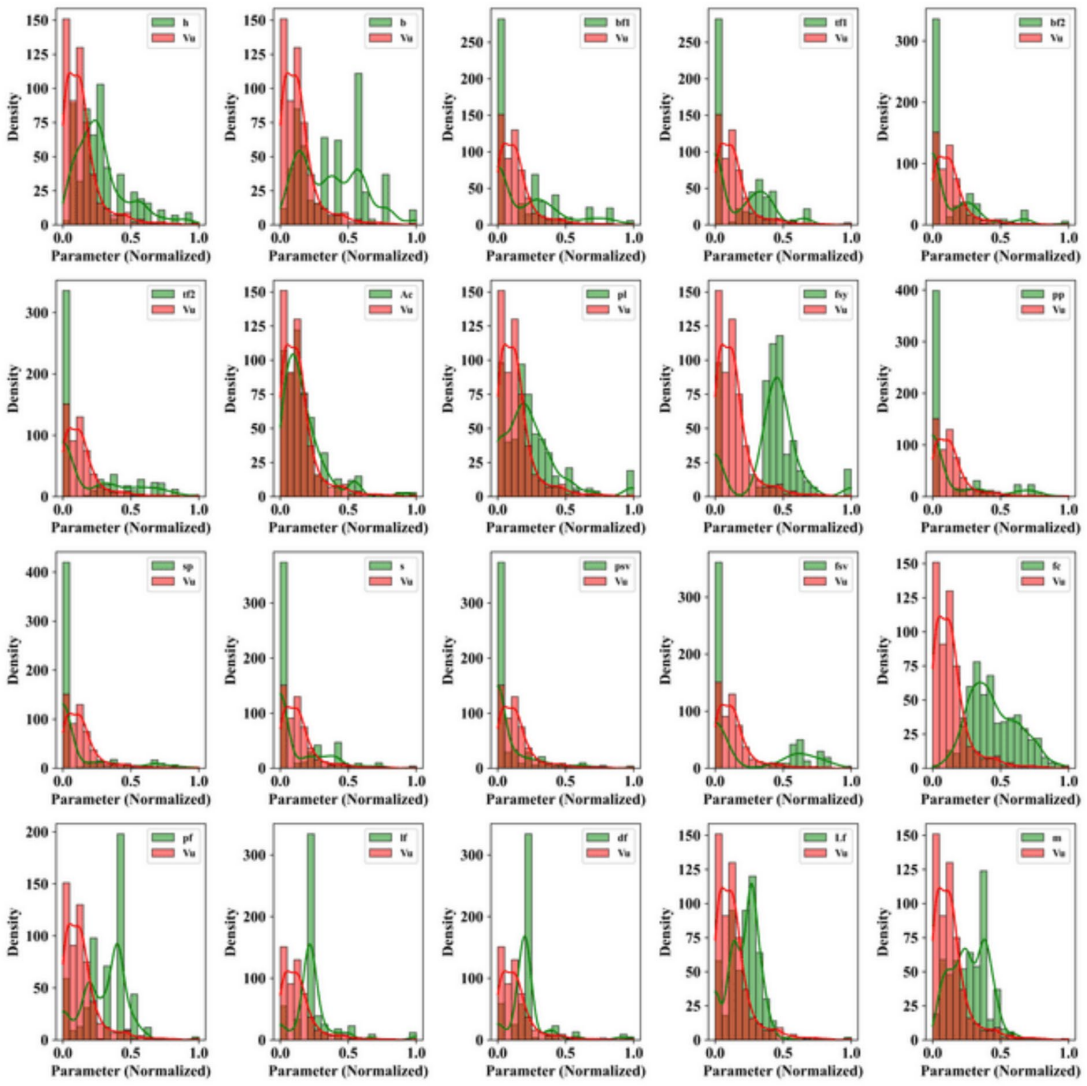
Relationship between each feature.

Figure 2 represents the histogram somewhat associated with the Kernel Density Estimator density (KDE) of the two sets of datasets, Vc and Vs, with the comparison of densities of numerous normalized traits. The frequency distribution of values in histograms is visualized in terms of bars, but the KDE plots smooth lines and curves that depict each probability density function of a given dataset. The normalized values are taken on the x-axis, and the constant density of the frequency of these values is taken on the y-axis; each subplot denotes one feature. The dataset showing ‘Vc’ is shown in red curves, and the dataset showing ‘Vs’ is shown in green curves. It has been found that the concentration of the Vc data lies heavy at the low side of the scale of normalized values, and those of Vs data, on the other hand, fall more or less uniformly throughout the scale. When distribution of an input resembles that of Vu substantially, then the output may indicate greater statistical dependence or sensitivity, which may indicate a greater contribution to the change in output. Low correlation or impact, on the other hand, is indicated by large differences between the shape, spread or peak position of an input and Vu. Features such as bf1, pl, fv, and vr have shape distributions quite different to Vu, suggesting lower statistical dependence and possible limited significance to the output. Other inputs, like Ac, fy, and pp, are moderately similar, implying that there is a certain or conditional effect. The figure shows the sharpness and skew of distributions of inputs such as vp, df and lf, which will be found to have low influence or may interact with Vu in more complex ways. Such a visualization is beneficial to differentiate and characterize peculiarities of behaviors and properties of each dataset and provide a numerical basis for further analysis of their characteristics.

**Fig. 3:**
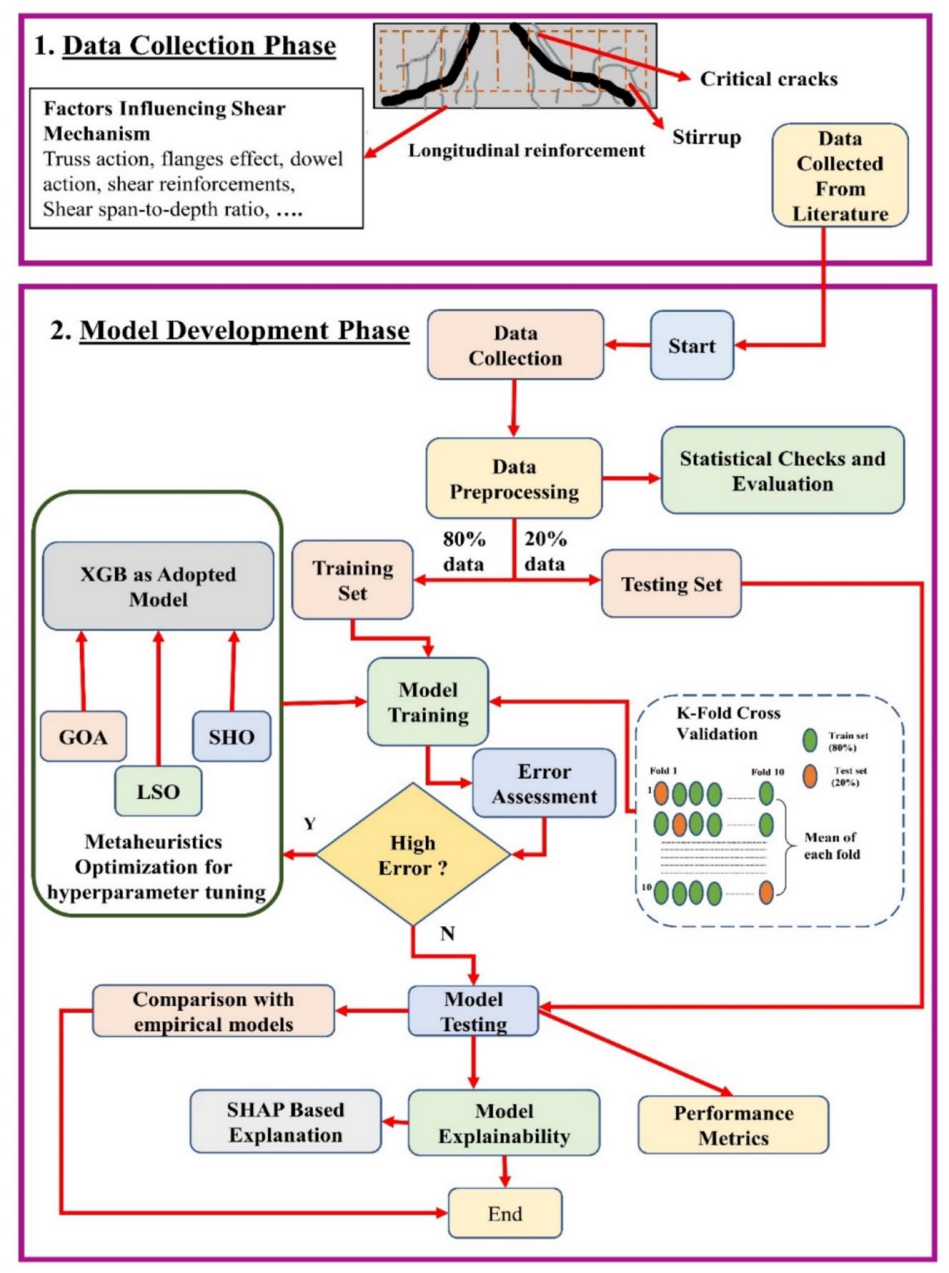
Research methodology for the current study.

Figure 3 illustrates a systematic research methodology used for this project. Initially, the data that influenced the shear behavior was collected from various studies. Later, for developing the model, these data were used to develop, train, and test a model. First, the data was cleaned, and then it was divided into a training dataset, which had 80% of the data, and a testing dataset, which had 20% of the data. The model used XGB as the main predictive method and methods of mathematical approaches, including GOA, LSA, and SHO set hyperparameters. A K-Fold Cross-Validation increases model reliability as it allocates each set of data to be used in training and testing the model. When high errors were detected, further optimization was done. Subsequently, the accuracy of the model was compared with empirical models to verify the model’s performance after meeting the satisfactory level. Finally, SHAP was applied to explain the model and to determine the importance of each parameter in the shear behavior predictions. This systematic approach resulted in an efficient and easily interpretable model that could predict the shear strength of UHPC beams.

## Soft computing techniques

### Extreme gradient boosting (XGB)

XGB is a widely adopted ensemble model based on the framework of gradient boosting. It is used to predict output with the help of several decision trees^56–58^. Such trees are analyzed as weak learning trees, and the errors produced are rectified in the upcoming trees, making them strong learning trees. This process involves reducing the loss function, predicting the weak learners, and adding the extra loss function to minimize the error. The use of learning rate, depth of the tree, and regularization techniques helps in optimizing the performance very well. The loss function is calculated using Eqs. (1) and (2).

1 $${\mathcal{L}}^{t}=\sum_{i=1}^{n}l\left({y}_{i,}{\widehat{y}}_{i}^{\left(t-1\right)}+{f}_{t}\left({x}_{i,}\right)\right)+\Omega \left({f}_{t}\right)$$

2 $$\Omega \left(f\right)=\gamma T+\frac{1}{2}\lambda {\Vert \omega \Vert }^{2}$$

where ${\mathcal{L}}^{t}$ = loss function, ${y}_{i,}\widehat{y} =$ marked data and forecasted data, ${f}_{t}$ = model of t^th^ tree, t = iteration index, T = total number of tree leaves, $\lambda$ and $\gamma$ are penalty coefficients, $\omega$ is the vector having a score for each leaf.

### Giant Armadillo algorithm (GOA)

The Giant Armadillo Algorithm (GOA) is a recently developed metaheuristics algorithm inspired by the hunting behaviour to target termite mounds by Giant Armadillos^52^. The metaheuristics algorithm is mathematically modelled using two stages. First, the exploration of space focuses on the movement of giant armadillos in search of termite mounds. The final step is based on the exploitation of spaces. It focuses on the digging strategies of giant armadillos to attack the mounds of termites. The decision variable values are initialized using Eq. (3) according to the starting positions of Giant Armadillos in the matrix space representation provided in Eq. (4). Although effective, MLPA lacks accuracy when the prey is in proximity to one seal (leader) while being far from other seals. To address this deficiency, Weighted Leaders Prey Allocation (WLPA) is used, whereby designated leaders are allocated a weight contingent upon their proximity to prey, denoted as w_i. The anticipated location of the prey might be denoted as

3 $${x}_{i,d}=l{b}_{d}+r \cdot \left(u{b}_{d}-l{b}_{d}\right)$$

4 $$X = \left[ {\begin{array}{*{20}c} {X_{1} } \\ \vdots \\ {\begin{array}{*{20}c} {X_{i} } \\ {\begin{array}{*{20}c} { \vdots } \\ {X_{N} } \\ \end{array} } \\ \end{array} } \\ \end{array} } \right]_{N \times m} = \left[ {\begin{array}{*{20}c} {x_{1,1} } & \ldots & {x_{1,d} } & \ldots & {x_{1,m} } \\ \vdots & { \ddots } & & {\mathinner{\mkern2mu\raise1pt\hbox{.}\mkern2mu \raise4pt\hbox{.}\mkern2mu\raise7pt\hbox{.}\mkern1mu}} & \vdots \\ {x_{i,1} } & \ldots & {x_{i,d} } & \ldots & {x_{i,m} } \\ \vdots & {\mathinner{\mkern2mu\raise1pt\hbox{.}\mkern2mu \raise4pt\hbox{.}\mkern2mu\raise7pt\hbox{.}\mkern1mu}} & \vdots & \ddots & { \vdots } \\ {x_{N,1} } & \ldots & {x_{N,d} } & \ldots & {x_{N \times m} } \\ \end{array} } \right]_{N \times m}$$

where X denotes the population matrix of GOA, X_i_ is the i^th^ position state of GOA member. x_i,d_ is the d^th^ search space dimension, N is the total number of giant armadillos, m is the number of variables, r denotes random number space in the interval [0,1], lb_d_ and ub_d_ indicate the lower and upper bound of the d^th^ variable, respectively. Equation (5) evaluates the objective function value for each candidate solution presented by the position of Giant Armadillo.

5 $$F={\left[\begin{array}{c}{F}_{1}\\ \vdots \\ \begin{array}{c}{F}_{i}\\ \vdots \\ {F}_{N}\end{array}\end{array}\right]}_{N\times 1}={\left[\begin{array}{c}F\left({X}_{1}\right)\\ \vdots \\ \begin{array}{c}F\left({X}_{i}\right)\\ \vdots \\ F\left({X}_{N}\right)\end{array}\end{array}\right]}_{N\times 1}$$

Here, F and F_i_ are the vector of the objective function and the objective function for the i^th^ GOA member, respectively.

The set of such locations for each member is shown in Eq. (6). The new position is randomly updated to one of the condition termite mounds from the set of candidate termite mounds and attacked using Eq. (7).

6 $$T{M}_{i}=\left\{{X}_{k}:{F}_{k}<F{}_{i} and k\ne i\right\}, where i=\text{1,2}, \dots , N and k\in \left\{\text{1,2}, \dots , N\right\}$$

Here, TM_i_ is the location for the i^th^ giant armadillo for the set of candidate termite mounds’, X_k_ is the total population member with optimal objective function value than the i^th^ giant armadillo as shown in Eq. (8)

7 $${x}_{i,j}^{P1}={x}_{i,j}+r{}_{i,j} \cdot \left(STM{}_{i,j}-{I}_{i,j} \cdot {x}_{i,j}\right)$$

8 $$X_{i} = \left\{ {\begin{array}{*{20}c} {X_{i}^{{P1}} ,} & {F_{i}^{{P1}} < F_{i} ,} \\ {X_{i} ,} & {else} \\ \end{array} } \right.$$

Here, STM_i_ is the adopted termite mound for the i^th^ giant armadillo, STM_i,j_ is its j^th^ dimension, ${X}_{i}^{P1}$ is the updated position calculated for the i^th^ giant armadillo. The attacking phase of the proposed GOA, ${x}_{i,j}^{P1}$ is its j^th^ dimension, ${F}_{i}^{P1}$ is its objective function value, $r{}_{i,j}$ are arbitrary numbers from the interval [0, 1], and I_i,j_ are numbers that are arbitrarily selected.

In the exploitation phase, the digging action of a giant armadillo to open the mound and prey on the termites is modelled. This results in minor changes in the giant armadillo’s position, which increases the algorithm’s exploitation in the local search space. Thus, a new position calculated based on Eq. (9) is changed by the initial position of the member according to Eq. (10), where the value of the objective function is enhanced.

9 $${x}_{i,j}^{P2}={x}_{i,j}+\left(1-2{r}_{i,j}\right) \cdot \frac{u{b}_{j}-l{b}_{j}}{t}$$

10 $$X{}_{i}=\left\{\begin{array}{c}\begin{array}{cc}{X}_{i}^{P2}, & {F}_{i}^{P2}\le F{}_{i},\end{array}\\ \begin{array}{cc}{X}_{i},& else\end{array}\end{array}\right.$$

Here, ${X}_{i}^{P2}$ is the updated position calculated for the i^th^ giant armadillo in the digging phase of the proposed GOA, ${x}_{i,j}^{P2}$ is the j^th^ dimension, ${F}_{i}^{P2}$ is its optimized objective function value, ${r}_{i,j}$ are the arbitrary numbers from the [0, 1] interval, and t is the total iteration.

### Spotted Hyena Optimization (SHO)

Spotted Hyena Optimization (SHO) is a nature-inspired metaheuristic algorithm drawn from the lifestyle followed by a spotted hyena^53^. The unique behaviour of hyenas is represented by the female hyena being the most dominant member. Further, the hyenas combine sounds similar to human laughter to investigate the food source. The mathematical modelling of the SHO algorithm involves encircling the prey, hunting the prey, attacking, and searching. The SHO algorithm is efficient in finding the best solution over further iteration. The involved equation as shown in Eqs. (11) and (12) represents the different steps followed:

11 $${\overrightarrow{D}}_{h}=\left|\overrightarrow{B}\cdot {\overrightarrow{P}}_{p}\left(x\right)-\overrightarrow{P}\left(x\right)\right|$$

12 $$\overrightarrow{P}\left(x+1\right)={\overrightarrow{P}}_{p}\left(x\right)-\overrightarrow{E}\cdot {\overrightarrow{D}}_{h}$$

where ${\overrightarrow{D}}_{h}$ define the distance between the prey and the hyena (see Eq. 13), $x$ indicates the updated iteration, $\overrightarrow{B}$ and $\overrightarrow{E}$ are co-efficient vectors, ${\overrightarrow{P}}_{p}$ indicates the position vector of the prey, $\overrightarrow{P}$ is the position vector of the spotted hyena.

$$\overrightarrow{B}=2\cdot r{\overrightarrow{d}}_{1}$$

$$\overrightarrow{E}=2\overrightarrow{h}\cdot r{\overrightarrow{d}}_{2}-\overrightarrow{h}$$

13 $$\overrightarrow{h}=5-\left(Itr\right.*\left(5/\right.Max\left.\left.{}_{\text{Itr }}\right)\right),\hspace{0.17em}Itr=1,2,3,\dots ,Ma{x}_{\text{Itr}}$$

The hunting behaviour can be modeled using Eq. (14).

$${\overrightarrow{D}}_{h}=\left|\overrightarrow{B}\cdot {\overrightarrow{P}}_{h}-{\overrightarrow{P}}_{k}\right|$$

$${\overrightarrow{P}}_{k}={\overrightarrow{P}}_{h}-\overrightarrow{E}\cdot {\overrightarrow{D}}_{h}$$

14 $${\overrightarrow{C}}_{h}={\overrightarrow{P}}_{k}+{\overrightarrow{P}}_{k+1}+\dots +{\overrightarrow{P}}_{k+N}$$

where ${\overrightarrow{P}}_{h}$ represents the position of the first best-spotted hyena, ${\overrightarrow{P}}_{k}$ represents the position of other spotted hyenas. $N$ is the number of spotted hyenas as calculated by Eq. (15).

15 $$N={\text{count}}_{\text{nos }}\left({\overrightarrow{P}}_{h},{\overrightarrow{P}}_{h+1},{\overrightarrow{P}}_{h+2},\dots ,\left({\overrightarrow{P}}_{h}+\overrightarrow{M}\right)\right)$$

where $\overrightarrow{M}$ is a random vector in $\left[0.5,1\right]$, nos defines the representation of solutions and counts all candidate solutions. Also, ${\overrightarrow{C}}_{h}$ represents a group or cluster of $N$ number of optimal solutions. The attack is done accordingly to Eq. (16).

16 $$\overrightarrow{P}\left(x+1\right)=\frac{{\overrightarrow{C}}_{h}}{N}$$

where $\overrightarrow{P}\left(x+1\right)$ save the best solution and update the search agents’ positions.

### Leopard Seal Optimization (LSA)

Leopard Seal Optimization (LSA) is a novel optimization method inspired by the foraging behaviour of leopard seals^54^. They always stay alert in the search for prey, regardless of their level of hunger. Their behaviour demonstrates that they practice self-distribution by assigning a certain search range to everyone. Upon the finding of prey by a member of the seal herd, the other members gather nearby and initiate a unified strike. The LSO model has three sequential phases: (I) prey search, (II) prey encirclement, and (III) prey attack. The following section numerically illustrates the predation dominance of lion seals.

The position vectors of leopard seals during their movement in each cycle, denoted by $\xi$, are maintained properly. The initial point of SA of the i^th^ cycle (${\overrightarrow{X}}_{init}^{j}$) is known beforehand as it indicates the ending point of the spiral in (i−1)^th^ iteration, which can also be denoted by ${\overrightarrow{X}}_{\xi }^{i-1}$. ${\overrightarrow{X}}_{1}^{i}$ and ${\overrightarrow{X}}_{ \xi }^{i}$ denote the initial and end points of the current and the i^th^ iteration, respectively. During each search phase iteration, $\xi$ position vectors of roaming leopard seals are denoted as ${\overrightarrow{X}}_{p}^{i} \forall p\in \{\text{1,2},3,\dots , \xi \}$ and the objective function’s value is ${F(\overrightarrow{X}}_{p}^{i}) \forall p\in \{\text{1,2},3,\dots , \xi \}$. The $\xi$ position vectors of the i^th^ iteration of seal ${L}_{m}$ is calculated by Eq. (17).

17 $${\overrightarrow{X}}_{\alpha }^{i}({L}_{m})|\forall \alpha \in \text{1,2},3,\dots , \xi -1={\overrightarrow{D}}^{i}({L}_{m}).{e}^{b{f}_{\alpha }}\text{cos}\left(2\pi {f}_{\alpha }\right)+ {\overrightarrow{X}}_{ \xi }^{i}{L}_{m}$$

where ${\overrightarrow{X}}_{\alpha }^{i}({L}_{m})$ is the new $\alpha$’s position of ${L}_{m}$ at the final i^th^ iteration and ${\overrightarrow{D}}^{i}({L}_{m})$ is the distance between the leopard seal and its prey.

The second phase is for the encirclement of the prey, in which the search agent curbs the prey and finds the optimal solution. The prime hindrance of this phase is that the position of the prey is not pre-known, hence the need to allocate the prey’s position beforehand. Single-leader prey Allocation (SLPA) is the simplest method for the allocation of prey. Based on SLPA, the prey’s position is demonstrated as shown in Eq. (18).

18 $${\overrightarrow{X}}_{prey}=Position \left[\begin{array}{c}argmax\_Validation [F\left({L}_{m}\right)] \\ \forall {L}_{m}\in S\end{array}\right]$$

where S are search agent sets and $F\left({L}_{m}\right)$ is a fitness function for the LSA algorithm. Another method is Multi Leader Prey Allocation (MLPA). If there are k leaders in z-dimensional space, the position vector of ${i}^{th}$ leader can be denoted as: ${\overrightarrow{X}}_{i}=[{x}_{1i}, {x}_{2i}, {x}_{3i}\dots .., {x}_{zi}]$. The prey’s predicted position can be represented by Eq. (19).

19 $${\overrightarrow{X}}_{prey}=\frac{1}{k}\left(\genfrac{}{}{0pt}{}{\begin{array}{c}{\sum }_{i=1}^{k}{x}_{1i}\\ {\sum }_{i=1}^{k}{x}_{2i}\\ \dots \end{array}}{\begin{array}{c}\dots \\ \dots \\ {\sum }_{i=1}^{k}{x}_{zi}\end{array}}\right)$$

If the prey is located extremely near to a leader seal and very distant from other seals, MLPA, although effective, is not accurate. To rectify this deficiency, Weighted Leaders Prey Allocation (WLPA) is used, whereby designated leaders are allocated a weight contingent upon their proximity to prey, denoted by an assigned weight w_i. The anticipated location of the prey might be denoted by Eq. (20).

20 $${\overrightarrow{X}}_{prey}=\frac{1}{{\sum }_{m=1}^{k}W({L}_{m})}\left(\genfrac{}{}{0pt}{}{\begin{array}{c}{\sum }_{m=1}^{k}W\left({L}_{m}\right).{x}_{1m}\\ {\sum }_{m=1}^{k}W\left({L}_{m}\right).{x}_{2m}\\ \dots \end{array}}{\begin{array}{c}\dots \\ \dots \\ {\sum }_{m=1}^{k}W\left({L}_{m}\right).{x}_{zm}\end{array}}\right)$$

When the prey is encircled and cannot move further, the agents come nearer the prey and eventually capture it. The distance between the agent and the prey (${\overrightarrow{D}}_{{L}_{m}}^{i})$ in this phase is calculated by Eq. (21).

21 $${\overrightarrow{D}}_{{L}_{m}}^{i}=\left|{\overrightarrow{X}}_{prey}^{i}-{\overrightarrow{X}}_{{L}_{m}}^{i}\right|$$

## Modelling and hyperparameter tuning

K-fold cross-validation is a reliable and extensively used technique to reduce overfitting problems. The procedure starts when the subdivided training data uses the remaining subgroup data for testing and the K−1 data of the whole K fold^59^. Every iteration of this algorithm uses the validation data once. The final model’s performance is defined as the mean performance across all k folds in testing and training. If an accepted model performs well during training but poorly on unknown data, it is deemed overfitted. For this study, the dataset was first divided into 80:20 training and evaluation sets. It had 80% of the training data and 20% of the assessment data, which were not repeated in the training set; tenfold cross-validation that used 80% of the total data for training may be a better option to avoid bias in the test set. There were ten sets in total: nine of them were employed for training the dataset, and the tenth set was employed for testing the dataset. The modelling was considered to be valid after the total performance in every fold was calculated. It turned out that each fold had different data in the testing set. However, to enhance the models’ performance before the training set, the hyperparameters needed to be tuned. In other words, hyperparameters are the variables that exist outside the model and are used by the algorithm to fine tune the performance of the model in question. This leads to the efficient tuning of hyperparameters and therefore an increase in the model’s accuracy. XGB adjusted parameters, including learning rate, max_depth, and n_estimators, to enhance the model’s efficiency and accuracy. The GOA, SHA, and LSA are metaheuristic algorithms that optimize the hyperparameters of the XGB algorithm. Defining the MHA parameters and establishing the bounds before optimization. For MHA, the parameters specified are population (n_pop), maximum iterations (max_iter), and number of hyperparameters (dim). Subsequently, to ascertain a standard hyperparameter value, optimization is performed to minimize the cost function, namely the Root Mean Square Error (RMSE). Both approaches possess the following parameters: dim = 3, cost_func = RMSE, max_iter = 100, and n_pop = 50. Table 2 presents the comprehensive hyperparameter results.

**Table 2:** Hyperparameter result of the applied hybrid model.

Model	Hyperparameters used	Optimized combination	Parameters of applied MHA
GOA-XGB	n_estimator	66.72	n_pop = 50, max_iter = 100, cost_func = RMSE, dim = 3
	max_sample split	3.977	
	max_depth	8.324	
SHO-XGB	n_estimator	85.536	
	max_sample split	2.672	
	max_depth	11.348	
LSA-XGB	n_estimator	147.804	
	max_sample split	0.6787	
	max_depth	3.837	

## Statistical performance indices

The assessment of the model’s performance in training and testing sets is crucial prior to the deployment of the model. This assessment is possible using datasets with performance indicators that still need to be made evident. By advancing the model’s generalization, each metric strengthens the model’s reliability. Equations (22–27) present the six statistical parameters that are employed in this inquiry. To improve the performance evaluation, several statistical indicators may be used, including R^2^, variance account factor (VAF), mean absolute error (MAE), Willmott’s index of agreement (WI), Root Mean Square Error (RMSE), and a20 index.

22 $${R}^{2}=\frac{\sum_{i=1}^{N}{\left({y}_{i}-{y}_{mean}\right)}^{2}-\sum_{i=1}^{N}{{(y}_{i}-{\widehat{y}}_{i})}^{2}}{\sum_{i=1}^{N}{\left({y}_{i}-{y}_{mean}\right)}^{2}}$$

23 $$\text{RMSE}=\sqrt{\frac{1}{N}\sum_{i=1}^{N}{{(y}_{i}-{\widehat{y}}_{i})}^{2}}$$

24 $$MAE=\frac{1}{N}\sum_{i=1}^{N}|{\widehat{y}}_{i}-{y}_{i}|$$

25 $$WI=1-\left[\frac{\sum_{i=1}^{N}{{(y}_{i}-{\widehat{y}}_{i})}^{2}}{{\sum_{i=1}^{N}\left\{|{\widehat{y}}_{n}-{y}_{mean}|+|{y}_{n}-{y}_{mean}|\right\}}^{2}}\right]$$

26 $$VAF\left( \% \right) = \left( {1 - \frac{{VAR\left( {y_{i} - \hat{y}_{i} } \right)}}{{VAR(y_{i} )}}} \right) \times 100$$

27 $$a20 index=\frac{n20}{n}$$

where, y_i_ = i^th^ measured value, ${\widehat{{\varvec{y}}}}_{{\varvec{i}}}$ = i^th^ predicted value, y_mean_ = mean of the measured value, N = total number of readings, R^2^ = coefficient of determination, RMSE = Root Mean Squared Error, MAE = Mean absolute error, WI = Willmott’s Index of Agreement, VAF = Variance Account Factor.

## Results and discussion

### Performance comparison of the employed model

The performance of the hybrid machine learning models—GOA-XGB, SHO-XGB, and LSA-XGB for predicting the shear strength of UHPC beams is presented in Tables 3 and 4. This analysis provides both the training and testing phases of models. It gives an overall assessment of the prediction performance, error, and reliability of each model, which are paramount in civil engineering applications. Starting with the R^2^ score, which measures how well each model explains the variance in the data, all models exhibit exceptionally high R^2^ values during the training phase: GOA-XGB gets 0.9941, SHO-XGB gets 0.9943, and LSA-XGB gets a slightly lower score of 0.9912. These values indicate that each model accounts for about 99% of the variability of the shear strength data and thus provides a good fit to the training dataset.

**Table 3:** Performance analysis during the training phase.

Metrics	GOA-XGB	SHO-XGB	LSA-XGB
R^2^ score	0.9941	0.9943	0.9912
RMSE	0.0104	0.0102	0.0127
MAE	0.0069	0.0071	0.0089
a20 Index	0.0008	0.0008	0.0009
WI	0.9985	0.9986	0.9978
VAF	99.4117	99.4281	99.1161
U95	0.0010	0.0010	0.0012

**Table 4:** Performance analysis during the testing phase.

Metrics	GOA-XGB	SHO-XGB	LSA-XGB
R^2^ score	0.9570	0.9594	0.9802
RMSE	0.0318	0.0309	0.0306
MAE	0.0210	0.0212	0.0208
a20 Index	0.0018	0.0023	0.0011
WI	0.9886	0.9891	0.9897
VAF	95.6950	95.9425	96.0307
U95	0.0059	0.0057	0.0057

Nonetheless, the 0.001 difference in R^2^ scores of the LSA-XGB may imply that it is less likely to over-fit compared to the training phase, since tested again during the testing phase. In the testing phase, coefficients of determination, R^2^ scores, decrease a bit, which is normal because models work with unknown data. Despite this decrease, the R^2^ values remain high: The R^2^ score of GOA-XGB is 0.9570, SHO-XGB is 0.9594, and the best R^2^ score is achieved by LSA-XGB with 0.9802. This high accuracy can be attributed to the LSA feature to efficiently explore the hyperparameter space of the XGB model without exaggerating the training data^54^. This shows that LSA-XGB outperforms the other models in terms of generalization, especially because the model complexity is well-balanced with the data fitting capability. The result showed enhanced prediction capacity compared to previous existing research works^14^. The testing phase results indicate that LSA-XGB can be the most suitable model for practical use when it is crucial to predict the results of new data. Other evaluation criteria that show the accuracy of each model are the RMSE and MAE. In the training phase, the RMSE values are very low for GOA-XGB at 0.0104, SHO-XGB at 0.0102, and LSA-XGB at 0.0127. This pattern is consistent with the MAE values: The results show that GOA-XGB has the lowest error at 0.0069, followed by SHO-XGB at 0.0071, while LSA-XGB has a slightly higher error at 0.0089. These low error values are able to substantiate the fact that the models yield near-correct predictions in the training phase. In the testing phase, RMSE and MAE values increase slightly, which is the expected drop in performance on new data. GOA-XGB has an RMSE of 0.0318 and an MAE of 0.0210; SHO-XGB has an RMSE of 0.0309 and an MAE of 0.0212; and LSA-XGB has slightly better results with an RMSE of 0.0306 and an MAE of 0.0208. The results of the testing phase indicate that LSA-XGB has a more appropriate level of generalization because the corresponding RMSE and MAE values are lower.

Further supporting the models’ credibility and stability are the A20 index, W(I), VAF, and U95 measures. The a20 index, which quantifies the proportion of predictions that are within a 20% range, is very small in the training set at about 0.0008 and slightly higher in testing. Such a low a20 index shows that the models’ predictions are very near reality and are quite precise. The WI values, which are near 1.0 for both training (0.9985) and testing (0.9886) sets, showed that the models have good conformity with the observed values and that the models are consistent. VAF values are above 95% for both phases, indicating that the models are good at reproducing almost all the data variability, with training VAFs peaking at 99.4%. The low U95 values in both phases suggest that there is little variability in the prediction, which is beneficial for the application of the models. Therefore, the high R^2^ values, low RMSE and MAE, high WI and VAF, and low U95 indicate that GOA-XGB, SHO-XGB, and LSA-XGB are very efficient in predicting the shear strength of UHPC beams. Even in the training phase, GOA-XGB and SHO-XGB have a high accuracy, but in the testing phase, LSA-XGB has a higher R^2^ score, fewer errors, and thus is the ideal model for use in practice. These findings demonstrate the effectiveness of hybrid machine learning models in improving structural prediction reliability in civil engineering, offering dependable tools to enhance UHPC design, material efficiency, and safety.

**Fig. 4:**
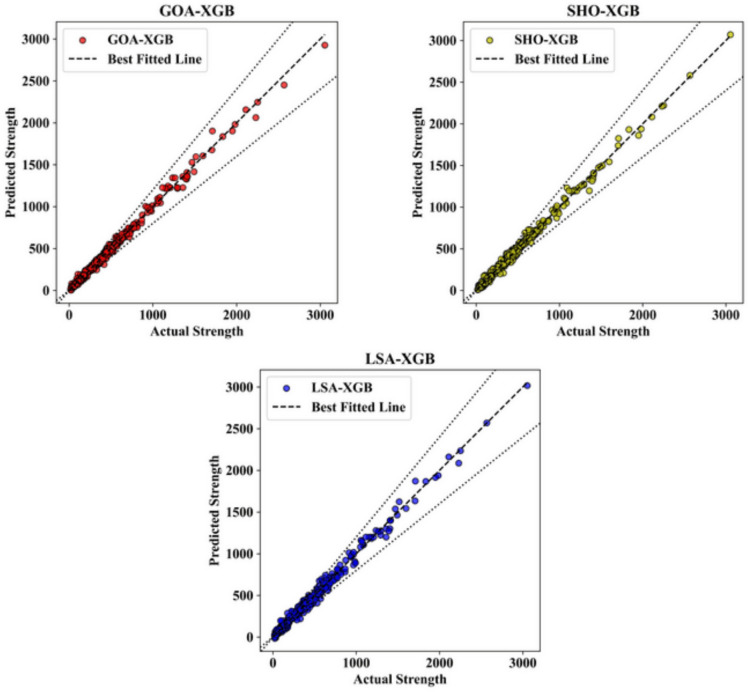
Actual vs. Predicted plot in training datasets.

**Fig. 5:**
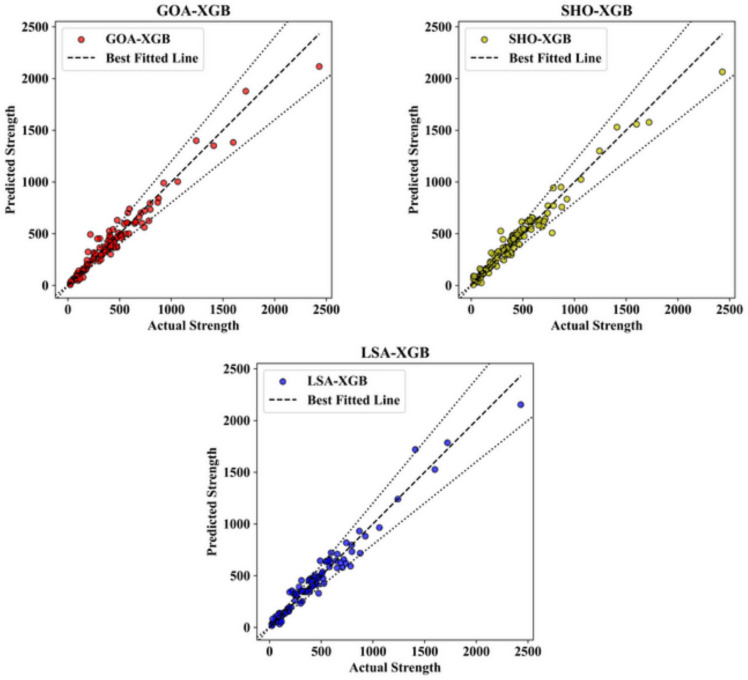
Actual vs. Predicted plot in testing datasets.

Further, Figs. 4 and 5 illustrate scatter charts of the measured shear strength (Vu, test) as well as the shear strength predicted by the ML models (Vu, pred). In these plots, the horizontal axis represents the test values, and the vertical pace shows the Vu and pred values. The greater the accordance of the dots with the black diagonal line that corresponds to y = x, the better the correspondence of the ML model to the distribution of the effect of input factors on the predicted shear strength. Notably, when comparing Figs. 4 and 5, most predicted points from all ML models for the test dataset closely align with the y = x line, as all models were optimized on the training dataset using different techniques. This means that for all the ML models generated, we can confirm that, indeed, they fit the data. In Fig. 4, which presents the results for the training datasets, the predicted strength values are closely aligned with the actual strength values across all three models. This strong correlation is visually apparent through the tight clustering of data points around the best-fit line, indicating a high level of predictive accuracy. For the GOA-XGB model, represented by red data points, most predicted values closely follow the actual strength values, with minimal deviation. Only a small number of points stray from the best-fit line, suggesting that the model is well-tuned to the training data.

The SHO-XGB model, shown in yellow, also displays a strong fit, although some deviations are observed at higher strength values. Despite these minor variances, the majority of data points remain close to the best-fit line, indicating that the SHO-XGB model performs reliably on the training data. Meanwhile, the LSA-XGB model, represented by blue points, demonstrates the highest level of accuracy among the three models. The predicted values almost perfectly follow the actual strength values, with very few points deviating from the best-fit line, showing that this model has been finely tuned to the characteristics of the training data. In Fig. 5, which illustrates the performance of these models on the testing datasets, the overall trends remain consistent. However, there is a slight increase in variability when compared to the training data. The GOA-XGB model, again represented by red points, shows a strong correlation between predicted and actual strength values. However, a few more deviations are noticeable in the higher strength ranges, indicating a slightly reduced accuracy when applied to unseen data. Nevertheless, the majority of points still align closely with the best-fit line, demonstrating the model’s strong predictive capability even on the testing set.

For the SHO-XGB model, displayed in yellow, the predicted values continue to follow the actual strength values closely, though the variance in mid-range strength values is more pronounced compared to the training set. This suggests that while the model generalizes well to unseen data, it may show a slight decline in performance at certain strength ranges. However, it still maintains a reasonably strong overall correlation, as most data points cluster near the best-fit line. The LSA-XGB model, represented by blue dots, again shows the best performance with the least error between the predicted and actual values, including the testing set. The majority of the predicted values are still quite accurate in terms of the actual strength values, and the points are clustered around the regression line. This agreement across both the training and testing sets shows that the LSA-XGB model has excellent out-of-sample prediction ability and is ideal for usage in both the training and testing datasets. In general, the comparison between Figs. 4 and 5 shows that the XGB models are quite reliable. Although the accuracy is slightly lower when the model is applied to the test data, the models, especially LSA-XGB, remain quite effective. The figures taken all together underscore the models’ capacity for identifying the structure of the data in the training set and then reproducing that performance in the testing set – a key factor for successful use in practical applications. The LSA-XGB model, for instance, demonstrates a high level of stability in the obtained accuracy across both datasets, thus being the most suitable model for strength values forecasting in this analysis.

### Model evaluation and validation techniques

**Fig. 6:**
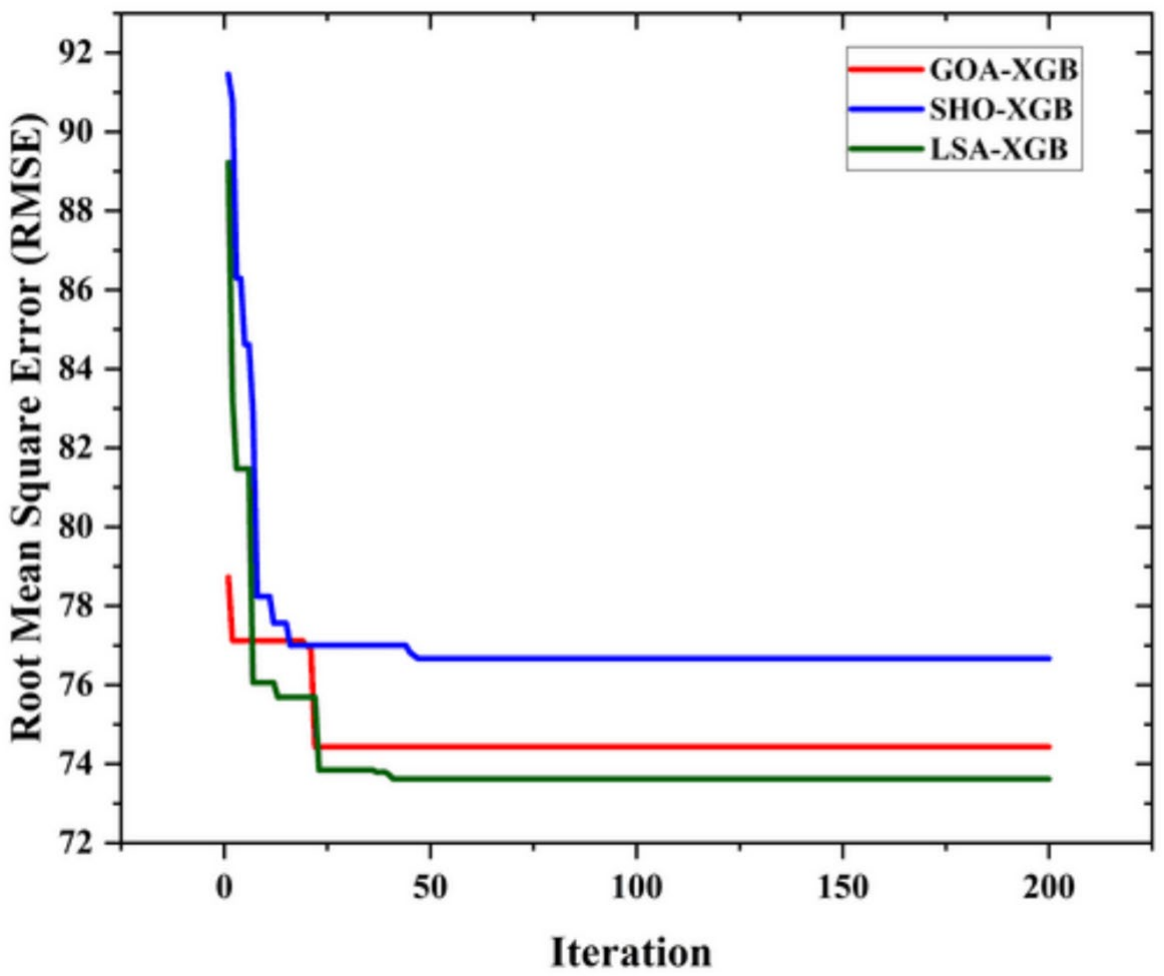
Convergence curve of all employed model.

Figure 6 shows the RMSE convergence of the three models used in this study, namely GOA-XGB, SHO-XGB, and LSA-XGB. The first axis on the graph is the number of iterations, while the second one is the RMSE values. The graph gives information on how each model takes to converge and obtain the minimum error that it can perform during training. The GOA-XGB model, depicted by the red line, has the fastest rate of convergence to an RMSE of about 74 in a few iterations. The sharp decrease in RMSE indicates that the model quickly learns and adjusts its predictions during the initial stages of training, finding the optimal solution efficiently. The SHO-XGB model is shown in the blue line, and it is observed that it has a higher RMSE at the beginning of the iterations than the other models, but it gradually reduces after some iterations. However, in the final analysis, the RMSE is approximately 76, which shows that the model provides a slightly less precise solution than the GOA-XGB and LSA-XGB. The LSA-XGB model, shown in green, also exhibits a similar convergence trend to the GOA-XGB model but with a slightly higher initial RMSE. Following a sharp fall in RMSE, the LSA-XGB model attains the minimum error of about 73, suggesting that it is the most efficient of the three models in terms of this metric.

The convergence differences between GOA-XGB, SHO-XGB, and LSA-XGB are because the former two models have a more efficient exploration–exploitation strategy. The GOA-XGB quickly decreased the RMSE by presetting on good solutions at the beginning of the process, which reduces the convergence time, albeit slightly reducing the final solution’s accuracy^52,60^. SHO-XGB is a very slow learning algorithm, which tries to avoid RMSE minimum’s pitfalls of getting caught up in local minima and thus has a slightly higher RMSE^53,61^. LSA-XGB can achieve both the highest exploration and the highest refinement, the fastest convergence and the lowest RMSE, thus being the most accurate of the three^54^.

In summary, Fig. 6 shows that all three models can converge, but the LSA-XGB model is the most efficient in terms of RMSE, followed by the GOA-XGB model. The SHO-XGB model, although still effective, exhibits a higher error value, indicating that there is potential for further improvement. From the above convergence behaviour comparison, it is now clear that different models have unique efficiency and accuracy while training.

**Fig. 7:**
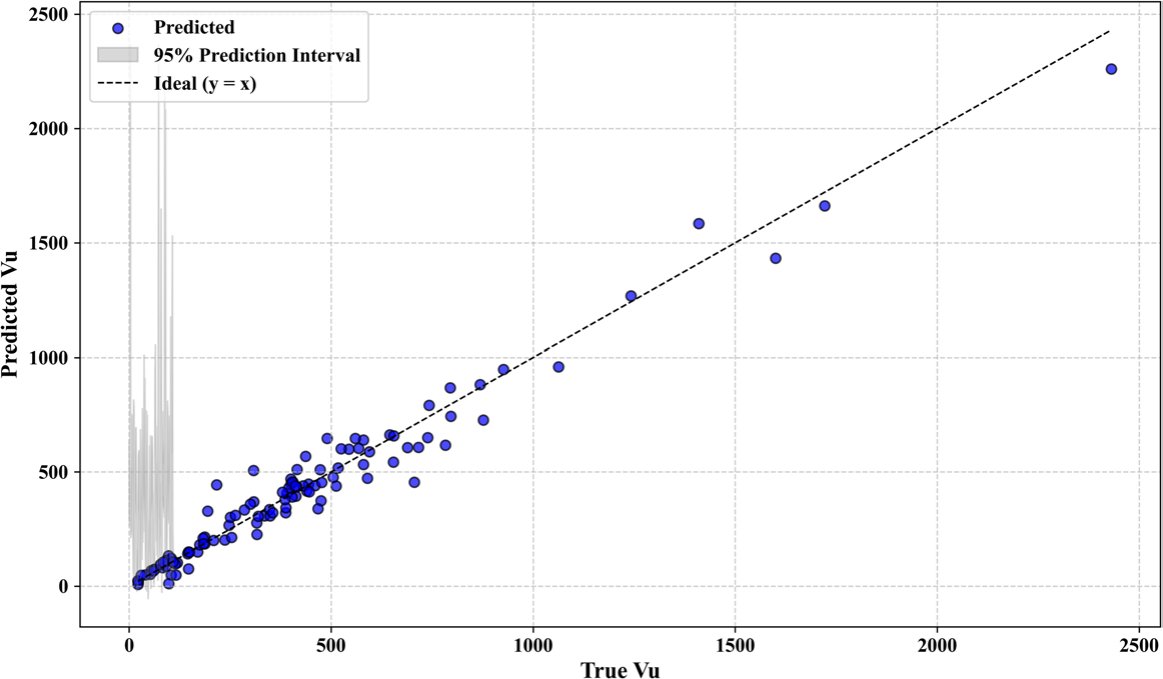
Bootstrap residual resampling.

**Fig. 8:**
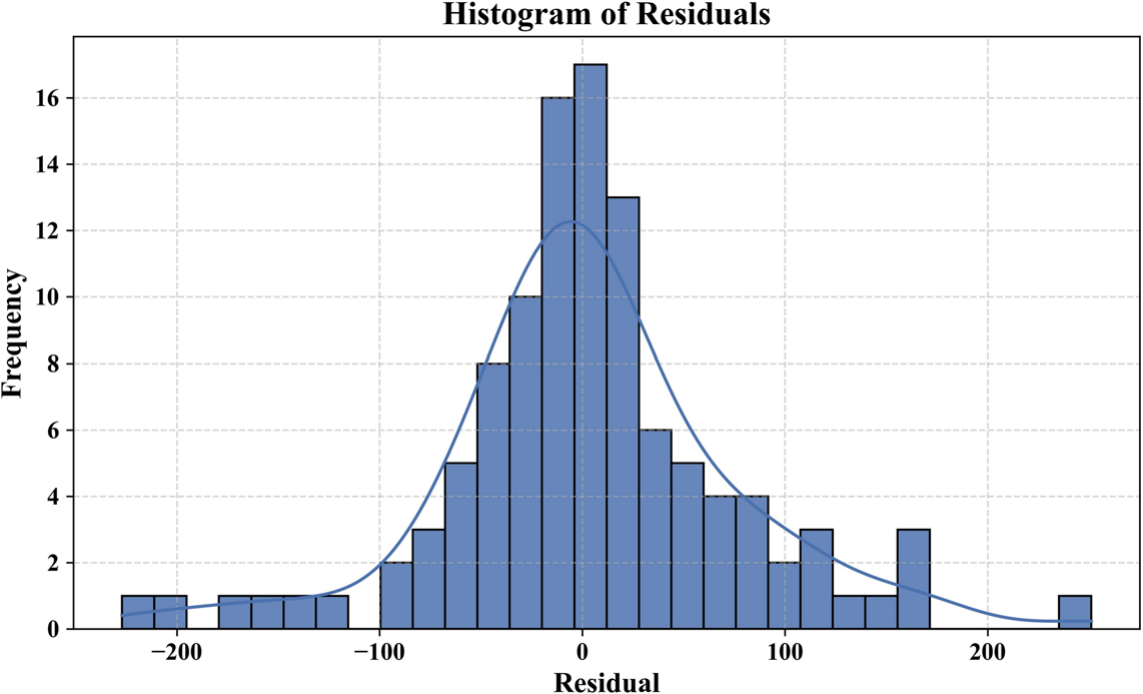
Model residual error of the best model.

Additional statistical analysis was carried out to confirm the robustness and generalizability of the used models. The bootstrap residual resampling result of the LSA-XGB model, as shown in Fig. 7 indicates a good fit between the predicted and true value of shear strength with a small 95 percent prediction interval. This suggests that the model is quite stable and substrata by biased with resampling. The histogram of the residuals in Fig. 8 shows that they are normally distributed around zero, indicating that the errors are random and that the model is not too seriously overfitted.

**Fig. 9:**
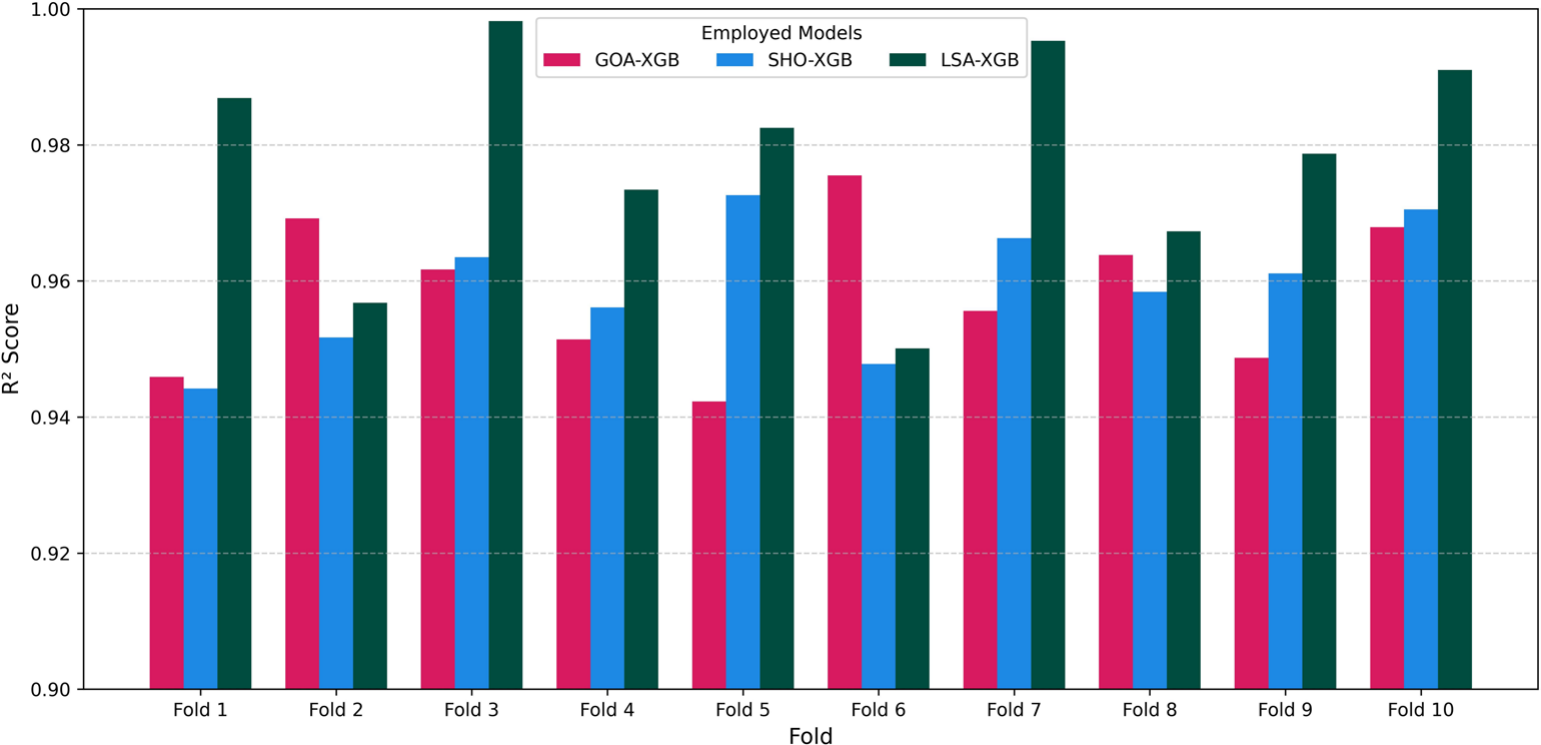
Cross validation of employed models in tenfold.

Lastly, Fig. 9 indicates the tenfold cross-validation of GOA-XGB, SHO-XGB, and LSA-XGB. Both LSA-XGB and the other methods have high values of R^2^ in all folds, showing that they have good generalization performance. These tests support the reliability, accuracy, and resilience of the intended models to unexpected data division and random resampling.

### Data visualization

**Fig. 10:**
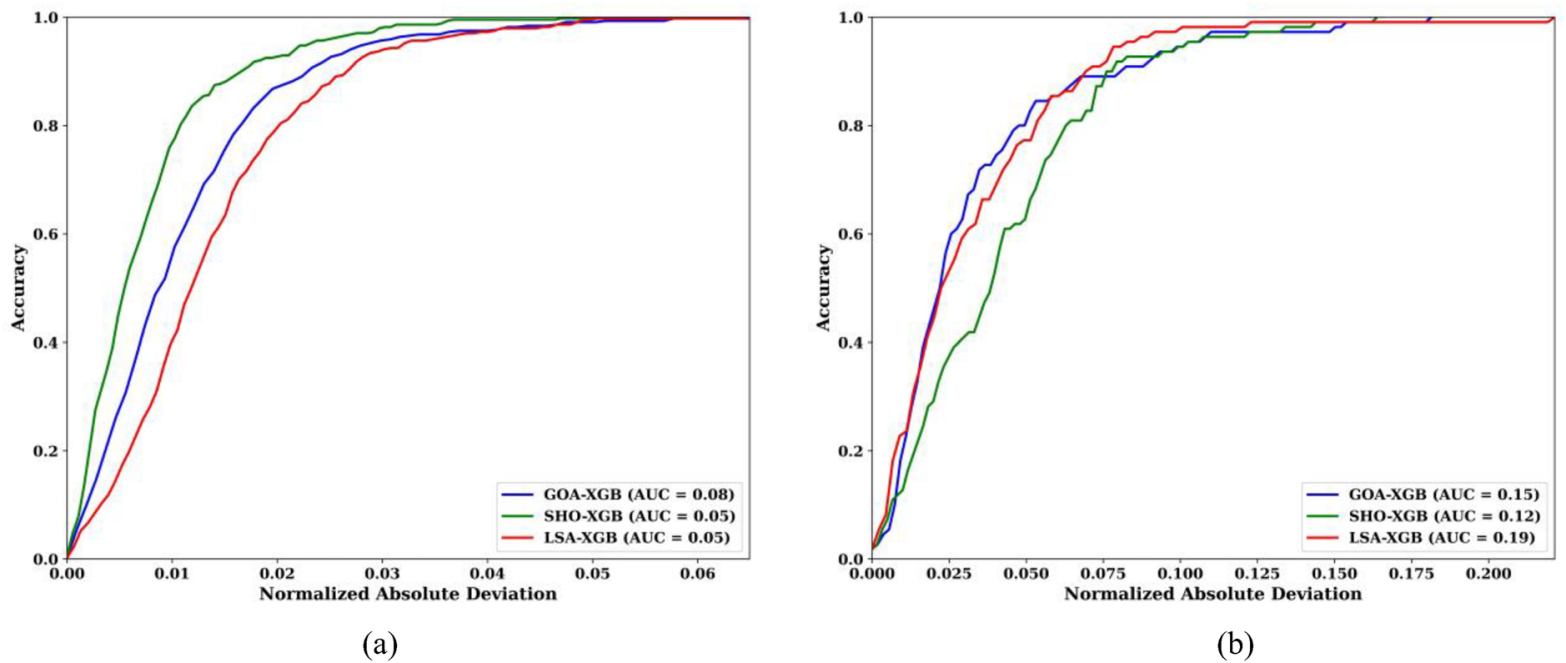
Regression Error Characteristics (REC) Curve (**a**) Training set (**b**) Testing Set.

Figure 10 presents the Regression Error Characteristic (REC) curves for the GOA-XGB, SHO-XGB, and LSA-XGB models on both training and testing datasets. These curves provide a visual comparison of the accuracy of each model as a function of normalized absolute deviation. The x-axis shows the normalized absolute deviation, while the y-axis represents accuracy. The Area Under the Curve (AUC) is included as a measure of the overall performance for each model. In Fig. 10a, which depicts the performance on the training dataset, the LSA-XGB model (green curve) demonstrates superior performance with the highest AUC of 0.905, indicating that this model has the best accuracy for predicting training data. The SHO-XGB model (blue curve) follows with an AUC of 0.885, showing good accuracy but falling slightly short of LSA-XGB. The GOA-XGB model (red curve) has an AUC of 0.868, making it the least accurate of the three on the training set but still within acceptable performance limits.

In Fig. 10b, the REC curves for the testing dataset reveal a similar trend, although the AUC values are slightly lower across all models. The LSA-XGB model remains the most accurate with an AUC of 0.819, followed closely by the SHO-XGB model with an AUC of 0.812. The GOA-XGB model, with an AUC of 0.815, also performs well, though all models show a small decrease in accuracy when applied to the testing data compared to the training data. The AUC differences stem from the fact that each model uses a different optimization approach. LSA-XGB outperforms all other models in terms of AUC because it finds a golden path in exploration and focused refinement, whereby it avoids many errors and can learn well from testing data. SHO-XGB has a wider scope but is not as precise when it comes to tuning, hence a slightly lower accuracy. GOA-XGB has a high convergence rate, which is good, but at the cost of the final accuracy, resulting in the lowest AUC. Therefore, LSA-XGB is the most reliable for accurate predictions with minimal deviation due to the balanced approach. The REC curves clearly illustrate the comparative effectiveness of each model in reducing prediction errors. A higher AUC indicates better overall performance, with the LSA-XGB model consistently demonstrating the best results on both training and testing datasets. These results provide strong evidence that the LSA-XGB model is the most reliable for making accurate predictions with minimal deviation.

### Model explainability

Explaining how a machine learning model arrives at its predictions is essential for interpreting results, especially in complex models. SHAP provides a robust framework for assessing the contribution of each feature to the model’s output. Figures 8, 9, and 10 collectively offer a deep dive into model explainability by utilizing SHAP values to illustrate the impact of individual features on the predictions. Figure 8 displays the Mean Absolute SHAP Value Plot, which ranks the features based on their average SHAP values, showing their overall importance in influencing the model’s predictions. Here, the feature Ac stands out with the highest mean SHAP value of 193.27, indicating that it has the strongest effect on the model’s decisions. Other features like m (109.60) and pf (42.66) also play significant roles, but with a lesser impact compared to Ac. Features at the lower end of the scale, such as lf (2.61) and bf2 (1.94), have minimal influence on the model’s predictions. This ranking helps to identify the key drivers behind the model’s outputs and gives insight into which features are most influential.

**Fig. 11:**
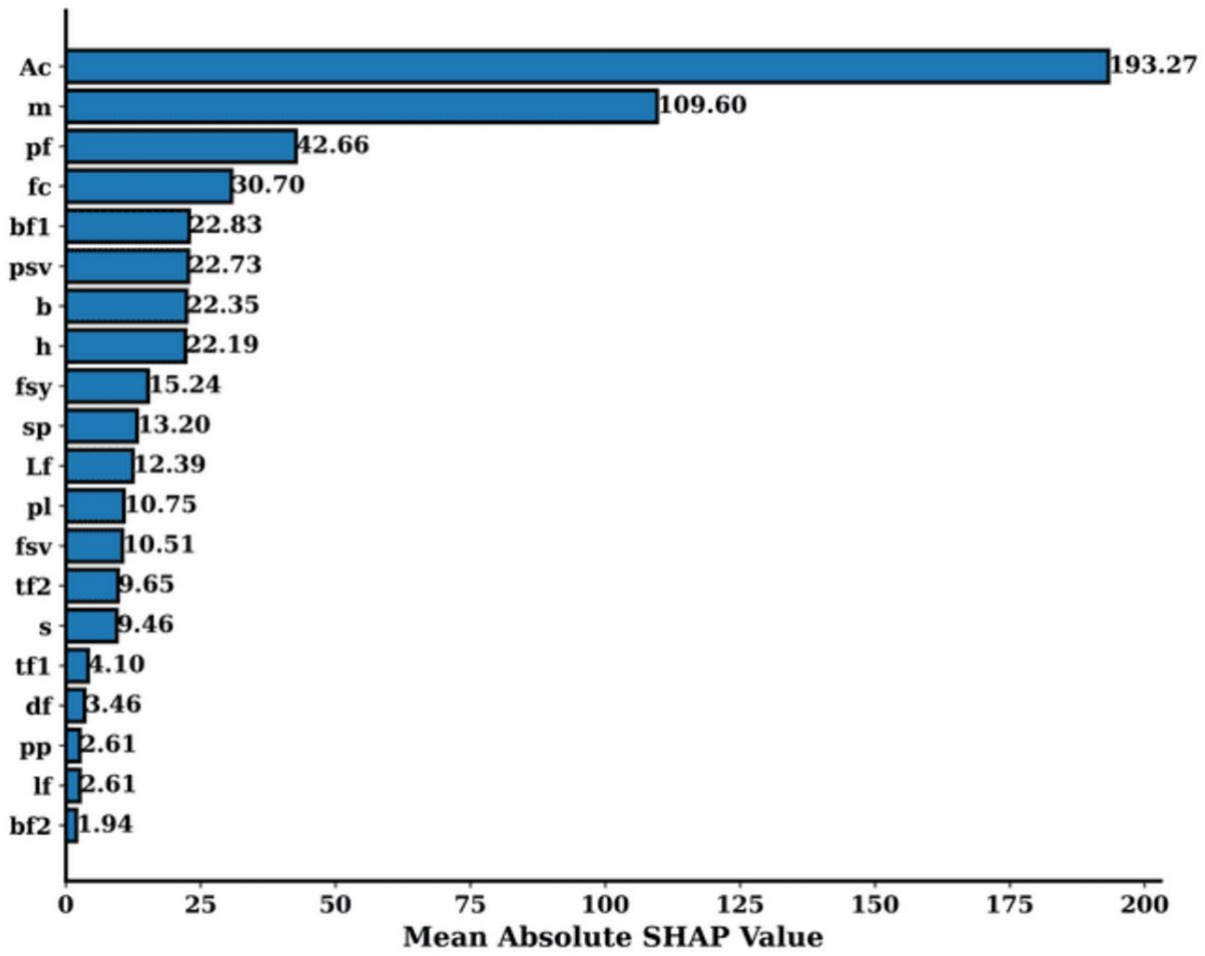
Mean absolute SHAP value plot.

Figure 11 goes further by presenting a SHAP Summary Plot, which visualizes how each feature value affects the model’s predictions. The x-axis shows the SHAP values, and the color gradient (from blue to red) represents the range of feature values, where blue indicates lower values and red represents higher ones. For the feature Ac, a wide distribution of SHAP values is observed, showing both positive and negative impacts on the predictions. Higher values of Ac (red) generally push the predictions upwards, while lower values (blue) reduce the predictions. A similar pattern is observed for features like m and pf, where higher feature values are associated with increased SHAP values, indicating a positive influence on the predictions. This plot allows for a better understanding of not only which features are important but also how changes in feature values influence the model’s predictions.

**Fig. 12:**
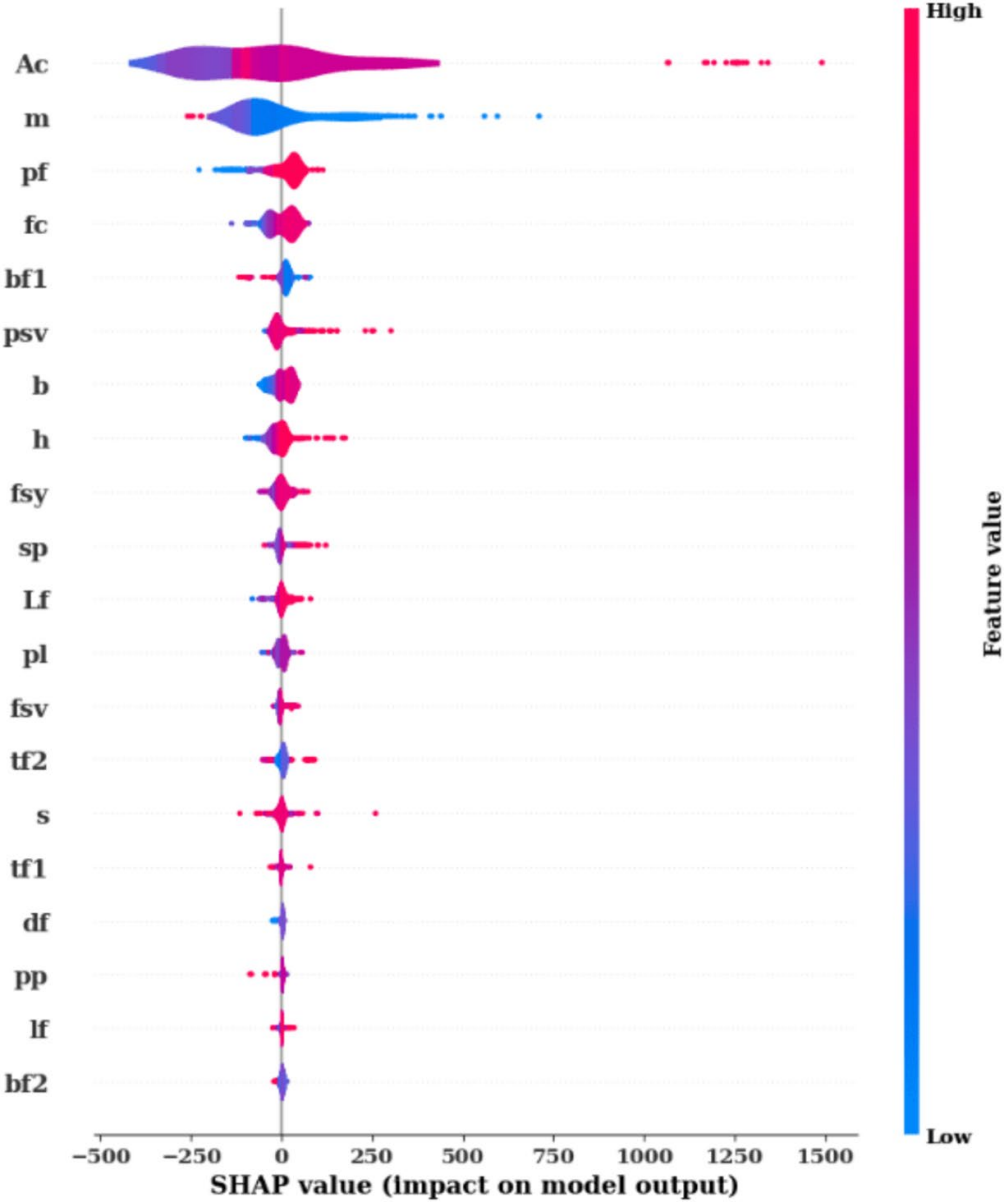
SHAP values.

**Fig. 13:**
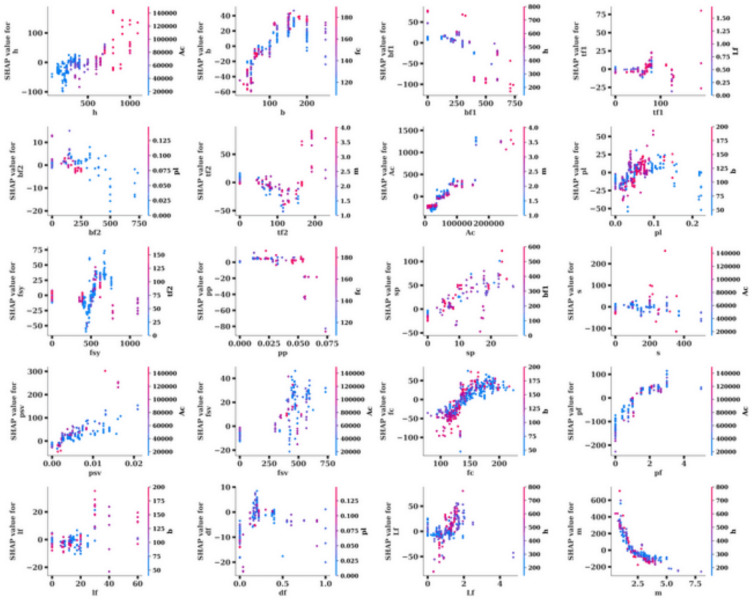
Model explainability using SHAP values.

Figure 12 takes a closer look at individual relationships between feature values and their SHAP values through a series of SHAP Scatter Plots. These scatter plots illustrate how each feature’s value directly affects its contribution to the model’s predictions. For instance, the feature Ac shows a strong positive correlation between higher values and increased SHAP values, suggesting that higher Ac values significantly boost the model’s predicted output. In contrast, features like pf demonstrate a negative correlation, where higher feature values lead to lower SHAP values and, consequently, lower predictions. Certain features, such as bf2 and df, exhibit smaller ranges of SHAP value changes, indicating that they play a less critical role in the overall prediction process. These plots provide detailed insights into the behavior of each feature and how its values influence the model’s output. In conclusion, Figs. 11, 12, and 13 provide a thorough analysis of model explainability using SHAP values. Figure 11 identifies the most important features, while Fig. 12 illustrates how variations in feature values impact predictions. Finally, Fig. 13 offers a closer look at the specific relationships between feature values and their contributions to the model’s output. Together, these figures provide a clear understanding of the model’s decision-making process and highlight which features are driving the predictions.

**Fig. 14:**
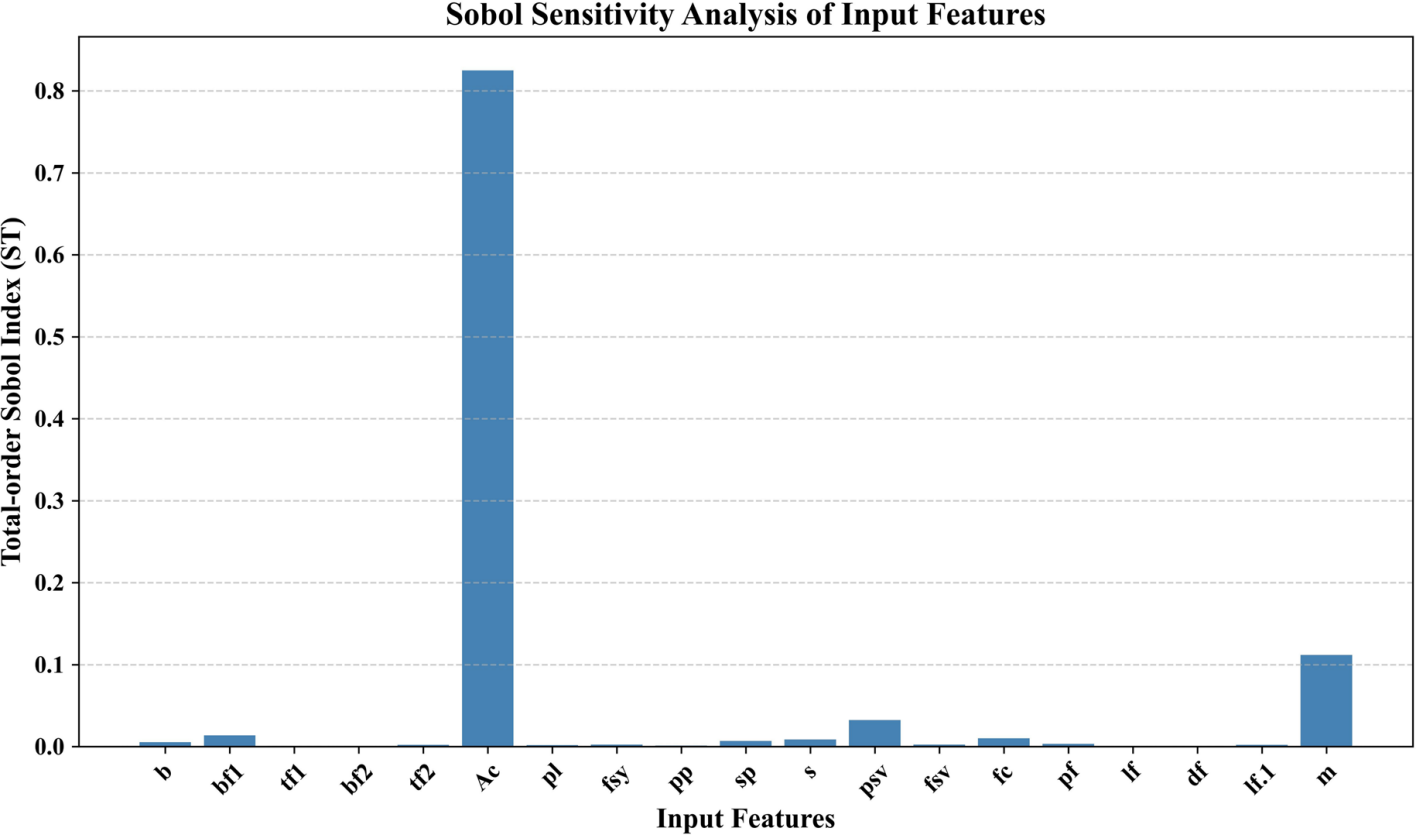
Sobol senstitivity analysis.

Besides the insights of the SHAP-based model, the Sobol global sensitivity analysis was also performed to measure the impact of input features on the model output. The total-order Sobol index indicates that Ac has a significant effect on the predicted shear strength (ST > 0.8), as shown in Fig. 14, and this confirms its dominant influence. m has a very small yet significant contribution, whereas the other features all have a very negligible influence. This homology of SHAP with Sobol outcomes increases the trust in the interpretation of feature importance and confirms that the decision-making process of the model is solid.

### Comparison with existing empirical equations

Various existing codes are present to determine beams’ shear capacity, developed based on experiments, theories, and assumptions. However, the shear mechanism is complex as numerous parameters are involved. All of these parameters are hard to include in a single equation. The standard parameters for the shear capacity are section size, axial compressive strength, axial tensile strength, transverse reinforcement, longitudinal reinforcement, fiber factor, etc. Since the existing codes are based on some assumptions, getting precise results is difficult, making it necessary to modify the existing equation or use other advanced techniques. This research utilizes existing codes, such as Model MC2010, EN, AFGC-2013, and EN 1992-1-1, to predict shear and determine the shear capacity of UHPC beams.

#### Fib model code for concrete structures 2010 (Model MC2010)

For determining the shear capacity of FRC beams, Model MC2010^62^ has provided an equation as mentioned in Eq. (28). This equation takes into account the contribution of the concrete from Eurocode 2. Parameters for his model include cylinder compressive strength, ultimate residual tensile strength, average tensile strength, average stress acting on the cross-section due to prestress and cross-sectional area.

28 $${\text{Vu}} = \left. {\left\{ {\frac{{0.18}}{{\gamma c}}k\left[ {100\rho s\left( {1 + 7.5\frac{{fFtuk}}{{fctk}}} \right)*fck} \right]^{{\frac{1}{3}}} + 0.15\sigma _{{cp}} } \right\}bwd} \right)$$

where,

K is the size effect factor = 1+$\sqrt{\frac{200}{d}}$  ≤ 2.

Here, d is the effective depth, ρs is the longitudinal reinforcement ratio Astbwd ρs\rho_sρs​ is the longitudinal reinforcement ratio, *f*_*ck*_​ is the compressive strength, *f*_*Ftuk*_​ is the ultimate residual tensile strength, and *f*_*cuk*_ ​ is the concrete’s tensile strength (all in MPa). *σ*_*cp*_​ represents prestress-induced stress, *b*_*w*_is the smallest width of the tension area, and ϒ_*c*_ (safety factor) is taken as 1. Incorporating *f*_*Ftuk*_​ enhances the matrix with fiber benefits.

#### French norm (AFGC-2013)

The French code AFGC-2013^63^ utilizes the truss model to determine the shear capacity of UHPC beams. The role of transverse reinforcement and fiber contribution is taken into consideration. The shear capacity is calculated by using Eq. (29).

29 $$V = V_{c} + V_{s} + V_{f}$$

where, *V*_*c*_ -shear capacity contribution due to concrete matrix, *V*_*s*_—shear capacity contribution due to transverse reinforcement, and *V*_*f*_—shear capacity contribution due to fiber.

The three components are calculated using the following Eqs. (30–32).

30 $$V_{c} = \frac{0.21}{{\Upsilon_{1} }}{\text{k}}_{2} {\text{f}}_{{\text{c}}}^{0.5} {\text{bd}}$$

where ϒ_1_ is the safety factor, k_2_ is the prestress coefficient, and b and d are the breath and effective depth, respectively.

31 $${\text{V}}_{{\text{s}}} = \frac{{A_{s} }}{s}zf_{y} cot\theta$$

where *A*_*s*_ is the area of the stirrup, z is the distance between the top and bottom longitudinal reinforcement, s is the spacing of the stirrup, θ is the angle between the diagonal compression and the bottom and the beam axis taken as 45 degrees, f_y_ is the yield strength of reinforcement.

32 $$V_{f} = \frac{{A_{b} \sigma_{Rd,f} }}{\tan \theta }$$

where, *A*_*b*_ is the cross-section of the concrete matrix and σ_Rd,f_ is the residual tensile strength.

#### European norm (EN 1992-1-1)

European norm (EN1992-1-1)^64^ takes into account the shear capacity provided by the concrete matrix and stirrup. Shear capacity is calculated using Eqs. (33) and (34) as given in:-

33 $$\begin{gathered} V_{s} = V_{Rd,c} + V_{Rd,s} \hfill \\ V_{Rd,c} = C_{(Rd,c)} k(100\rho f_{c} k)^{(1/3)} b_{w} d \hfill \\ \end{gathered}$$

where, *C*_*Rd,c*_ = 0.18/ϒ_c_ where ϒ_c_ is the partial safety factor, *k* = 1+$\sqrt{\frac{200}{d}}$ where d is the effective depth, *ρ* is the reinforcement ratio

34 $$V_{Rd,s} = \frac{{A_{sw} f_{ywd} z}}{s}\cot \theta$$

where, *f*_*ck*_ is the compressive strength of concrete (MPa), *b*_*w*_ = the width of the cross-section,

*A*_*sw*_ is stirrup sectional area, *z* = internal lever height taken as 0.9d, *s* is be spacing of the stirrup, and *θ* is the angle between the pressure bar and the longitudinal bar of the truss.

**Fig. 15:**
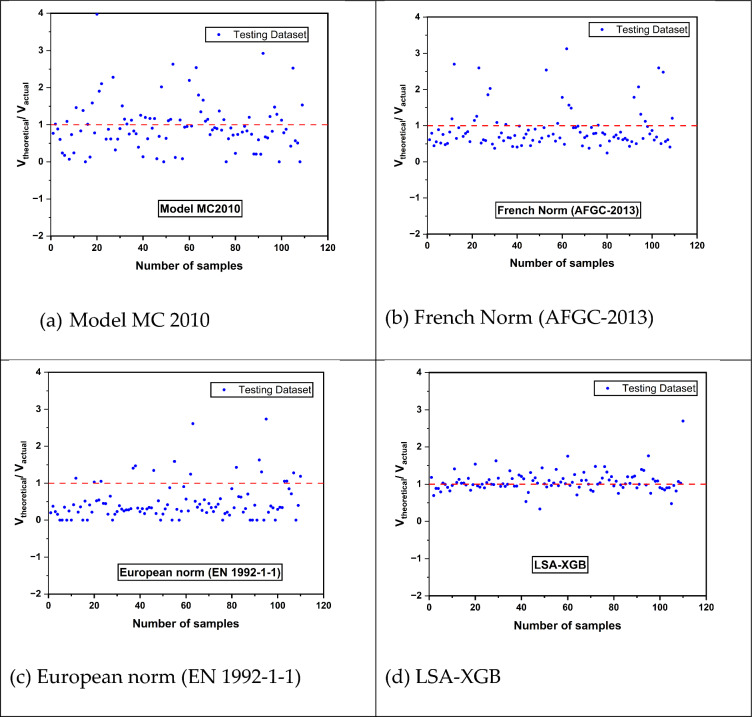
Results from existing shear capacity prediction models and comparison with the current study.

Figure 15 presents a comparison of the efficiency of the shear capacity prediction models for UHPC beams, including Model Code 2010 (MC2010), French Norm (AFGC-2013), and European Norm (EN 1992-1-1) with the advanced machine learning-based model LSA-XGB. The conventional models, MC2010 and AFGC-2013, mainly overestimate the shear strength, which may result in unsafe design considerations due to over-estimation of the UHPC beams’ performance. This overestimation is evident as their data points are consistently above the ideal 1:1 ratio, which means that the predicted values would be equal to the actual measurements in the most accurate way. On the other hand, EN 1992-1-1 tends to overemphasize the shear capacity as a general rule, which may be a more safety-oriented approach that helps to avoid overloading structures and, at the same time, may produce designs that are not sufficiently effective in terms of using materials, thus unnecessarily increasing structural reinforcement costs unnecessarily. The LSA-XGB model, utilizing a sophisticated XGB algorithm, shows a remarkable alignment of predictions with actual data, indicated by the tight clustering of points around the 1:1 ratio. This high accuracy demonstrates the possibility of using more sophisticated models for the prediction of structural performance and reliability in the design of structures, especially those made of UHPC, which has special mechanical characteristics. These results underscore the need for more accurate, machine learning-based models to be incorporated into design codes for high-consequence structures.

Table 5 shows the coefficient of regression (R^2^) and the theoretical to actual ratios of shear capacity. A high R^2^ value nearly 1, for instance, 0.9802 in LSA-XGB, shows that high predictability and ratio values closer to 1 imply more accuracy. The LSA-XGB model gives the highest overall performance with an R^2^ of 0.9802 and theoretical/actual ratio of 1.06, which suggests its predicted values are almost similar to the actual values. On the other hand, the other models, especially the European norm, have a large spread and lower accuracy, with the European norm model underpredicting, as evidenced by a ratio of 0.48. This analysis shows that there is a possibility of using machine learning techniques in structural engineering applications where accurate prediction of shear capacity is important, as compared to the use of traditional normative approaches.

**Table 5:** Comparison of different types of models.

Parameters	MC-2010	AFGC-2013	EN 1992–1-1	LSA-XGB
*R*^2^ score	0.5930	0.8408	0.5285	0.9802
*V*_*Theoretical*_*/V*_*Actual*_	1.19	1.12	0.48	1.06

The results of this study indicate that traditional models do not adequately capture the behaviour of UHPC beams, and future research should be directed toward refining or developing new shear capacity prediction methods that are more consistent with the unique structural characteristics of UHPC to ensure both safety and cost-effectiveness in design.

**Fig. 16:**
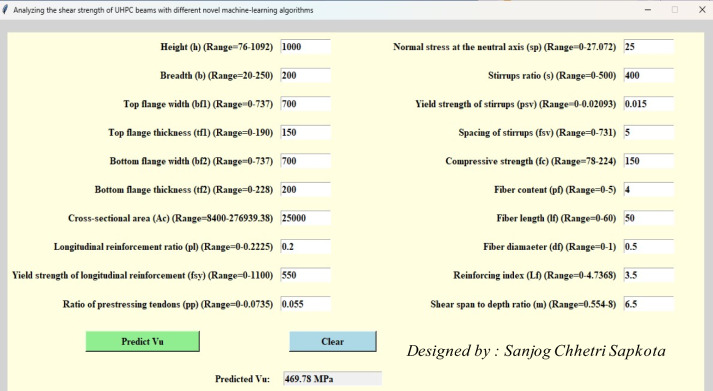
GUI for the prediction of shear strength of UHPC beam.

Since several parameters influence the shear behaviour of the UHPC beam, there is a critical issue in developing reliable and scalable modelling. To address this, an innovative GUI interface, as shown in Fig. 16**,** is integrated using the best-adopted model in the study. GUI development aims to generalize the state-of-the-art implementation of developed models for engineers and researchers. This framework can be helpful in the estimation of the shear strength of beams using their data. Further, engineers can make quick decisions by understanding the parameters influencing shear strength in real time for reliable problem-solving. This study integrates numerous libraries, including Pickle and Tkinter of the Python programming language. This interface can be scalable by integrating application-based modules into structural software.

## Conclusions

This research utilizes advanced machine learning techniques to predict the shear strength of UHPC beams. Combining XGB with metaheuristic algorithms, including GOA, SHO and LSA, this study has illustrated how high-level modelling techniques are used in predicting shear capacity. The vast collection of data has been a rich source for the benchmarking of these models and has been derived from various literature surveys containing a plethora of influential parameters. Additionally, this research evaluates the accuracy of existing codes. The following points summarize the key findings of this research:

1. The models demonstrated outstanding predictive performance; LSA-XGB had the highest testing R^2^ (0.9802), followed by SHO-XGB (0.9594) and GOA-XGB (0.9570). With SHO-XGB at 0.9943, GOA-XGB at 0.9941, and LSA-XGB at 0.9912, all models obtained extremely high R^2^ values during the training phase, demonstrating their ability to capture the variation in UHPC shear strength.
2. The accuracy of the structural predictions was highlighted by the models’ low error rates across all investigations. In testing, SHO-XGB achieved an RMSE of 0.0309 and MAE of 0.0212; GOA-XGB had an RMSE of 0.0318 and MAE of 0.0210; and LSA-XGB achieved the lowest errors, with an RMSE of 0.0306 and MAE of 0.0208. Additionally, throughout the simulation, all models had high VAF values above 95%, demonstrating the accuracy of the models’ predictions.
3. The LSA-XGB model had a better fit on the testing data with an R^2^ of 0.9802. In comparison, existing empirical equations yielded lower R^2^ values: 0.5930 for MC-2010, 0.8408 for AFGV-2013, and 0.5285 for EN 1992-1-1. These results show that the proposed LSA-XGB model is more accurate than the conventional methods used in the literature.
4. The comparison of various models, MC-2010, AFGC-2013, EN 1992-1-1, and the LSA-XGB hybrid model, revealed the following theoretical-to-actual prediction value ratios: 1.19, 1.12, 0.48, and 1.06, respectively. These results indicate that the LSA-XGB model provides predictions closer to actual values, outperforming traditional empirical equations.
5. The theoretical to actual prediction value ratios for the various model types—MC-2010, AFGC-2013, EN 1992-1-1, and LSA-XGB—were 1.19, 1.12, 0.48, and 1.06, respectively. This indicates that the hybrid prediction model that was developed outperformed empirical equations.
6. The application of GOA, SHO, and LSA algorithms in conjunction with XGB has successfully improved learning capabilities and optimized parameter tuning, demonstrating the potential of nature-inspired algorithms to tackle challenging engineering problems.
7. The findings indicate significant potential for using these models to improve the design, assessment, and management of UHPC structures, aligning with and potentially advancing current engineering codes and practices. The models provide a basis for revising existing design codes, which often do not fully account for the unique properties of UHPC.
8. Shapley Additive Explanations (SHAP) was employed, which improved the interpretability of employed ML models, vital for their broader acceptance and application in practical engineering scenarios.
9. Following model selection, a graphical user interface (GUI) was created for LSA-XGB to help engineers estimate UHPC shear strength. When designing UHPC beams, this GUI can be regarded as a useful, economical, and effective tool that enhances decision-making and aids in material optimization.

In summary, this study accurately predicted the shear strength of UHPC beams using an advanced machine learning algorithm. Understanding shear behavior and improving prediction model accuracy are still challenges. Despite the hybrid model’s high accuracy, there are still a number of areas that could be explored and improved. These include improving the incorporation of machine learning models into real-world engineering workflows, investigating the impact of extra variables, and tackling the constraints of diverse datasets.

## Limitations of the current study and future research directions

The primary limitation of this study lies in the relatively smaller dataset that was used to develop prediction models. More reliable and useful insights might be obtained by enlarging the dataset to include samples from various geographical locations with different binding materials and material compositions. The dataset used was entirely sourced from existing experimental literature, which, while comprehensive, may limit the generalizability of the model to novel concrete mix designs, uncommon cross-sectional geometries, or structural elements subjected to dynamic or fatigue loading. Although the current study aimed at the development of accurate machine learning models to predict the shear strength of UHPC beams, it is desirable that in the future, this research be continued by including the measures of uncertainty and risk-informed models in an effort to aid more visibly in structural design-making. Additionally, this study employs a hybridized ensemble model, which is particularly good for smaller datasets. However, it becomes inefficient and computationally expensive for larger datasets. Future research can explore the application of deep neural network modelling to overcome this limitation. A significant limitation of the ML model is developing a predictive equation. Future research can focus on utilizing modelling techniques that are capable of establishing a robust correlation between input and output parameters. Likewise, it is frequently difficult to comprehend how features interact in complex models, which makes it more difficult to interpret their contributions. Moreover, while SHAP was employed for model interpretability due to its theoretical rigor and additive consistency, comparing it with other model explanation techniques, such as permutation feature importance and LIME, could further validate its selection. Interpretation based on domain knowledge representation is, therefore, crucial. Therefore, results from different explainable models are evident to corroborate a finding. Furthermore, with an increase in datasets, real-time approaches become essential to ensure efficient processing and prediction. Future research should focus on integrating real-time data processing techniques with ML models to enhance their scalability and practical applicability in dynamic environments. The future development should also include investigating higher-order feature selection and multicollinearity alleviation methods like Recursive Feature Elimination (RFE) and Variance Inflation Factor (VIF) analysis to achieve better robustness and interpretability of a model.

## Supplementary Information

Supplementary Information.

## Data Availability

The datasets generated during and/or analysed during the current study are available from the corresponding author on reasonable request.

## Abbreviations

UHPC: Ultra-high-performance concrete
ML: Machine learning
NIA: Nature-inspired algorithm
XGB: Extreme gradient boosting
GOA: Giant armadillo optimization
SHO: Spotted hyena optimization
LSA: Leopard seal optimization
SHAP: Shapley additive explanations
AI: Artificial intelligence
DT: Decision trees
SVM: Support vector machines
kNN: K-nearest neighbours
ANN: Artificial neural networks
KDE: Kernel density estimator
GBM: Gradient boosting machine
RFE: Recursive feature elimination
R^2^: Coefficient of regression
RMSE: Root mean squared error
MAE: Mean absolute error
VAF: Variance accounted for
GUI: Graphical user interface
VIF: Variance inflation factor
Ac: Area of concrete
m: Stirrup spacing
pf: Fiber pull-out force
bf1: Flange width 1
bf2: Flange width 2
tf1: Flange thickness 1
tf2: Flange thickness 2
Vu: Ultimate shear strength
h: Overall section height
b: Width of section
s: Stirrup spacing
pp: Prestressing strands
sp: Shear plane
psv: Prestressing steel volume
fsv: Shear strength of steel
fsy: Yield strength of steel
fc: Compressive strength of concrete
df: Diameter of fiber
lf: Length of fiber
