# Supplementary material for: Enhancing shear strength predictions of UHPC beams through hybrid machine learning approaches
Source: Sci Rep. 2025 Aug 2;15:28259. doi: 10.1038/s41598-025-13444-y (PMC12318006; doi:10.1038/s41598-025-13444-y)
Supplement: Supplementary file 1 — Supplementary Information. [file 41598_2025_13444_MOESM1_ESM.docx]

**Supplementary material-1 (Data Set)**

|  | | | | | | | | | | | | | | | | | | | | |
| --- | --- | --- | --- | --- | --- | --- | --- | --- | --- | --- | --- | --- | --- | --- | --- | --- | --- | --- | --- | --- |
| **h** | **b** | **bf1** | **tf1** | **bf2** | **tf2** | **Ac** | **ρl** | **fsy** | **ρp** | **σp** | **s** | **ρsv** | **fsv** | **fc** | **ρf** | **lf** | **df** | **λf** | **m** | **Vu** |
| 200 | 150 | 0 | 0 | 0 | 0 | 30000 | 0.0134 | 421.4 | 0 | 0 | 75 | 0.014 | 466.6 | 138.5 | 2 | 13 | 0.2 | 1.7 | 1.5 | 194.5 |
| 200 | 150 | 0 | 0 | 0 | 0 | 30000 | 0.0209 | 421.4 | 0 | 0 | 75 | 0.014 | 466.6 | 138.5 | 2 | 13 | 0.2 | 1.7 | 1.5 | 298.8 |
| 200 | 150 | 0 | 0 | 0 | 0 | 30000 | 0.0253 | 421.4 | 0 | 0 | 75 | 0.014 | 466.6 | 138.5 | 2 | 13 | 0.2 | 1.7 | 1.5 | 388.2 |
| 200 | 150 | 0 | 0 | 0 | 0 | 30000 | 0.0253 | 421.4 | 0 | 0 | 75 | 0.0067 | 466.6 | 138.5 | 2 | 13 | 0.2 | 1.7 | 1.5 | 375.6 |
| 200 | 150 | 0 | 0 | 0 | 0 | 30000 | 0.0134 | 421.4 | 0 | 0 | 100 | 0.014 | 466.6 | 138.5 | 2 | 13 | 0.2 | 1.7 | 1.5 | 363.8 |
| 350 | 200 | 0 | 0 | 0 | 0 | 70000 | 0.148057 | 419.2 | 0 | 0 | 0 | 0 | 412 | 164 | 2 | 13 | 0.2 | 1.7 | 2.8 | 670 |
| 350 | 200 | 0 | 0 | 0 | 0 | 70000 | 0.148057 | 419.2 | 0 | 0 | 0 | 0 | 412 | 174.9 | 1.5 | 13 | 0.2 | 1.7 | 2.8 | 739 |
| 350 | 200 | 0 | 0 | 0 | 0 | 70000 | 0.148057 | 419.2 | 0 | 0 | 0 | 0 | 412 | 118.5 | 0 | 13 | 0.2 | 1.7 | 2.8 | 29.5 |
| 400 | 200 | 0 | 0 | 0 | 0 | 80000 | 0.126348 | 419.2 | 0 | 0 | 0 | 0 | 412 | 164 | 2 | 13 | 0.2 | 1.7 | 2.8 | 770 |
| 400 | 200 | 0 | 0 | 0 | 0 | 80000 | 0.126348 | 419.2 | 0 | 0 | 0 | 0 | 412 | 174.9 | 1.5 | 13 | 0.2 | 1.7 | 2.8 | 742.5 |
| 400 | 200 | 0 | 0 | 0 | 0 | 80000 | 0.126348 | 419.2 | 0 | 0 | 0 | 0 | 412 | 118.5 | 0 | 13 | 0.2 | 1.7 | 2.8 | 247.5 |
| 300 | 200 | 0 | 0 | 0 | 0 | 60000 | 0.049087 | 425.1 | 0 | 0 | 0 | 0 | 412 | 126.1 | 0 | 0 | 0 | 0 | 1.2 | 783 |
| 300 | 200 | 0 | 0 | 0 | 0 | 60000 | 0.049087 | 425.1 | 0 | 0 | 0 | 0 | 412 | 126.1 | 0 | 0 | 0 | 0 | 1.8 | 317 |
| 300 | 200 | 0 | 0 | 0 | 0 | 60000 | 0.049087 | 425.1 | 0 | 0 | 0 | 0 | 412 | 126.1 | 0 | 0 | 0 | 0 | 3.1 | 174.5 |
| 300 | 200 | 0 | 0 | 0 | 0 | 60000 | 0.049087 | 425.1 | 0 | 0 | 0 | 0 | 412 | 158.8 | 2 | 20 | 0.2 | 2 | 1.2 | 935.5 |
| 300 | 200 | 0 | 0 | 0 | 0 | 60000 | 0.049087 | 425.1 | 0 | 0 | 0 | 0 | 412 | 158.8 | 2 | 20 | 0.2 | 2 | 1.8 | 723 |
| 300 | 200 | 0 | 0 | 0 | 0 | 60000 | 0.049087 | 425.1 | 0 | 0 | 0 | 0 | 412 | 158.8 | 2 | 20 | 0.2 | 2 | 3.1 | 570.5 |
| 220 | 120 | 0 | 0 | 0 | 0 | 26400 | 0.04 | 550 | 0 | 0 | 0 | 0 | 0 | 103 | 0 | 0 | 0 | 0 | 2.5 | 107 |
| 220 | 120 | 0 | 0 | 0 | 0 | 26400 | 0.04 | 550 | 0 | 0 | 0 | 0 | 0 | 122 | 2 | 19 | 0.2 | 1.653571 | 2.5 | 184 |
| 220 | 120 | 0 | 0 | 0 | 0 | 26400 | 0.04 | 550 | 0 | 0 | 0 | 0 | 0 | 103 | 0 | 0 | 0 | 0 | 3 | 42.5 |
| 220 | 120 | 0 | 0 | 0 | 0 | 26400 | 0.04 | 550 | 0 | 0 | 0 | 0 | 0 | 122 | 2 | 19 | 0.2 | 1.653571 | 3 | 177 |
| 220 | 120 | 0 | 0 | 0 | 0 | 26400 | 0.04 | 550 | 0 | 0 | 0 | 0 | 0 | 122 | 2 | 19 | 0.2 | 1.653571 | 3 | 177.5 |
| 250 | 150 | 0 | 0 | 0 | 0 | 37500 | 0.049309 | 522.3 | 0 | 0 | 0 | 0 | 0 | 116.7 | 2 | 13 | 0.22 | 1.181818 | 2.23 | 320 |
| 250 | 150 | 0 | 0 | 0 | 0 | 37500 | 0.065745 | 522.3 | 0 | 0 | 0 | 0 | 0 | 116.7 | 2 | 13 | 0.22 | 1.181818 | 2.26 | 360 |
| 250 | 150 | 0 | 0 | 0 | 0 | 37500 | 0.065745 | 522.3 | 0 | 0 | 150 | 0.004466 | 441 | 116.7 | 2 | 13 | 0.22 | 1.181818 | 2.26 | 435 |
| 250 | 150 | 0 | 0 | 0 | 0 | 37500 | 0.065745 | 522.3 | 0 | 0 | 90 | 0.007443 | 441 | 116.7 | 2 | 13 | 0.22 | 1.181818 | 2.26 | 485 |
| 250 | 150 | 0 | 0 | 0 | 0 | 37500 | 0.065745 | 522.3 | 0 | 0 | 0 | 0 | 0 | 116.7 | 2 | 13 | 0.22 | 1.181818 | 2.26 | 325 |
| 250 | 150 | 0 | 0 | 0 | 0 | 37500 | 0.065745 | 522.3 | 0 | 0 | 0 | 0 | 0 | 116.7 | 2 | 13 | 0.22 | 1.181818 | 1.76 | 382.5 |
| 150 | 120 | 0 | 0 | 0 | 0 | 18000 | 0.080899 | 530 | 0 | 0 | 80 | 0.010467 | 570 | 116.8 | 1 | 8 | 0.12 | 0.666667 | 1.5 | 160.55 |
| 150 | 120 | 0 | 0 | 0 | 0 | 18000 | 0.080899 | 530 | 0 | 0 | 80 | 0.010467 | 570 | 115.8 | 1 | 8 | 0.12 | 0.666667 | 2.5 | 112.25 |
| 150 | 120 | 0 | 0 | 0 | 0 | 18000 | 0.080899 | 530 | 0 | 0 | 80 | 0.010467 | 570 | 112.7 | 1 | 8 | 0.12 | 0.666667 | 3.5 | 95.35 |
| 150 | 120 | 0 | 0 | 0 | 0 | 18000 | 0.080899 | 530 | 0 | 0 | 130 | 0.006441 | 570 | 115.1 | 1 | 8 | 0.12 | 0.666667 | 1.5 | 153 |
| 150 | 120 | 0 | 0 | 0 | 0 | 18000 | 0.080899 | 530 | 0 | 0 | 130 | 0.006441 | 570 | 117.6 | 1 | 8 | 0.12 | 0.666667 | 2.5 | 118.1 |
| 150 | 120 | 0 | 0 | 0 | 0 | 18000 | 0.080899 | 530 | 0 | 0 | 130 | 0.006441 | 570 | 118.9 | 1 | 8 | 0.12 | 0.666667 | 3.5 | 84.35 |
| 150 | 120 | 0 | 0 | 0 | 0 | 18000 | 0.080899 | 530 | 0 | 0 | 200 | 0.004187 | 570 | 114.3 | 1 | 8 | 0.12 | 0.666667 | 1.5 | 125.35 |
| 150 | 120 | 0 | 0 | 0 | 0 | 18000 | 0.080899 | 530 | 0 | 0 | 200 | 0.004187 | 570 | 116.5 | 1 | 8 | 0.12 | 0.666667 | 2.5 | 110.25 |
| 150 | 120 | 0 | 0 | 0 | 0 | 18000 | 0.080899 | 530 | 0 | 0 | 200 | 0.004187 | 570 | 114.8 | 1 | 8 | 0.12 | 0.666667 | 3.5 | 78 |
| 150 | 120 | 0 | 0 | 0 | 0 | 18000 | 0.080899 | 530 | 0 | 0 | 130 | 0.006441 | 570 | 118.5 | 1 | 8 | 0.12 | 0.666667 | 2.5 | 106.95 |
| 150 | 120 | 0 | 0 | 0 | 0 | 18000 | 0.080899 | 530 | 0 | 0 | 130 | 0.006441 | 570 | 118.5 | 1 | 8 | 0.12 | 0.666667 | 2.5 | 112.2 |
| 150 | 120 | 0 | 0 | 0 | 0 | 18000 | 0.080899 | 530 | 0 | 0 | 80 | 0.010467 | 570 | 125.7 | 2 | 8 | 0.12 | 1.333333 | 3.5 | 111.55 |
| 250 | 150 | 0 | 0 | 0 | 0 | 37500 | 0.065417 | 522.3 | 0 | 0 | 0 | 0 | 0 | 127 | 2 | 13 | 0.22 | 1.181818 | 1.5 | 656 |
| 250 | 150 | 0 | 0 | 0 | 0 | 37500 | 0.065417 | 522.3 | 0 | 0 | 0 | 0 | 0 | 127 | 2 | 13 | 0.22 | 1.181818 | 2.2 | 356 |
| 250 | 150 | 0 | 0 | 0 | 0 | 37500 | 0.065417 | 522.3 | 0 | 0 | 0 | 0 | 0 | 127 | 2 | 13 | 0.22 | 1.181818 | 3 | 335 |
| 250 | 150 | 0 | 0 | 0 | 0 | 37500 | 0.049309 | 522.3 | 0 | 0 | 0 | 0 | 0 | 127.1 | 2 | 13 | 0.22 | 1.181818 | 2.26 | 300.5 |
| 250 | 150 | 0 | 0 | 0 | 0 | 37500 | 0.082182 | 522.3 | 0 | 0 | 0 | 0 | 0 | 127.1 | 2 | 13 | 0.22 | 1.181818 | 2.26 | 425 |
| 250 | 150 | 0 | 0 | 0 | 0 | 37500 | 0.065745 | 522.3 | 0 | 0 | 225 | 0.001675 | 441 | 127.1 | 2 | 13 | 0.22 | 1.181818 | 2.26 | 419.25 |
| 250 | 150 | 0 | 0 | 0 | 0 | 37500 | 0.065745 | 522.3 | 0 | 0 | 150 | 0.002512 | 441 | 127.1 | 2 | 13 | 0.22 | 1.181818 | 2.26 | 430.85 |
| 250 | 150 | 0 | 0 | 0 | 0 | 37500 | 0.081771 | 548 | 0 | 0 | 0 | 0 | 472 | 160.5556 | 2 | 13 | 0.22 | 1.181818 | 2.25 | 450.5 |
| 250 | 150 | 0 | 0 | 0 | 0 | 37500 | 0.081771 | 548 | 0 | 0 | 150 | 0.002512 | 472 | 160.5556 | 2 | 13 | 0.22 | 1.181818 | 2.25 | 502.5 |
| 250 | 150 | 0 | 0 | 0 | 0 | 37500 | 0.081771 | 548 | 0 | 0 | 75 | 0.005024 | 472 | 160.5556 | 2 | 13 | 0.22 | 1.181818 | 2.25 | 529.2 |
| 250 | 150 | 0 | 0 | 0 | 0 | 37500 | 0.081771 | 548 | 0 | 0 | 300 | 0.001256 | 472 | 160.5556 | 2 | 13 | 0.22 | 1.181818 | 3 | 354.65 |
| 250 | 150 | 0 | 0 | 0 | 0 | 37500 | 0.081771 | 548 | 0 | 0 | 150 | 0.002512 | 472 | 160.5556 | 2 | 13 | 0.22 | 1.181818 | 3 | 418.3 |
| 250 | 150 | 0 | 0 | 0 | 0 | 37500 | 0.081771 | 548 | 0 | 0 | 100 | 0.003768 | 472 | 160.5556 | 3 | 13 | 0.22 | 1.772727 | 3 | 421.7 |
| 250 | 150 | 0 | 0 | 0 | 0 | 37500 | 0.065417 | 548 | 0 | 0 | 0 | 0 | 0 | 93.4 | 0 | 13 | 0.22 | 0 | 2.25 | 116.25 |
| 250 | 150 | 0 | 0 | 0 | 0 | 37500 | 0.065417 | 548 | 0 | 0 | 0 | 0 | 0 | 107.1 | 1 | 13 | 0.22 | 0.590909 | 2.25 | 249.75 |
| 250 | 150 | 0 | 0 | 0 | 0 | 37500 | 0.065417 | 548 | 0 | 0 | 0 | 0 | 0 | 131.1 | 3 | 13 | 0.22 | 1.772727 | 2.25 | 415.5 |
| 250 | 150 | 0 | 0 | 0 | 0 | 37500 | 0.065417 | 548 | 0 | 0 | 150 | 0.002512 | 472 | 160.5556 | 2 | 13 | 0.22 | 1.181818 | 1.5 | 585 |
| 250 | 150 | 0 | 0 | 0 | 0 | 37500 | 0.081771 | 548 | 0 | 0 | 150 | 0.002512 | 472 | 160.5556 | 2 | 13 | 0.22 | 1.181818 | 1.5 | 681.05 |
| 250 | 150 | 0 | 0 | 0 | 0 | 37500 | 0.098125 | 548 | 0 | 0 | 150 | 0.002512 | 472 | 139.1 | 2 | 13 | 0.22 | 1.181818 | 1.5 | 774.4 |
| 250 | 150 | 0 | 0 | 0 | 0 | 37500 | 0.098125 | 548 | 0 | 0 | 150 | 0.002512 | 472 | 127.1 | 2 | 13 | 0.22 | 1.181818 | 2.26 | 522.5 |
| 250 | 150 | 0 | 0 | 0 | 0 | 37500 | 0.065417 | 441 | 0 | 0 | 0 | 0 | 0 | 127 | 2 | 13 | 0.22 | 1.181818 | 2.26 | 325 |
| 250 | 150 | 0 | 0 | 0 | 0 | 37500 | 0.081771 | 548 | 0 | 0 | 0 | 0 | 0 | 160.5556 | 2 | 13 | 0.22 | 1.181818 | 1 | 1292.3 |
| 250 | 150 | 0 | 0 | 0 | 0 | 37500 | 0.081771 | 548 | 0 | 0 | 0 | 0 | 0 | 160.5556 | 2 | 13 | 0.22 | 1.181818 | 3.5 | 302.1 |
| 250 | 150 | 0 | 0 | 0 | 0 | 37500 | 0.065417 | 522.3 | 0 | 0 | 0 | 0 | 0 | 127 | 2 | 13 | 0.22 | 1.181818 | 1.75 | 386 |
| 300 | 150 | 0 | 0 | 0 | 0 | 45000 | 0.063388 | 543.8 | 0 | 0 | 50 | 0.007536 | 423.4 | 124.9 | 3 | 7 | 0.18 | 1.166667 | 2.33 | 564.7 |
| 300 | 150 | 0 | 0 | 0 | 0 | 45000 | 0.042092 | 543.8 | 0 | 0 | 100 | 0.003768 | 423.4 | 124.9 | 3 | 7 | 0.18 | 1.166667 | 2.35 | 463.1 |
| 300 | 150 | 0 | 0 | 0 | 0 | 45000 | 0.036075 | 543.8 | 0 | 0 | 150 | 0.002512 | 423.4 | 124.9 | 3 | 7 | 0.18 | 1.166667 | 2.21 | 436.3 |
| 300 | 150 | 0 | 0 | 0 | 0 | 45000 | 0.036075 | 543.8 | 0 | 0 | 200 | 0.001884 | 423.4 | 124.9 | 3 | 7 | 0.18 | 1.166667 | 2.21 | 364.4 |
| 300 | 150 | 0 | 0 | 0 | 0 | 45000 | 0.048494 | 543.8 | 0 | 0 | 100 | 0.003768 | 423.4 | 138.1 | 5 | 7 | 0.18 | 1.944444 | 2.32 | 517.6 |
| 300 | 150 | 0 | 0 | 0 | 0 | 45000 | 0.039949 | 543.8 | 0 | 0 | 150 | 0.002512 | 423.4 | 138.1 | 5 | 7 | 0.18 | 1.944444 | 2.29 | 480.8 |
| 300 | 150 | 0 | 0 | 0 | 0 | 45000 | 0.039949 | 543.8 | 0 | 0 | 200 | 0.001884 | 423.4 | 138.1 | 5 | 7 | 0.18 | 1.944444 | 2.29 | 406 |
| 300 | 150 | 0 | 0 | 0 | 0 | 45000 | 0.052333 | 526.3 | 0 | 0 | 200 | 0.001884 | 465.7 | 108.2 | 0 | 13 | 0.2 | 0 | 1.2 | 445 |
| 300 | 150 | 0 | 0 | 0 | 0 | 45000 | 0.052333 | 526.3 | 0 | 0 | 200 | 0.001884 | 465.7 | 118 | 1 | 13 | 0.2 | 0.65 | 1.2 | 645 |
| 300 | 150 | 0 | 0 | 0 | 0 | 45000 | 0.052333 | 526.3 | 0 | 0 | 0 | 0 | 0 | 118 | 1 | 13 | 0.2 | 0.65 | 1.2 | 580 |
| 300 | 150 | 0 | 0 | 0 | 0 | 45000 | 0.052333 | 526.3 | 0 | 0 | 100 | 0.003768 | 465.7 | 118 | 1 | 13 | 0.2 | 0.65 | 1.2 | 670 |
| 300 | 150 | 0 | 0 | 0 | 0 | 45000 | 0.052333 | 526.3 | 0 | 0 | 150 | 0.002512 | 465.7 | 118 | 1 | 13 | 0.2 | 0.65 | 1.2 | 655 |
| 300 | 150 | 0 | 0 | 0 | 0 | 45000 | 0.04643 | 526.3 | 0 | 0 | 200 | 0.001884 | 465.7 | 118 | 1 | 13 | 0.2 | 0.65 | 1.2 | 560 |
| 300 | 150 | 0 | 0 | 0 | 0 | 45000 | 0.040527 | 526.3 | 0 | 0 | 200 | 0.001884 | 465.7 | 118 | 1 | 13 | 0.2 | 0.65 | 1.2 | 490 |
| 300 | 150 | 0 | 0 | 0 | 0 | 45000 | 0.052333 | 526.3 | 0 | 0 | 200 | 0.001884 | 465.7 | 118 | 1 | 13 | 0.2 | 0.65 | 1.4 | 525 |
| 300 | 150 | 0 | 0 | 0 | 0 | 45000 | 0.052333 | 526.3 | 0 | 0 | 200 | 0.001884 | 465.7 | 118 | 1 | 13 | 0.2 | 0.65 | 1.6 | 475 |
| 250 | 150 | 0 | 0 | 0 | 0 | 37500 | 0.066245 | 451.8 | 0 | 0 | 150 | 0.004466 | 448.1 | 114.5 | 2.5 | 12 | 0.6 | 0.5 | 1.52 | 598.4 |
| 250 | 150 | 0 | 0 | 0 | 0 | 37500 | 0.066245 | 451.8 | 0 | 0 | 150 | 0.004466 | 448.1 | 114.5 | 2.5 | 12 | 0.6 | 0.5 | 1.78 | 512.6 |
| 250 | 150 | 0 | 0 | 0 | 0 | 37500 | 0.066245 | 451.8 | 0 | 0 | 150 | 0.004466 | 448.1 | 114.5 | 2.5 | 12 | 0.6 | 0.5 | 2.18 | 444.2 |
| 250 | 150 | 0 | 0 | 0 | 0 | 37500 | 0.066245 | 451.8 | 0 | 0 | 150 | 0.004466 | 448.1 | 114.5 | 2.5 | 12 | 0.6 | 0.5 | 2.54 | 395.2 |
| 250 | 150 | 0 | 0 | 0 | 0 | 37500 | 0.066245 | 451.8 | 0 | 0 | 225 | 0.001675 | 450.2 | 114.5 | 2.5 | 12 | 0.6 | 0.5 | 2.18 | 412.9 |
| 250 | 150 | 0 | 0 | 0 | 0 | 37500 | 0.066245 | 451.8 | 0 | 0 | 150 | 0.002512 | 450.2 | 114.5 | 2.5 | 12 | 0.6 | 0.5 | 2.18 | 420.9 |
| 250 | 150 | 0 | 0 | 0 | 0 | 37500 | 0.066245 | 451.8 | 0 | 0 | 90 | 0.007443 | 448.1 | 114.5 | 2.5 | 12 | 0.6 | 0.5 | 2.18 | 472.7 |
| 150 | 100 | 0 | 0 | 0 | 0 | 15000 | 0.039129 | 570 | 0 | 0 | 0 | 0 | 0 | 118 | 2 | 13 | 0.2 | 1.3 | 2.5 | 171 |
| 150 | 100 | 0 | 0 | 0 | 0 | 15000 | 0.039129 | 570 | 0 | 0 | 200 | 0.002826 | 340 | 118 | 2 | 13 | 0.2 | 1.3 | 2.5 | 175 |
| 150 | 100 | 0 | 0 | 0 | 0 | 15000 | 0.039129 | 570 | 0 | 0 | 150 | 0.003768 | 340 | 118 | 2 | 13 | 0.2 | 1.3 | 2.5 | 183 |
| 150 | 100 | 0 | 0 | 0 | 0 | 15000 | 0.039129 | 570 | 0 | 0 | 100 | 0.005652 | 340 | 118 | 2 | 13 | 0.2 | 1.3 | 2.5 | 187 |
| 150 | 100 | 0 | 0 | 0 | 0 | 15000 | 0.039129 | 570 | 0 | 0 | 0 | 0 | 0 | 110 | 1 | 13 | 0.2 | 0.65 | 2.5 | 158 |
| 150 | 100 | 0 | 0 | 0 | 0 | 15000 | 0.039129 | 570 | 0 | 0 | 0 | 0 | 0 | 100 | 0.5 | 13 | 0.2 | 0.325 | 2.5 | 142 |
| 150 | 100 | 0 | 0 | 0 | 0 | 15000 | 0.039129 | 570 | 0 | 0 | 0 | 0 | 0 | 129 | 2 | 13 | 0.2 | 1.3 | 2.5 | 178 |
| 250 | 150 | 0 | 0 | 0 | 0 | 37500 | 0.041867 | 480 | 0 | 0 | 100 | 0.006699 | 460 | 105 | 2 | 12 | 0.2 | 1.2 | 1.25 | 505 |
| 250 | 150 | 0 | 0 | 0 | 0 | 37500 | 0.041867 | 480 | 0 | 0 | 100 | 0.006699 | 460 | 105 | 2 | 12 | 0.2 | 1.2 | 1.75 | 380 |
| 350 | 200 | 0 | 0 | 0 | 0 | 70000 | 0.053915 | 481 | 0 | 0 | 200 | 0.002512 | 568 | 138.1 | 2 | 13 | 0.2 | 1.3 | 1.5 | 991.05 |
| 350 | 200 | 0 | 0 | 0 | 0 | 70000 | 0.053915 | 481 | 0 | 0 | 200 | 0.002512 | 568 | 138.1 | 2 | 13 | 0.2 | 1.3 | 2 | 778.05 |
| 350 | 200 | 0 | 0 | 0 | 0 | 70000 | 0.053915 | 481 | 0 | 0 | 200 | 0.002512 | 568 | 138.1 | 2 | 13 | 0.2 | 1.3 | 2.5 | 646.15 |
| 350 | 200 | 0 | 0 | 0 | 0 | 70000 | 0.053915 | 481 | 0 | 0 | 200 | 0.002512 | 568 | 133.3 | 1 | 13 | 0.2 | 0.65 | 2 | 687.2 |
| 350 | 200 | 0 | 0 | 0 | 0 | 70000 | 0.053915 | 481 | 0 | 0 | 200 | 0.002512 | 568 | 139.8 | 3 | 13 | 0.2 | 1.95 | 2 | 804.75 |
| 350 | 200 | 0 | 0 | 0 | 0 | 70000 | 0.053915 | 481 | 0 | 0 | 0 | 0 | 0 | 138.1 | 2 | 13 | 0.2 | 1.3 | 2 | 689.25 |
| 350 | 200 | 0 | 0 | 0 | 0 | 70000 | 0.053915 | 481 | 0 | 0 | 100 | 0.005024 | 568 | 138.1 | 2 | 13 | 0.2 | 1.3 | 2 | 866.85 |
| 350 | 200 | 0 | 0 | 0 | 0 | 70000 | 0.053915 | 481 | 0 | 0 | 300 | 0.001675 | 568 | 138.1 | 2 | 13 | 0.2 | 1.3 | 2 | 748.45 |
| 350 | 250 | 0 | 0 | 0 | 0 | 87500 | 0.071452 | 500 | 0 | 0 | 0 | 0 | 0 | 143 | 2.65 | 13 | 0.2 | 1.7225 | 2.5 | 796.4 |
| 350 | 250 | 0 | 0 | 0 | 0 | 87500 | 0.071452 | 500 | 0 | 0 | 0 | 0 | 0 | 139 | 2.65 | 13 | 0.2 | 1.7225 | 1.5 | 1068 |
| 350 | 250 | 0 | 0 | 0 | 0 | 87500 | 0.071452 | 500 | 0 | 0 | 0 | 0 | 0 | 132 | 2.65 | 13 | 0.2 | 1.7225 | 3.5 | 634.6 |
| 300 | 150 | 0 | 0 | 0 | 0 | 45000 | 0 | 0 | 0.014933 | 13.72667 | 0 | 0 | 0 | 154.2 | 1 | 13 | 0.2 | 0.65 | 1.2 | 810 |
| 300 | 150 | 0 | 0 | 0 | 0 | 45000 | 0 | 0 | 0.0112 | 9.777778 | 0 | 0 | 0 | 154.2 | 1 | 13 | 0.2 | 0.65 | 1.2 | 708.5 |
| 800 | 200 | 0 | 0 | 0 | 0 | 160000 | 0.055949 | 400 | 0 | 0 | 0 | 0 | 0 | 122.4 | 2 | 13 | 0.22 | 1.181818 | 1.22 | 2430.851 |
| 800 | 200 | 0 | 0 | 0 | 0 | 160000 | 0.055949 | 400 | 0 | 0 | 290 | 0.001732 | 400 | 122.4 | 2 | 13 | 0.22 | 1.181818 | 0.92 | 3052.979 |
| 800 | 200 | 0 | 0 | 0 | 0 | 160000 | 0.055949 | 400 | 0 | 0 | 217 | 0.002315 | 400 | 122.4 | 2 | 13 | 0.22 | 1.181818 | 1.31 | 2251 |
| 800 | 200 | 0 | 0 | 0 | 0 | 160000 | 0.055949 | 400 | 0 | 0 | 0 | 0 | 0 | 122.4 | 2 | 13 | 0.22 | 1.181818 | 1.61 | 1834.5 |
| 800 | 200 | 0 | 0 | 0 | 0 | 160000 | 0.055949 | 400 | 0 | 0 | 357 | 0.001407 | 400 | 122.4 | 2 | 13 | 0.22 | 1.181818 | 1.61 | 1982 |
| 800 | 200 | 0 | 0 | 0 | 0 | 160000 | 0.055949 | 400 | 0 | 0 | 214 | 0.002348 | 400 | 122.4 | 2 | 13 | 0.22 | 1.181818 | 1.61 | 2111 |
| 800 | 200 | 0 | 0 | 0 | 0 | 160000 | 0.055949 | 400 | 0 | 0 | 0 | 0 | 0 | 122.4 | 2 | 13 | 0.22 | 1.181818 | 1.92 | 1598 |
| 800 | 200 | 0 | 0 | 0 | 0 | 160000 | 0.055949 | 400 | 0 | 0 | 318 | 0.00158 | 400 | 122.4 | 2 | 13 | 0.22 | 1.181818 | 1.92 | 1705.5 |
| 800 | 200 | 0 | 0 | 0 | 0 | 160000 | 0.055949 | 400 | 0 | 0 | 254 | 0.001978 | 400 | 122.4 | 2 | 13 | 0.22 | 1.181818 | 1.92 | 1721.5 |
| 800 | 200 | 0 | 0 | 0 | 0 | 160000 | 0.055949 | 400 | 0 | 0 | 355 | 0.001415 | 400 | 122.4 | 2 | 13 | 0.22 | 1.181818 | 2.14 | 1468 |
| 800 | 200 | 0 | 0 | 0 | 0 | 160000 | 0.055949 | 400 | 0 | 0 | 237 | 0.00212 | 400 | 122.4 | 2 | 13 | 0.22 | 1.181818 | 2.14 | 1514.5 |
| 250 | 150 | 400 | 80 | 0 | 0 | 57500 | 0.081771 | 552 | 0 | 0 | 0 | 0 | 0 | 160.5556 | 2 | 13 | 0.22 | 1.181818 | 3.5 | 384.4 |
| 250 | 150 | 400 | 80 | 0 | 0 | 57500 | 0.081771 | 552 | 0 | 0 | 0 | 0 | 0 | 160.5556 | 2 | 13 | 0.22 | 1.181818 | 2.25 | 545.4 |
| 250 | 150 | 400 | 80 | 0 | 0 | 57500 | 0.081771 | 552 | 0 | 0 | 150 | 0.002512 | 423.4 | 160.5556 | 2 | 13 | 0.22 | 1.181818 | 2.25 | 559.8 |
| 400 | 100 | 220 | 70 | 0 | 0 | 48400 | 0.040516 | 465 | 0.013548 | 9.038678 | 0 | 0 | 0 | 142.7 | 2 | 13 | 0.2 | 1.3 | 3 | 286 |
| 400 | 100 | 220 | 70 | 0 | 0 | 48400 | 0.040516 | 465 | 0.013548 | 9.038678 | 180 | 0.005582 | 490 | 142.7 | 2 | 13 | 0.2 | 1.3 | 3 | 335 |
| 400 | 100 | 220 | 70 | 0 | 0 | 48400 | 0.040516 | 465 | 0.013548 | 9.038678 | 100 | 0.010048 | 490 | 142.7 | 2 | 13 | 0.2 | 1.3 | 3 | 380 |
| 350 | 120 | 300 | 50 | 0 | 0 | 51000 | 0.04103 | 536 | 0.012153 | 6.739765 | 500 | 0.002617 | 572 | 135 | 1.5 | 13 | 0.2 | 0.983333 | 3.13 | 408 |
| 350 | 120 | 300 | 50 | 0 | 0 | 51000 | 0.04103 | 536 | 0.012153 | 6.739765 | 500 | 0.002617 | 572 | 125 | 1.5 | 13 | 0.2 | 0.983333 | 2.49 | 499 |
| 350 | 120 | 300 | 50 | 0 | 0 | 51000 | 0.04103 | 536 | 0.012153 | 6.739765 | 500 | 0.002617 | 572 | 136 | 1.5 | 13 | 0.2 | 0.983333 | 1.03 | 1049 |
| 350 | 120 | 300 | 50 | 0 | 0 | 51000 | 0.049595 | 536 | 0.012153 | 6.739765 | 200 | 0.006542 | 572 | 136 | 1.5 | 13 | 0.2 | 0.983333 | 2.09 | 677 |
| 350 | 120 | 300 | 50 | 0 | 0 | 51000 | 0.049595 | 536 | 0.012153 | 6.739765 | 300 | 0.004361 | 572 | 143 | 1.5 | 13 | 0.2 | 0.983333 | 2.62 | 568 |
| 350 | 120 | 300 | 50 | 0 | 0 | 51000 | 0.049595 | 536 | 0.012153 | 9.190588 | 300 | 0.004361 | 572 | 141 | 1.5 | 13 | 0.2 | 0.983333 | 2.09 | 654 |
| 350 | 120 | 300 | 50 | 0 | 0 | 51000 | 0.073495 | 536 | 0 | 0 | 500 | 0.002617 | 572 | 141 | 1.5 | 13 | 0.2 | 0.983333 | 1.73 | 706 |
| 350 | 120 | 300 | 50 | 0 | 0 | 51000 | 0.073495 | 536 | 0 | 0 | 300 | 0.004361 | 572 | 122 | 1.5 | 13 | 0.2 | 0.983333 | 2.08 | 431 |
| 350 | 120 | 300 | 50 | 0 | 0 | 51000 | 0.073495 | 536 | 0 | 0 | 200 | 0.006542 | 572 | 122 | 1.5 | 13 | 0.2 | 0.983333 | 2.08 | 511 |
| 500 | 120 | 600 | 70 | 400 | 80 | 116000 | 0.018183 | 527 | 0.015201 | 5.019222 | 0 | 0 | 0 | 112.5 | 2 | 12 | 0.16 | 1.5 | 1.52 | 780.2 |
| 500 | 120 | 600 | 70 | 400 | 80 | 116000 | 0.018183 | 527 | 0.015201 | 5.269194 | 200 | 0.004187 | 451 | 110.4 | 2 | 12 | 0.16 | 1.5 | 1.52 | 936.7 |
| 500 | 120 | 600 | 70 | 400 | 80 | 116000 | 0.018183 | 527 | 0.015201 | 5.307945 | 100 | 0.008373 | 451 | 112 | 2 | 12 | 0.16 | 1.5 | 1.52 | 1063 |
| 500 | 120 | 600 | 70 | 400 | 80 | 116000 | 0.018183 | 527 | 0.015201 | 5.054218 | 0 | 0 | 0 | 111.9 | 2 | 12 | 0.16 | 1.5 | 2.28 | 621.5 |
| 500 | 120 | 600 | 70 | 400 | 80 | 116000 | 0.018183 | 527 | 0.015201 | 5.539784 | 200 | 0.004187 | 451 | 109.1 | 2 | 12 | 0.16 | 1.5 | 2.28 | 745.9 |
| 500 | 120 | 600 | 70 | 400 | 80 | 116000 | 0.018183 | 527 | 0.015201 | 4.964237 | 100 | 0.008373 | 451 | 111.3 | 2 | 12 | 0.16 | 1.5 | 2.28 | 876.7 |
| 500 | 120 | 600 | 70 | 400 | 80 | 116000 | 0.018183 | 527 | 0.015201 | 5.415428 | 0 | 0 | 0 | 107.9 | 2 | 12 | 0.16 | 1.5 | 3.04 | 493 |
| 500 | 120 | 600 | 70 | 400 | 80 | 116000 | 0.018183 | 527 | 0.015201 | 5.347926 | 200 | 0.004187 | 451 | 110.4 | 2 | 12 | 0.16 | 1.5 | 3.04 | 605 |
| 400 | 70 | 300 | 40 | 200 | 105 | 50850 | 0.040051 | 555.9 | 0.011429 | 4.719764 | 0 | 0 | 0 | 121.2 | 0.75 | 13 | 0.2 | 0.4875 | 1.28 | 573.5 |
| 400 | 70 | 300 | 40 | 200 | 105 | 50850 | 0.040051 | 556.9 | 0.011429 | 4.719764 | 0 | 0 | 0 | 125.1 | 0.75 | 13 | 0.2 | 0.4875 | 1.71 | 345.1 |
| 400 | 70 | 300 | 40 | 200 | 105 | 50850 | 0.040051 | 557.9 | 0.011429 | 4.719764 | 0 | 0 | 0 | 117.8 | 0.75 | 13 | 0.2 | 0.4875 | 2 | 253.5 |
| 400 | 70 | 300 | 40 | 200 | 105 | 50850 | 0.040051 | 558.9 | 0.011429 | 4.719764 | 0 | 0 | 0 | 121.2 | 0.75 | 13 | 0.2 | 0.4875 | 2.57 | 202.4 |
| 400 | 70 | 300 | 40 | 200 | 105 | 50850 | 0.060077 | 558.9 | 0 | 0 | 0 | 0 | 0 | 119.2 | 0.75 | 13 | 0.2 | 0.4875 | 2 | 224.1 |
| 400 | 70 | 300 | 40 | 200 | 105 | 50850 | 0.040051 | 558.9 | 0.011429 | 2.949853 | 0 | 0 | 0 | 111.3 | 0.75 | 13 | 0.2 | 0.4875 | 2 | 236.9 |
| 400 | 70 | 300 | 40 | 200 | 105 | 50850 | 0.040051 | 558.9 | 0.011429 | 6.686332 | 0 | 0 | 0 | 121.2 | 0.75 | 13 | 0.2 | 0.4875 | 2 | 316.4 |
| 300 | 150 | 550 | 50 | 370 | 50 | 76000 | 0 | 0 | 0.014933 | 8.034211 | 0 | 0 | 0 | 154.2 | 1 | 13 | 0.2 | 0.65 | 1.2 | 955.5 |
| 400 | 100 | 575 | 50 | 225 | 50 | 70000 | 0 | 0 | 0.016 | 8.838571 | 0 | 0 | 0 | 154.2 | 1 | 13 | 0.2 | 0.65 | 1.2 | 865.5 |
| 140 | 40 | 120 | 35 | 0 | 0 | 8400 | 0.049579 | 760.9 | 0 | 0 | 0 | 0 | 0 | 116 | 2 | 13 | 0.2 | 1.3 | 3.17 | 76 |
| 140 | 40 | 120 | 35 | 0 | 0 | 8400 | 0.049579 | 760.9 | 0 | 0 | 0 | 0 | 0 | 96.3 | 0.5 | 13 | 0.2 | 0.325 | 3.17 | 52.1 |
| 140 | 40 | 120 | 35 | 0 | 0 | 8400 | 0.049579 | 760.9 | 0 | 0 | 0 | 0 | 0 | 94.3 | 1 | 13 | 0.2 | 0.65 | 3.17 | 52.5 |
| 140 | 40 | 120 | 35 | 0 | 0 | 8400 | 0.049579 | 760.9 | 0 | 0 | 0 | 0 | 0 | 116 | 2 | 13 | 0.2 | 1.3 | 2.5 | 104 |
| 140 | 40 | 120 | 35 | 0 | 0 | 8400 | 0.049579 | 760.9 | 0 | 0 | 0 | 0 | 0 | 116 | 2 | 13 | 0.2 | 1.3 | 3.75 | 88 |
| 140 | 40 | 120 | 35 | 0 | 0 | 8400 | 0.049579 | 760.9 | 0 | 0 | 190 | 0.007437 | 417.2 | 116 | 2 | 13 | 0.2 | 1.3 | 3.17 | 97 |
| 140 | 40 | 120 | 35 | 0 | 0 | 8400 | 0.049579 | 760.9 | 0 | 0 | 127 | 0.011126 | 417.2 | 116 | 2 | 13 | 0.2 | 1.3 | 3.17 | 109 |
| 140 | 40 | 120 | 35 | 0 | 0 | 8400 | 0.049579 | 760.9 | 0 | 0 | 0 | 0 | 0 | 120.5 | 2 | 6 | 0.2 | 0.6 | 3.17 | 68.9 |
| 140 | 40 | 120 | 35 | 0 | 0 | 8400 | 0.049579 | 760.9 | 0 | 0 | 0 | 0 | 0 | 113.2 | 2 | 30 | 0.6 | 1 | 3.17 | 66.2 |
| 140 | 40 | 120 | 35 | 0 | 0 | 8400 | 0.049579 | 760.9 | 0 | 0 | 0 | 0 | 0 | 120.5 | 2 | 13 | 0.2 | 1.3 | 3.17 | 47.1 |
| 140 | 40 | 120 | 35 | 0 | 0 | 8400 | 0.049579 | 760.9 | 0 | 0 | 0 | 0 | 0 | 113.2 | 2 | 13 | 0.2 | 1.3 | 3.17 | 77 |
| 600 | 70 | 600 | 65 | 300 | 110 | 101750 | 0.022825 | 350 | 0.028269 | 8.101425 | 100 | 0.016149 | 350 | 140 | 2 | 13 | 0.2 | 1.3 | 1.06 | 1600 |
| 600 | 70 | 600 | 65 | 300 | 110 | 101750 | 0.022825 | 350 | 0.028269 | 8.101425 | 100 | 0.016149 | 350 | 140 | 2 | 13 | 0.2 | 1.3 | 2.12 | 965.7 |
| 600 | 70 | 600 | 65 | 300 | 110 | 101750 | 0.022825 | 350 | 0.028269 | 8.101425 | 100 | 0.016149 | 350 | 140 | 2 | 13 | 0.2 | 1.3 | 3.19 | 926.7 |
| 160 | 40 | 120 | 20 | 80 | 47.5 | 9900 | 0.094351 | 542.1 | 0 | 0 | 0 | 0 | 0 | 125 | 0.75 | 13 | 0.2 | 0.4875 | 2.5 | 42.9 |
| 225 | 55 | 170 | 25 | 115 | 70 | 19450 | 0.091025 | 542.1 | 0 | 0 | 0 | 0 | 0 | 125 | 0.75 | 13 | 0.2 | 0.4875 | 2.5 | 82.7 |
| 275 | 70 | 210 | 35 | 140 | 82.5 | 29925 | 0.085475 | 542.1 | 0 | 0 | 0 | 0 | 0 | 125 | 0.75 | 13 | 0.2 | 0.4875 | 2.5 | 176.6 |
| 320 | 80 | 240 | 40 | 160 | 95 | 39600 | 0.092223 | 542.1 | 0 | 0 | 0 | 0 | 0 | 125 | 0.75 | 13 | 0.2 | 0.4875 | 2.5 | 183.5 |
| 355 | 90 | 270 | 45 | 180 | 105 | 49500 | 0.087926 | 542.1 | 0 | 0 | 0 | 0 | 0 | 125 | 0.75 | 13 | 0.2 | 0.4875 | 2.5 | 241.6 |
| 500 | 50 | 360 | 60 | 200 | 60 | 52600 | 0.039818 | 451 | 0.017872 | 7.060837 | 200 | 0.005652 | 451 | 175 | 2.5 | 13 | 0.2 | 1.625 | 1.68 | 576.45 |
| 500 | 50 | 360 | 60 | 200 | 60 | 52600 | 0.039818 | 451 | 0.017872 | 8.026616 | 200 | 0.005652 | 451 | 175 | 2.5 | 13 | 0.2 | 1.625 | 2.42 | 460 |
| 500 | 50 | 360 | 60 | 200 | 60 | 52600 | 0.039818 | 451 | 0.017872 | 7.703422 | 0 | 0 | 0 | 175 | 2.5 | 13 | 0.2 | 1.625 | 3.17 | 265 |
| 140 | 100 | 0 | 0 | 0 | 0 | 14000 | 0.035886 | 520 | 0 | 0 | 0 | 0 | 0 | 78 | 0 | 0 | 0 | 0 | 3.5 | 35.5 |
| 140 | 100 | 0 | 0 | 0 | 0 | 14000 | 0.035886 | 520 | 0 | 0 | 0 | 0 | 0 | 94 | 0.5 | 13 | 0.2 | 0.325 | 3.5 | 66.5 |
| 140 | 100 | 0 | 0 | 0 | 0 | 14000 | 0.035886 | 520 | 0 | 0 | 0 | 0 | 0 | 98 | 1 | 13 | 0.2 | 0.65 | 3.5 | 70 |
| 140 | 100 | 0 | 0 | 0 | 0 | 14000 | 0.035886 | 520 | 0 | 0 | 0 | 0 | 0 | 103 | 1.5 | 13 | 0.2 | 0.975 | 3.5 | 77.5 |
| 140 | 100 | 0 | 0 | 0 | 0 | 14000 | 0.035886 | 520 | 0 | 0 | 0 | 0 | 0 | 110 | 2 | 13 | 0.2 | 1.3 | 3.5 | 82.5 |
| 140 | 100 | 0 | 0 | 0 | 0 | 14000 | 0.049904 | 658 | 0 | 0 | 0 | 0 | 0 | 110 | 2 | 13 | 0.2 | 1.3 | 3.5 | 107.5 |
| 140 | 100 | 0 | 0 | 0 | 0 | 14000 | 0.056071 | 658 | 0 | 0 | 0 | 0 | 0 | 110 | 2 | 13 | 0.2 | 1.3 | 3.5 | 112.5 |
| 140 | 100 | 0 | 0 | 0 | 0 | 14000 | 0.035886 | 520 | 0 | 0 | 0 | 0 | 0 | 101 | 2 | 13 | 0.2 | 1.3 | 3.5 | 77.5 |
| 140 | 100 | 0 | 0 | 0 | 0 | 14000 | 0.035886 | 520 | 0 | 0 | 0 | 0 | 0 | 93.4 | 2 | 13 | 0.2 | 1.3 | 3.5 | 75 |
| 140 | 100 | 0 | 0 | 0 | 0 | 14000 | 0.035886 | 520 | 0 | 0 | 0 | 0 | 0 | 110 | 2 | 13 | 0.2 | 1.3 | 2.5 | 125 |
| 140 | 100 | 0 | 0 | 0 | 0 | 14000 | 0.035886 | 520 | 0 | 0 | 0 | 0 | 0 | 110 | 2 | 13 | 0.2 | 1.3 | 3 | 97.5 |
| 140 | 100 | 0 | 0 | 0 | 0 | 14000 | 0.035886 | 520 | 0 | 0 | 0 | 0 | 0 | 110 | 2 | 13 | 0.2 | 1.3 | 4 | 62.5 |
| 140 | 100 | 0 | 0 | 0 | 0 | 14000 | 0.035886 | 520 | 0 | 0 | 0 | 0 | 0 | 110 | 2 | 13 | 0.2 | 1.3 | 4.5 | 59.5 |
| 140 | 100 | 0 | 0 | 0 | 0 | 14000 | 0.035886 | 520 | 0 | 0 | 0 | 0 | 0 | 98 | 1 | 13 | 0.2 | 0.65 | 2.5 | 100 |
| 140 | 100 | 0 | 0 | 0 | 0 | 14000 | 0.035886 | 520 | 0 | 0 | 0 | 0 | 0 | 98 | 1 | 13 | 0.2 | 0.65 | 4.5 | 55 |
| 140 | 100 | 0 | 0 | 0 | 0 | 14000 | 0.035568 | 520 | 0 | 0 | 0 | 0 | 0 | 125 | 2 | 13 | 0.2 | 1.3 | 3.5 | 94 |
| 140 | 100 | 0 | 0 | 0 | 0 | 14000 | 0.035256 | 520 | 0 | 0 | 0 | 0 | 0 | 142 | 2 | 13 | 0.2 | 1.3 | 3.5 | 101 |
| 140 | 100 | 0 | 0 | 0 | 0 | 14000 | 0.03495 | 520 | 0 | 0 | 0 | 0 | 0 | 151 | 2 | 13 | 0.2 | 1.3 | 3.5 | 110 |
| 180 | 120 | 0 | 0 | 0 | 0 | 21600 | 0.039222 | 494 | 0 | 0 | 0 | 0 | 0 | 134.5 | 0.4 | 40 | 0.2 | 0.8 | 1 | 475.65 |
| 180 | 120 | 0 | 0 | 0 | 0 | 21600 | 0.039222 | 494 | 0 | 0 | 0 | 0 | 0 | 134.5 | 0.4 | 40 | 0.2 | 0.8 | 1.5 | 416.8 |
| 180 | 120 | 0 | 0 | 0 | 0 | 21600 | 0.039222 | 494 | 0 | 0 | 0 | 0 | 0 | 134.5 | 0.4 | 40 | 0.2 | 0.8 | 2 | 103.95 |
| 240 | 120 | 0 | 0 | 0 | 0 | 28800 | 0.038889 | 494 | 0 | 0 | 0 | 0 | 0 | 134.5 | 0.4 | 40 | 0.2 | 0.8 | 1 | 400.6 |
| 240 | 120 | 0 | 0 | 0 | 0 | 28800 | 0.038889 | 494 | 0 | 0 | 0 | 0 | 0 | 134.5 | 0.4 | 40 | 0.2 | 0.8 | 1.5 | 340.8 |
| 240 | 120 | 0 | 0 | 0 | 0 | 28800 | 0.038889 | 494 | 0 | 0 | 0 | 0 | 0 | 134.5 | 0.4 | 40 | 0.2 | 0.8 | 2 | 132.4 |
| 300 | 120 | 0 | 0 | 0 | 0 | 36000 | 0.039938 | 494 | 0 | 0 | 0 | 0 | 0 | 134.5 | 0.4 | 40 | 0.2 | 0.8 | 1 | 475.65 |
| 300 | 120 | 0 | 0 | 0 | 0 | 36000 | 0.039938 | 494 | 0 | 0 | 0 | 0 | 0 | 134.5 | 0.4 | 40 | 0.2 | 0.8 | 1.5 | 308.45 |
| 300 | 120 | 0 | 0 | 0 | 0 | 36000 | 0.039938 | 494 | 0 | 0 | 0 | 0 | 0 | 134.5 | 0.4 | 40 | 0.2 | 0.8 | 2 | 328.05 |
| 290 | 150 | 0 | 0 | 0 | 0 | 43500 | 0.078244 | 617.7 | 0 | 0 | 0 | 0 | 0 | 166.9 | 1.5 | 17.5 | 0.2 | 1.3125 | 3 | 475.8 |
| 290 | 150 | 0 | 0 | 0 | 0 | 43500 | 0.078244 | 617.7 | 0 | 0 | 165 | 0.006343 | 537.5 | 166.9 | 1.5 | 17.5 | 0.2 | 1.3125 | 3 | 537.3 |
| 225 | 150 | 0 | 0 | 0 | 0 | 33750 | 0.019356 | 494 | 0 | 0 | 200 | 0.005233 | 430 | 138 | 1 | 13 | 0.22 | 0.590909 | 1.8 | 172.5 |
| 225 | 150 | 0 | 0 | 0 | 0 | 33750 | 0.019356 | 494 | 0 | 0 | 200 | 0.005233 | 430 | 150 | 2 | 13 | 0.22 | 1.181818 | 1.8 | 185.5 |
| 225 | 150 | 0 | 0 | 0 | 0 | 33750 | 0.019356 | 494 | 0 | 0 | 370 | 0.002829 | 430 | 138 | 1 | 13 | 0.22 | 0.590909 | 1.8 | 147.5 |
| 225 | 150 | 0 | 0 | 0 | 0 | 33750 | 0.019356 | 494 | 0 | 0 | 370 | 0.002829 | 430 | 150 | 2 | 13 | 0.22 | 1.181818 | 1.8 | 176 |
| 225 | 150 | 0 | 0 | 0 | 0 | 33750 | 0.019356 | 494 | 0 | 0 | 370 | 0.002829 | 430 | 138 | 1 | 13 | 0.22 | 0.590909 | 1.8 | 155.5 |
| 225 | 150 | 0 | 0 | 0 | 0 | 33750 | 0.019356 | 494 | 0 | 0 | 370 | 0.002829 | 430 | 150 | 2 | 13 | 0.22 | 1.181818 | 1.8 | 183 |
| 225 | 150 | 0 | 0 | 0 | 0 | 33750 | 0.019356 | 494 | 0 | 0 | 370 | 0.002829 | 430 | 138 | 1 | 13 | 0.22 | 0.590909 | 2.6 | 104 |
| 225 | 150 | 0 | 0 | 0 | 0 | 33750 | 0.019356 | 494 | 0 | 0 | 370 | 0.002829 | 430 | 150 | 2 | 13 | 0.22 | 1.181818 | 2.6 | 114.5 |
| 225 | 150 | 0 | 0 | 0 | 0 | 33750 | 0.019356 | 494 | 0 | 0 | 370 | 0.002829 | 430 | 138 | 1 | 13 | 0.22 | 0.590909 | 2.6 | 116 |
| 225 | 150 | 0 | 0 | 0 | 0 | 33750 | 0.019356 | 494 | 0 | 0 | 370 | 0.002829 | 430 | 150 | 2 | 13 | 0.22 | 1.181818 | 2.6 | 125 |
| 152 | 152 | 0 | 0 | 0 | 0 | 23104 | 0.077467 | 690 | 0 | 0 | 0 | 0 | 0 | 137 | 2 | 13 | 0.18 | 1.444444 | 1.2 | 446 |
| 152 | 152 | 0 | 0 | 0 | 0 | 23104 | 0.059514 | 690 | 0 | 0 | 0 | 0 | 0 | 137 | 2 | 13 | 0.18 | 1.444444 | 1.2 | 452 |
| 152 | 152 | 0 | 0 | 0 | 0 | 23104 | 0.077467 | 400 | 0 | 0 | 0 | 0 | 0 | 137 | 2 | 13 | 0.18 | 1.444444 | 1.2 | 409.5 |
| 152 | 152 | 0 | 0 | 0 | 0 | 23104 | 0.059514 | 400 | 0 | 0 | 0 | 0 | 0 | 137 | 2 | 13 | 0.18 | 1.444444 | 1.2 | 377.5 |
| 152 | 152 | 0 | 0 | 0 | 0 | 23104 | 0.043696 | 400 | 0 | 0 | 0 | 0 | 0 | 137 | 2 | 13 | 0.18 | 1.444444 | 1.2 | 392.5 |
| 152 | 152 | 0 | 0 | 0 | 0 | 23104 | 0.048798 | 400 | 0 | 0 | 0 | 0 | 0 | 125 | 2 | 13 | 0.18 | 1.444444 | 1.2 | 349 |
| 152 | 152 | 0 | 0 | 0 | 0 | 23104 | 0.039218 | 400 | 0 | 0 | 0 | 0 | 0 | 125 | 2 | 13 | 0.18 | 1.444444 | 1.2 | 332 |
| 152 | 152 | 0 | 0 | 0 | 0 | 23104 | 0.048798 | 400 | 0 | 0 | 0 | 0 | 0 | 125 | 2 | 13 | 0.18 | 1.444444 | 1.2 | 335 |
| 152 | 152 | 0 | 0 | 0 | 0 | 23104 | 0.039218 | 400 | 0 | 0 | 0 | 0 | 0 | 125 | 2 | 13 | 0.18 | 1.444444 | 1.2 | 336 |
| 203 | 102 | 0 | 0 | 0 | 0 | 20706 | 0.034589 | 400 | 0 | 0 | 0 | 0 | 0 | 125 | 2 | 13 | 0.18 | 1.444444 | 0.9 | 409 |
| 203 | 102 | 0 | 0 | 0 | 0 | 20706 | 0.021891 | 400 | 0 | 0 | 0 | 0 | 0 | 125 | 2 | 13 | 0.18 | 1.444444 | 0.9 | 342 |
| 76 | 152 | 0 | 0 | 0 | 0 | 11552 | 0.056235 | 400 | 0 | 0 | 0 | 0 | 0 | 125 | 2 | 13 | 0.18 | 1.444444 | 2.8 | 106 |
| 76 | 152 | 0 | 0 | 0 | 0 | 11552 | 0.02817 | 400 | 0 | 0 | 0 | 0 | 0 | 125 | 2 | 13 | 0.18 | 1.444444 | 2.8 | 85 |
| 76 | 152 | 0 | 0 | 0 | 0 | 11552 | 0.027667 | 400 | 0 | 0 | 0 | 0 | 0 | 125 | 2 | 13 | 0.18 | 1.444444 | 2.8 | 71 |
| 400 | 80 | 0 | 0 | 0 | 0 | 32000 | 0.036334 | 491.2 | 0 | 0 | 150 | 0.00471 | 491.2 | 188.1 | 1.5 | 25 | 1 | 0.375 | 0.79 | 445 |
| 400 | 80 | 0 | 0 | 0 | 0 | 32000 | 0.036231 | 491.2 | 0 | 0 | 150 | 0.008373 | 491.2 | 188.1 | 1.5 | 25 | 1 | 0.375 | 0.79 | 530 |
| 400 | 80 | 0 | 0 | 0 | 0 | 32000 | 0.036128 | 491.2 | 0 | 0 | 150 | 0.00471 | 491.2 | 188.1 | 1.5 | 25 | 1 | 0.375 | 0.94 | 415 |
| 400 | 80 | 0 | 0 | 0 | 0 | 32000 | 0.036025 | 491.2 | 0 | 0 | 150 | 0.008373 | 491.2 | 188.1 | 1.5 | 25 | 1 | 0.375 | 0.94 | 455 |
| 400 | 80 | 0 | 0 | 0 | 0 | 32000 | 0.035924 | 491.2 | 0 | 0 | 75 | 0.016747 | 491.2 | 188.1 | 1.5 | 25 | 1 | 0.375 | 0.94 | 505 |
| 150 | 100 | 0 | 0 | 0 | 0 | 15000 | 0.050645 | 478 | 0 | 0 | 0 | 0 | 0 | 126 | 0 | 0 | 0 | 0 | 4.03 | 35.6 |
| 150 | 100 | 0 | 0 | 0 | 0 | 15000 | 0.050645 | 478 | 0 | 0 | 0 | 0 | 0 | 127 | 0.5 | 6 | 0.16 | 0.1875 | 4.03 | 40.65 |
| 150 | 100 | 0 | 0 | 0 | 0 | 15000 | 0.050645 | 478 | 0 | 0 | 0 | 0 | 0 | 131 | 1 | 6 | 0.16 | 0.375 | 4.03 | 59.08 |
| 150 | 100 | 0 | 0 | 0 | 0 | 15000 | 0.050645 | 478 | 0 | 0 | 0 | 0 | 0 | 140 | 1.5 | 6 | 0.16 | 0.5625 | 4.03 | 55.58 |
| 150 | 100 | 0 | 0 | 0 | 0 | 15000 | 0.050645 | 478 | 0 | 0 | 0 | 0 | 0 | 132 | 0.5 | 13 | 0.16 | 0.40625 | 4.03 | 56.19 |
| 150 | 100 | 0 | 0 | 0 | 0 | 15000 | 0.050645 | 478 | 0 | 0 | 0 | 0 | 0 | 137 | 1 | 13 | 0.16 | 0.8125 | 4.03 | 63.49 |
| 150 | 100 | 0 | 0 | 0 | 0 | 15000 | 0.050645 | 478 | 0 | 0 | 0 | 0 | 0 | 130 | 0.5 | 30 | 0.55 | 0.272727 | 4.03 | 40.58 |
| 150 | 100 | 0 | 0 | 0 | 0 | 15000 | 0.050645 | 478 | 0 | 0 | 0 | 0 | 0 | 131 | 1 | 30 | 0.55 | 0.545455 | 4.03 | 61.5 |
| 150 | 100 | 0 | 0 | 0 | 0 | 15000 | 0.050645 | 478 | 0 | 0 | 0 | 0 | 0 | 139 | 1.5 | 30 | 0.55 | 0.818182 | 4.03 | 71.74 |
| 150 | 100 | 0 | 0 | 0 | 0 | 15000 | 0.050645 | 478 | 0 | 0 | 0 | 0 | 0 | 127 | 0.5 | 60 | 0.75 | 0.4 | 4.03 | 36.21 |
| 150 | 100 | 0 | 0 | 0 | 0 | 15000 | 0.050645 | 478 | 0 | 0 | 0 | 0 | 0 | 129 | 1 | 60 | 0.75 | 0.8 | 4.03 | 48.58 |
| 150 | 100 | 0 | 0 | 0 | 0 | 15000 | 0.050645 | 478 | 0 | 0 | 0 | 0 | 0 | 136 | 1.5 | 60 | 0.75 | 1.2 | 4.03 | 57.89 |
| 150 | 100 | 0 | 0 | 0 | 0 | 15000 | 0.050645 | 478 | 0 | 0 | 0 | 0 | 0 | 127 | 0.5 | 60 | 0.9 | 0.333333 | 4.03 | 41.55 |
| 150 | 100 | 0 | 0 | 0 | 0 | 15000 | 0.050645 | 478 | 0 | 0 | 0 | 0 | 0 | 130 | 1 | 60 | 0.9 | 0.666667 | 4.03 | 63.76 |
| 150 | 100 | 0 | 0 | 0 | 0 | 15000 | 0.050645 | 478 | 0 | 0 | 0 | 0 | 0 | 138 | 1.5 | 60 | 0.9 | 1 | 4.03 | 62.49 |
| 350 | 165 | 0 | 0 | 0 | 0 | 57750 | 0.07495 | 474 | 0 | 0 | 0 | 0 | 0 | 118.6 | 0 | 0 | 0 | 0 | 1.5 | 364.4133 |
| 350 | 165 | 0 | 0 | 0 | 0 | 57750 | 0.07495 | 474 | 0 | 0 | 0 | 0 | 0 | 115.3 | 0.75 | 30 | 0.38 | 0.592105 | 1.5 | 428.4054 |
| 350 | 165 | 0 | 0 | 0 | 0 | 57750 | 0.07495 | 474 | 0 | 0 | 0 | 0 | 0 | 124.1 | 1.5 | 30 | 0.38 | 1.184211 | 1.5 | 673.8485 |
| 350 | 165 | 0 | 0 | 0 | 0 | 57750 | 0.07495 | 474 | 0 | 0 | 0 | 0 | 0 | 111.7 | 0.75 | 8 | 0.038 | 1.578947 | 1.5 | 439.8004 |
| 350 | 165 | 0 | 0 | 0 | 0 | 57750 | 0.07495 | 474 | 0 | 0 | 0 | 0 | 0 | 104.5 | 2.25 | 8 | 0.038 | 4.736842 | 1.5 | 495.5573 |
| 350 | 165 | 0 | 0 | 0 | 0 | 57750 | 0.07495 | 474 | 0 | 0 | 0 | 0 | 0 | 126.3 | 0.75 | 60 | 0.9 | 0.5 | 1.5 | 515.8729 |
| 350 | 165 | 0 | 0 | 0 | 0 | 57750 | 0.07495 | 474 | 0 | 0 | 0 | 0 | 0 | 107.4 | 1.5 | 60 | 0.9 | 1 | 1.5 | 560.1831 |
| 350 | 165 | 0 | 0 | 0 | 0 | 57750 | 0.07495 | 474 | 0 | 0 | 0 | 0 | 0 | 124 | 2.25 | 60 | 0.9 | 1.5 | 1.5 | 692.6856 |
| 350 | 165 | 0 | 0 | 0 | 0 | 57750 | 0.07495 | 474 | 0 | 0 | 0 | 0 | 0 | 122.9 | 1.5 | 30 | 0.38 | 1.092105 | 1.5 | 703.874 |
| 350 | 165 | 0 | 0 | 0 | 0 | 57750 | 0.07495 | 474 | 0 | 0 | 0 | 0 | 0 | 118.7 | 2.25 | 30 | 0.38 | 1.684211 | 1.5 | 813.2647 |
| 350 | 165 | 0 | 0 | 0 | 0 | 57750 | 0.07495 | 474 | 0 | 0 | 0 | 0 | 0 | 114.4 | 2.25 | 30 | 0.38 | 1.592105 | 1.5 | 743.3363 |
| 350 | 165 | 0 | 0 | 0 | 0 | 57750 | 0.07495 | 474 | 0 | 0 | 0 | 0 | 0 | 97.6 | 2.25 | 30 | 0.38 | 2.504386 | 1.5 | 623.0165 |
| 350 | 165 | 0 | 0 | 0 | 0 | 57750 | 0.07495 | 474 | 0 | 0 | 0 | 0 | 0 | 94.5 | 0 | 0 | 0 | 0 | 3.3 | 104.2589 |
| 350 | 165 | 0 | 0 | 0 | 0 | 57750 | 0.07495 | 474 | 0 | 0 | 0 | 0 | 0 | 116.5 | 0.75 | 30 | 0.38 | 0.592105 | 3.3 | 287.086 |
| 350 | 165 | 0 | 0 | 0 | 0 | 57750 | 0.07495 | 474 | 0 | 0 | 0 | 0 | 0 | 102.9 | 1.5 | 30 | 0.38 | 1.184211 | 3.3 | 330.7338 |
| 350 | 165 | 0 | 0 | 0 | 0 | 57750 | 0.07495 | 474 | 0 | 0 | 0 | 0 | 0 | 115 | 0.75 | 8 | 0.038 | 1.578947 | 3.3 | 170.219 |
| 350 | 165 | 0 | 0 | 0 | 0 | 57750 | 0.07495 | 474 | 0 | 0 | 0 | 0 | 0 | 94.2 | 2.25 | 8 | 0.038 | 4.736842 | 3.3 | 258.1514 |
| 350 | 165 | 0 | 0 | 0 | 0 | 57750 | 0.07495 | 474 | 0 | 0 | 0 | 0 | 0 | 133.7 | 0.75 | 60 | 0.9 | 0.5 | 3.3 | 193.4584 |
| 350 | 165 | 0 | 0 | 0 | 0 | 57750 | 0.07495 | 474 | 0 | 0 | 0 | 0 | 0 | 132 | 1.5 | 60 | 0.9 | 1 | 3.3 | 325.3031 |
| 350 | 165 | 0 | 0 | 0 | 0 | 57750 | 0.07495 | 474 | 0 | 0 | 0 | 0 | 0 | 116.6 | 2.25 | 60 | 0.9 | 1.5 | 3.3 | 347.4304 |
| 350 | 165 | 0 | 0 | 0 | 0 | 57750 | 0.07495 | 474 | 0 | 0 | 0 | 0 | 0 | 136 | 1.5 | 30 | 0.38 | 1.092105 | 3.3 | 370.2188 |
| 350 | 165 | 0 | 0 | 0 | 0 | 57750 | 0.07495 | 474 | 0 | 0 | 0 | 0 | 0 | 109.7 | 2.25 | 30 | 0.38 | 1.684211 | 3.3 | 377.433 |
| 350 | 165 | 0 | 0 | 0 | 0 | 57750 | 0.07495 | 474 | 0 | 0 | 0 | 0 | 0 | 124.2 | 2.25 | 30 | 0.38 | 1.592105 | 3.3 | 411.1654 |
| 350 | 165 | 0 | 0 | 0 | 0 | 57750 | 0.07495 | 474 | 0 | 0 | 0 | 0 | 0 | 113 | 2.25 | 30 | 0.38 | 2.504386 | 3.3 | 328.3439 |
| 350 | 200 | 0 | 0 | 0 | 0 | 70000 | 0.043806 | 445 | 0 | 0 | 0 | 0 | 0 | 198 | 0 | 0 | 0 | 0 | 3.4 | 119.8 |
| 350 | 200 | 0 | 0 | 0 | 0 | 70000 | 0.043806 | 445 | 0 | 0 | 150 | 0.005233 | 422 | 198 | 0 | 0 | 0 | 0 | 3.4 | 259.4 |
| 350 | 200 | 0 | 0 | 0 | 0 | 70000 | 0.043806 | 445 | 0 | 0 | 0 | 0 | 0 | 117 | 2 | 13 | 0.2 | 1.3 | 3.4 | 258.9 |
| 350 | 200 | 0 | 0 | 0 | 0 | 70000 | 0.043806 | 445 | 0 | 0 | 0 | 0 | 0 | 198 | 0 | 0 | 0 | 0 | 2 | 235.4 |
| 350 | 200 | 0 | 0 | 0 | 0 | 70000 | 0.043806 | 445 | 0 | 0 | 0 | 0 | 0 | 217 | 2 | 13 | 0.2 | 1.3 | 2 | 568.4 |
| 350 | 200 | 0 | 0 | 0 | 0 | 70000 | 0.043806 | 445 | 0 | 0 | 0 | 0 | 0 | 117 | 2 | 13 | 0.2 | 1.3 | 2 | 493.2 |
| 267 | 112 | 0 | 0 | 0 | 0 | 29904 | 0 | 0 | 0.023838 | 0 | 0 | 0 | 0 | 144.1 | 0 | 0 | 0 | 0 | 3.5 | 57.2 |
| 267 | 112 | 0 | 0 | 0 | 0 | 29904 | 0 | 0 | 0.023838 | 0 | 0 | 0 | 0 | 144.1 | 0 | 0 | 0 | 0 | 3.5 | 54.9 |
| 266 | 114 | 0 | 0 | 0 | 0 | 30324 | 0 | 0 | 0.023646 | 0 | 0 | 0 | 0 | 169.2 | 0 | 0 | 0 | 0 | 3.5 | 62.5 |
| 266 | 114 | 0 | 0 | 0 | 0 | 30324 | 0 | 0 | 0.023646 | 0 | 0 | 0 | 0 | 169.2 | 0 | 0 | 0 | 0 | 4 | 63.7 |
| 269 | 113 | 0 | 0 | 0 | 0 | 30397 | 0 | 0 | 0.023293 | 9.7498 | 0 | 0 | 0 | 168.1 | 0 | 0 | 0 | 0 | 4 | 159.1 |
| 269 | 113 | 0 | 0 | 0 | 0 | 30397 | 0 | 0 | 0.023293 | 9.7498 | 0 | 0 | 0 | 168.1 | 0 | 0 | 0 | 0 | 5 | 113.7 |
| 269 | 113 | 0 | 0 | 0 | 0 | 30397 | 0 | 0 | 0.023293 | 21.0125 | 0 | 0 | 0 | 168.1 | 0 | 0 | 0 | 0 | 5 | 151.5 |
| 269 | 113 | 0 | 0 | 0 | 0 | 30397 | 0 | 0 | 0.023293 | 21.0125 | 0 | 0 | 0 | 168.1 | 0 | 0 | 0 | 0 | 5 | 148.9 |
| 267 | 112 | 0 | 0 | 0 | 0 | 29904 | 0 | 0 | 0.023724 | 0 | 0 | 0 | 0 | 163.4 | 1 | 20 | 0.4 | 0.5 | 3.5 | 115.3 |
| 267 | 112 | 0 | 0 | 0 | 0 | 29904 | 0 | 0 | 0.023724 | 0 | 0 | 0 | 0 | 163.4 | 1 | 20 | 0.4 | 0.5 | 3.5 | 146.3 |
| 267 | 114 | 0 | 0 | 0 | 0 | 30438 | 0 | 0 | 0.02342 | 0 | 0 | 0 | 0 | 178.1 | 1 | 20 | 0.4 | 0.5 | 3.5 | 103.8 |
| 267 | 114 | 0 | 0 | 0 | 0 | 30438 | 0 | 0 | 0.02342 | 0 | 0 | 0 | 0 | 178.1 | 1 | 20 | 0.4 | 0.5 | 4 | 109.6 |
| 270 | 113 | 0 | 0 | 0 | 0 | 30510 | 0 | 0 | 0.023293 | 10.1558 | 0 | 0 | 0 | 175.1 | 1 | 20 | 0.4 | 0.5 | 5 | 145.1 |
| 270 | 113 | 0 | 0 | 0 | 0 | 30510 | 0 | 0 | 0.023293 | 10.1558 | 0 | 0 | 0 | 175.1 | 1 | 20 | 0.4 | 0.5 | 5 | 146.3 |
| 265 | 112 | 0 | 0 | 0 | 0 | 29680 | 0 | 0 | 0.023838 | 22.763 | 0 | 0 | 0 | 175.1 | 1 | 20 | 0.4 | 0.5 | 5 | 217.6 |
| 272 | 112 | 0 | 0 | 0 | 0 | 30464 | 0 | 0 | 0.023173 | 0 | 0 | 0 | 0 | 192 | 2 | 20 | 0.4 | 1 | 3.5 | 183.1 |
| 272 | 112 | 0 | 0 | 0 | 0 | 30464 | 0 | 0 | 0.023173 | 0 | 0 | 0 | 0 | 192 | 2 | 20 | 0.4 | 1 | 4 | 123.8 |
| 268 | 113 | 0 | 0 | 0 | 0 | 30284 | 0 | 0 | 0.023403 | 10.5051 | 0 | 0 | 0 | 184.3 | 2 | 20 | 0.4 | 1 | 4.5 | 159.6 |
| 268 | 113 | 0 | 0 | 0 | 0 | 30284 | 0 | 0 | 0.023403 | 10.5051 | 0 | 0 | 0 | 184.3 | 2 | 20 | 0.4 | 1 | 4.5 | 157.6 |
| 600 | 150 | 0 | 0 | 0 | 0 | 90000 | 0.036228 | 435 | 0 | 0 | 200 | 0.001884 | 491.2 | 132.3 | 0.5 | 13 | 0.2 | 0.325 | 0.923 | 873.31 |
| 600 | 150 | 0 | 0 | 0 | 0 | 90000 | 0.036228 | 435 | 0 | 0 | 200 | 0.001884 | 491.2 | 145.5 | 1 | 13 | 0.2 | 0.65 | 0.923 | 1089.4 |
| 600 | 150 | 0 | 0 | 0 | 0 | 90000 | 0.036228 | 435 | 0 | 0 | 200 | 0.001884 | 491.2 | 175.2 | 2 | 13 | 0.2 | 1.3 | 0.923 | 1356.96 |
| 600 | 150 | 0 | 0 | 0 | 0 | 90000 | 0.036228 | 435 | 0 | 0 | 200 | 0.001884 | 491.2 | 198.6 | 3 | 13 | 0.2 | 1.95 | 0.923 | 1407.18 |
| 600 | 150 | 0 | 0 | 0 | 0 | 90000 | 0.036241 | 410 | 0 | 0 | 200 | 0.001884 | 414 | 151.4 | 1 | 13 | 0.2 | 0.65 | 0.554 | 1409.21 |
| 600 | 150 | 0 | 0 | 0 | 0 | 90000 | 0.036257 | 410 | 0 | 0 | 200 | 0.001884 | 414 | 151.4 | 1 | 13 | 0.2 | 0.65 | 0.739 | 1411.8 |
| 600 | 150 | 0 | 0 | 0 | 0 | 90000 | 0.036228 | 410 | 0 | 0 | 200 | 0.001884 | 414 | 151.4 | 1 | 13 | 0.2 | 0.65 | 0.923 | 1208.2 |
| 600 | 150 | 0 | 0 | 0 | 0 | 90000 | 0.03188 | 410 | 0 | 0 | 200 | 0.001884 | 414 | 151.4 | 1 | 13 | 0.2 | 0.65 | 0.923 | 1150.7 |
| 600 | 150 | 0 | 0 | 0 | 0 | 90000 | 0.033503 | 410 | 0 | 0 | 200 | 0.001884 | 414 | 151.4 | 1 | 13 | 0.2 | 0.65 | 0.923 | 1176.4 |
| 600 | 150 | 0 | 0 | 0 | 0 | 90000 | 0.036228 | 410 | 0 | 0 | 0 | 0 | 0 | 151.4 | 1 | 13 | 0.2 | 0.65 | 0.923 | 1056.3 |
| 600 | 150 | 0 | 0 | 0 | 0 | 90000 | 0.036228 | 410 | 0 | 0 | 100 | 0.003768 | 414 | 151.4 | 1 | 13 | 0.2 | 0.65 | 0.923 | 1392.24 |
| 600 | 150 | 0 | 0 | 0 | 0 | 90000 | 0.036228 | 410 | 0 | 0 | 250 | 0.001507 | 414 | 151.4 | 1 | 13 | 0.2 | 0.65 | 0.923 | 1180.6 |
| 600 | 150 | 0 | 0 | 0 | 0 | 90000 | 0.036228 | 410 | 0 | 0 | 200 | 0.001884 | 414 | 151.4 | 1 | 13 | 0.2 | 0.65 | 0.923 | 1115.1 |
| 600 | 150 | 0 | 0 | 0 | 0 | 90000 | 0.036228 | 410 | 0 | 0 | 200 | 0.001884 | 414 | 151.4 | 1 | 13 | 0.2 | 0.65 | 0.923 | 1358.7 |
| 350 | 250 | 0 | 0 | 0 | 0 | 87500 | 0.070153 | 675 | 0 | 0 | 0 | 0 | 0 | 151.8 | 2.65 | 13 | 0.2 | 1.7225 | 1.5 | 1293 |
| 350 | 250 | 0 | 0 | 0 | 0 | 87500 | 0.070153 | 675 | 0 | 0 | 0 | 0 | 0 | 151.8 | 2.65 | 13 | 0.2 | 1.7225 | 2.5 | 795 |
| 350 | 250 | 0 | 0 | 0 | 0 | 87500 | 0.070153 | 675 | 0 | 0 | 0 | 0 | 0 | 138.6 | 2.65 | 13 | 0.2 | 1.7225 | 2 | 966 |
| 350 | 250 | 0 | 0 | 0 | 0 | 87500 | 0.070153 | 675 | 0 | 0 | 0 | 0 | 0 | 138.6 | 2.65 | 13 | 0.2 | 1.7225 | 3 | 628 |
| 350 | 250 | 0 | 0 | 0 | 0 | 87500 | 0.061042 | 675 | 0 | 0 | 0 | 0 | 0 | 132 | 2.65 | 13 | 0.2 | 1.7225 | 2.5 | 719 |
| 350 | 250 | 0 | 0 | 0 | 0 | 87500 | 0.061042 | 675 | 0 | 0 | 0 | 0 | 0 | 132 | 2.65 | 13 | 0.2 | 1.7225 | 3 | 608 |
| 350 | 250 | 0 | 0 | 0 | 0 | 87500 | 0.055088 | 675 | 0 | 0 | 0 | 0 | 0 | 129.8 | 2.65 | 13 | 0.2 | 1.7225 | 1.5 | 913 |
| 350 | 250 | 0 | 0 | 0 | 0 | 87500 | 0.055088 | 675 | 0 | 0 | 0 | 0 | 0 | 129.8 | 2.65 | 13 | 0.2 | 1.7225 | 2.5 | 665 |
| 150 | 100 | 0 | 0 | 0 | 0 | 15000 | 0.012077 | 550 | 0 | 0 | 0 | 0 | 0 | 127 | 0 | 0 | 0 | 0 | 2.307692 | 18.2 |
| 150 | 100 | 0 | 0 | 0 | 0 | 15000 | 0.017391 | 550 | 0 | 0 | 0 | 0 | 0 | 127 | 0 | 0 | 0 | 0 | 2.307692 | 22.1 |
| 150 | 100 | 0 | 0 | 0 | 0 | 15000 | 0.012077 | 550 | 0 | 0 | 0 | 0 | 0 | 130 | 0 | 0 | 0 | 0 | 2.307692 | 21.95 |
| 150 | 100 | 0 | 0 | 0 | 0 | 15000 | 0.017391 | 550 | 0 | 0 | 0 | 0 | 0 | 130 | 0 | 0 | 0 | 0 | 2.307692 | 27 |
| 150 | 100 | 0 | 0 | 0 | 0 | 15000 | 0.012077 | 550 | 0 | 0 | 0 | 0 | 0 | 135 | 0.5 | 13 | 0.2 | 0.325 | 2.307692 | 26.9 |
| 150 | 100 | 0 | 0 | 0 | 0 | 15000 | 0.017391 | 550 | 0 | 0 | 0 | 0 | 0 | 135 | 0.5 | 13 | 0.2 | 0.325 | 2.307692 | 30.2 |
| 150 | 110 | 0 | 0 | 0 | 0 | 16500 | 0.01581 | 540 | 0 | 0 | 0 | 0 | 0 | 97 | 1 | 30 | 0.375 | 0.8 | 3.27 | 34 |
| 150 | 110 | 0 | 0 | 0 | 0 | 16500 | 0.023715 | 540 | 0 | 0 | 0 | 0 | 0 | 97 | 1 | 30 | 0.375 | 0.8 | 3.27 | 57.5 |
| 150 | 110 | 0 | 0 | 0 | 0 | 16500 | 0.01581 | 540 | 0 | 0 | 0 | 0 | 0 | 83.75 | 0 | 0 | 0 | 0 | 3.27 | 21.5 |
| 150 | 110 | 0 | 0 | 0 | 0 | 16500 | 0.01581 | 540 | 0 | 0 | 0 | 0 | 0 | 105.7 | 1 | 30 | 0.375 | 0.8 | 3.27 | 32.5 |
| 200 | 180 | 0 | 0 | 0 | 0 | 36000 | 0.026 | 494 | 0 | 0 | 0 | 0 | 0 | 179.6 | 0 | 0 | 0 | 0 | 3.5 | 66 |
| 200 | 177 | 0 | 0 | 0 | 0 | 35400 | 0.0417 | 494 | 0 | 0 | 0 | 0 | 0 | 186.6 | 0 | 0 | 0 | 0 | 3.5 | 87.5 |
| 200 | 177 | 0 | 0 | 0 | 0 | 35400 | 0.0417 | 494 | 0 | 0 | 0 | 0 | 0 | 186.8 | 0 | 0 | 0 | 0 | 3.5 | 85 |
| 200 | 180 | 0 | 0 | 0 | 0 | 36000 | 0.0252 | 494 | 0 | 0 | 0 | 0 | 0 | 185.6 | 0 | 0 | 0 | 0 | 3.4 | 82.5 |
| 300 | 150 | 0 | 0 | 0 | 0 | 45000 | 0.0612 | 494 | 0 | 0 | 0 | 0 | 0 | 141 | 0 | 0 | 0 | 0 | 3.5 | 144 |
| 300 | 150 | 0 | 0 | 0 | 0 | 45000 | 0.0872 | 494 | 0 | 0 | 0 | 0 | 0 | 133.6 | 0 | 0 | 0 | 0 | 3 | 147 |
| 300 | 150 | 0 | 0 | 0 | 0 | 45000 | 0.0872 | 494 | 0 | 0 | 0 | 0 | 0 | 152.8 | 1 | 13 | 0.2 | 0.65 | 3 | 384.8 |
| 300 | 150 | 0 | 0 | 0 | 0 | 45000 | 0.0872 | 494 | 0 | 0 | 0 | 0 | 0 | 153.9 | 1.5 | 13 | 0.2 | 0.975 | 3 | 455.2 |
| 910 | 155 | 300 | 190 | 460 | 228 | 238140 | 0 | 0 | 0.024414 | 10 | 0 | 0 | 0 | 193 | 2 | 13 | 0.2 | 1.3 | 2.5 | 1710 |
| 910 | 155 | 300 | 190 | 460 | 228 | 238140 | 0 | 0 | 0.024414 | 10 | 0 | 0 | 0 | 193 | 2 | 13 | 0.2 | 1.3 | 2.8 | 2230 |
| 910 | 155 | 300 | 190 | 460 | 228 | 238140 | 0 | 0 | 0.024414 | 10 | 0 | 0 | 0 | 193 | 2 | 13 | 0.2 | 1.3 | 2.3 | 1950 |
| 650 | 50 | 400 | 112.5 | 250 | 112.5 | 94375 | 0 | 0 | 0.056 | 0 | 0 | 0 | 0 | 161 | 2.5 | 13 | 0.2 | 1.625 | 3.33 | 430 |
| 650 | 50 | 400 | 112.5 | 250 | 112.5 | 94375 | 0 | 0 | 0.056 | 14.3 | 0 | 0 | 0 | 160 | 2.5 | 13 | 0.2 | 1.625 | 3.33 | 497 |
| 650 | 50 | 400 | 112.5 | 250 | 112.5 | 94375 | 0 | 0 | 0.056 | 7.15 | 0 | 0 | 0 | 149 | 2.5 | 13 | 0.2 | 1.625 | 3.33 | 428 |
| 650 | 50 | 400 | 112.5 | 250 | 112.5 | 94375 | 0 | 0 | 0.056 | 7.15 | 0 | 0 | 0 | 164 | 1.25 | 13 | 0.2 | 0.8125 | 3.33 | 337 |
| 650 | 50 | 400 | 112.5 | 250 | 112.5 | 94375 | 0 | 0 | 0.056 | 7.15 | 0 | 0 | 0 | 171 | 2.5 | 13 | 0.2 | 1.575 | 3.33 | 440 |
| 650 | 50 | 400 | 112.5 | 250 | 112.5 | 94375 | 0 | 0 | 0.056 | 7.15 | 0 | 0 | 0 | 157 | 2.5 | 30 | 0.5 | 1.5 | 3.33 | 330 |
| 650 | 50 | 400 | 112.5 | 250 | 112.5 | 94375 | 0 | 0 | 0.056 | 7.15 | 0 | 0 | 0 | 169 | 2.5 | 13 | 0.2 | 1.594 | 3.33 | 400 |
| 400 | 55 | 200 | 60 | 165 | 165 | 48850 | 0 | 0 | 0.0396 | 15.7509 | 0 | 0 | 0 | 151 | 0.9 | 17.5 | 0.15 | 1.05 | 3.8 | 234 |
| 400 | 52 | 200 | 60 | 165 | 165 | 48325 | 0 | 0 | 0.0539 | 18.9776 | 0 | 0 | 0 | 174 | 0.9 | 17.5 | 0.15 | 1.05 | 3.8 | 267 |
| 400 | 60 | 200 | 60 | 165 | 165 | 49725 | 0 | 0 | 0.0467 | 18.7329 | 0 | 0 | 0 | 162 | 2.5 | 9 | 0.15 | 1.5 | 3.8 | 408 |
| 400 | 60 | 200 | 60 | 165 | 165 | 49725 | 0 | 0 | 0.0467 | 18.5248 | 0 | 0 | 0 | 176 | 0.9 | 17.5 | 0.15 | 1.05 | 3.8 | 347 |
| 400 | 60 | 200 | 60 | 165 | 165 | 49725 | 0 | 0 | 0.0467 | 18.576 | 0 | 0 | 0 | 183 | 0.9 | 17.5 | 0.15 | 1.05 | 4.4 | 292 |
| 460 | 50 | 230 | 80 | 220 | 130 | 59500 | 0.114843 | 617 | 0 | 0 | 0 | 0 | 0 | 148 | 2 | 13 | 0.2 | 1.3 | 4 | 260 |
| 460 | 50 | 230 | 80 | 220 | 130 | 59500 | 0.153124 | 617 | 0 | 0 | 0 | 0 | 0 | 144 | 1 | 13 | 0.2 | 0.65 | 4 | 175 |
| 460 | 50 | 230 | 80 | 220 | 130 | 59500 | 0.153124 | 617 | 0 | 0 | 0 | 0 | 0 | 146 | 2 | 13 | 0.2 | 1.3 | 4 | 190 |
| 460 | 50 | 230 | 80 | 220 | 130 | 59500 | 0.153124 | 617 | 0 | 0 | 0 | 0 | 0 | 152 | 3 | 13 | 0.2 | 1.95 | 4 | 300 |
| 380 | 50 | 170 | 70 | 165 | 120 | 41200 | 0.107956 | 617 | 0 | 0 | 0 | 0 | 0 | 147 | 2 | 13 | 0.2 | 1.3 | 6 | 135 |
| 380 | 50 | 170 | 70 | 165 | 120 | 41200 | 0.107956 | 617 | 0 | 0 | 0 | 0 | 0 | 149 | 2 | 13 | 0.2 | 1.3 | 8 | 110 |
| 380 | 50 | 170 | 70 | 165 | 120 | 41200 | 0.124603 | 617 | 0 | 0 | 0 | 0 | 0 | 146 | 2 | 13 | 0.2 | 1.3 | 4 | 215 |
| 380 | 50 | 170 | 70 | 165 | 120 | 41200 | 0.124603 | 617 | 0 | 0 | 0 | 0 | 0 | 147 | 2 | 13 | 0.2 | 1.3 | 6 | 155 |
| 650 | 50 | 500 | 75 | 500 | 75 | 100000 | 0 | 0 | 0.027097 | 19.2 | 0 | 0 | 0 | 125 | 1 | 15 | 0.2 | 0.75 | 3.2 | 330 |
| 650 | 50 | 500 | 75 | 500 | 75 | 100000 | 0 | 0 | 0.027097 | 19.2 | 0 | 0 | 0 | 126 | 1 | 15 | 0.2 | 0.75 | 3.2 | 355 |
| 650 | 50 | 500 | 75 | 500 | 75 | 100000 | 0 | 0 | 0.027097 | 19.2 | 0 | 0 | 0 | 135 | 1 | 15 | 0.2 | 0.75 | 3.2 | 360 |
| 650 | 50 | 500 | 75 | 500 | 75 | 100000 | 0 | 0 | 0.027097 | 19.2 | 0 | 0 | 0 | 122 | 1 | 15 | 0.2 | 0.75 | 2.5 | 455.5 |
| 650 | 50 | 500 | 75 | 500 | 75 | 100000 | 0 | 0 | 0.027097 | 19.2 | 0 | 0 | 0 | 140 | 1 | 25 | 0.2 | 1.25 | 3.5 | 422.5 |
| 650 | 50 | 500 | 75 | 500 | 75 | 100000 | 0 | 0 | 0.027097 | 19.2 | 0 | 0 | 0 | 140 | 1 | 25 | 0.2 | 1.25 | 4.5 | 390.5 |
| 650 | 50 | 500 | 75 | 500 | 75 | 100000 | 0 | 0 | 0.027097 | 19.2 | 0 | 0 | 0 | 122 | 1.5 | 20 | 0.2 | 1.5 | 2.5 | 521.5 |
| 650 | 50 | 500 | 75 | 500 | 75 | 100000 | 0 | 0 | 0.027097 | 19.2 | 0 | 0 | 0 | 122 | 1 | 15 | 0.2 | 0.75 | 1.8 | 582 |
| 700 | 50 | 500 | 125 | 500 | 130 | 149750 | 0 | 0 | 0.0525 | 0 | 0 | 0 | 0 | 185.5 | 2 | 13 | 0.2 | 1.3 | 2.5 | 526.5 |
| 700 | 50 | 500 | 125 | 500 | 130 | 149750 | 0 | 0 | 0.0525 | 9.4798 | 0 | 0 | 0 | 189.8 | 2 | 13 | 0.2 | 1.3 | 2.5 | 716.5 |
| 700 | 50 | 500 | 125 | 500 | 130 | 149750 | 0 | 0 | 0.0525 | 0 | 0 | 0 | 0 | 188.5 | 2 | 13 | 0.2 | 1.3 | 3.4 | 404 |
| 700 | 50 | 500 | 125 | 500 | 130 | 149750 | 0 | 0 | 0.0525 | 10.65776 | 0 | 0 | 0 | 182.3 | 2 | 13 | 0.2 | 1.3 | 3.4 | 477 |
| 1000 | 40 | 500 | 125 | 500 | 130 | 157300 | 0 | 0 | 0.044681 | 0 | 0 | 0 | 0 | 186.5 | 2 | 13 | 0.2 | 1.3 | 1.7 | 869 |
| 1000 | 40 | 500 | 125 | 500 | 130 | 157300 | 0 | 0 | 0.044681 | 10.14622 | 0 | 0 | 0 | 186.5 | 2 | 13 | 0.2 | 1.3 | 1.7 | 968.5 |
| 1000 | 40 | 500 | 125 | 500 | 130 | 157300 | 0 | 0 | 0.044681 | 0 | 0 | 0 | 0 | 186.5 | 2 | 13 | 0.2 | 1.3 | 2.2 | 598 |
| 1000 | 40 | 500 | 125 | 500 | 130 | 157300 | 0 | 0 | 0.044681 | 10.14622 | 0 | 0 | 0 | 186.5 | 2 | 13 | 0.2 | 1.3 | 2.2 | 764.5 |
| 700 | 50 | 500 | 125 | 500 | 130 | 149750 | 0 | 0 | 0.0525 | 0 | 0 | 0 | 0 | 174.5 | 1 | 13 | 0.2 | 0.65 | 2.5 | 488 |
| 700 | 50 | 500 | 125 | 500 | 130 | 149750 | 0 | 0 | 0.0525 | 9.4798 | 0 | 0 | 0 | 181.3 | 1 | 13 | 0.2 | 0.65 | 2.5 | 590 |
| 700 | 50 | 500 | 125 | 500 | 130 | 149750 | 0 | 0 | 0.0525 | 0 | 0 | 0 | 0 | 188.2 | 1.5 | 13 | 0.2 | 0.975 | 2.5 | 614 |
| 700 | 50 | 500 | 125 | 500 | 130 | 149750 | 0 | 0 | 0.0525 | 9.4798 | 0 | 0 | 0 | 183.6 | 1.5 | 13 | 0.2 | 0.975 | 2.5 | 712 |
| 700 | 50 | 500 | 125 | 500 | 130 | 149750 | 0 | 0 | 0.0525 | 0 | 0 | 0 | 0 | 168.9 | 1 | 13 | 0.2 | 0.65 | 3.4 | 279 |
| 700 | 50 | 500 | 125 | 500 | 130 | 149750 | 0 | 0 | 0.0525 | 10.65776 | 0 | 0 | 0 | 167.2 | 1 | 13 | 0.2 | 0.65 | 3.4 | 374 |
| 700 | 50 | 500 | 125 | 500 | 130 | 149750 | 0 | 0 | 0.0525 | 0 | 0 | 0 | 0 | 193 | 1.5 | 13 | 0.2 | 0.975 | 3.4 | 308 |
| 700 | 50 | 500 | 125 | 500 | 130 | 149750 | 0 | 0 | 0.0525 | 10.65776 | 0 | 0 | 0 | 189.2 | 1.5 | 13 | 0.2 | 0.975 | 3.4 | 437 |
| 380 | 65 | 270 | 67.5 | 230 | 127.5 | 59575 | 0 | 0 | 0.045397 | 12.55 | 0 | 0 | 0 | 190 | 2.5 | 20 | 0.3 | 1.666667 | 2.5 | 430 |
| 380 | 65 | 270 | 67.5 | 230 | 127.5 | 59575 | 0 | 0 | 0.045397 | 12.55 | 0 | 0 | 0 | 190 | 2.5 | 20 | 0.3 | 1.666667 | 2.5 | 431 |
| 380 | 65 | 270 | 67.5 | 230 | 127.5 | 59575 | 0 | 0 | 0.045397 | 12.3 | 0 | 0 | 0 | 207 | 2 | 13 | 0.2 | 1.3 | 2.5 | 507 |
| 380 | 65 | 270 | 67.5 | 230 | 127.5 | 59575 | 0 | 0 | 0.045397 | 12.22 | 75 | 0.011594 | 561 | 190 | 2.5 | 20 | 0.3 | 1.666667 | 2.5 | 544 |
| 380 | 65 | 270 | 67.5 | 230 | 127.5 | 59575 | 0 | 0 | 0.045397 | 12.3 | 75 | 0.011594 | 561 | 207 | 2 | 13 | 0.2 | 1.3 | 2.5 | 629.5 |
| 380 | 65 | 270 | 67.5 | 230 | 127.5 | 59575 | 0.103941 | 551 | 0 | 0 | 0 | 0 | 0 | 190 | 2.5 | 20 | 0.3 | 1.666667 | 2.5 | 454.5 |
| 380 | 65 | 270 | 67.5 | 230 | 127.5 | 59575 | 0.103941 | 551 | 0 | 0 | 0 | 0 | 0 | 207 | 2 | 13 | 0.2 | 1.3 | 2.5 | 447.5 |
| 380 | 65 | 270 | 67.5 | 230 | 127.5 | 59575 | 0.103941 | 551 | 0 | 0 | 0 | 0 | 0 | 168 | 0 | 0 | 0 | 0 | 2.5 | 180.5 |
| 160 | 100 | 220 | 50 | 0 | 0 | 22000 | 0.076961 | 617 | 0 | 0 | 0 | 0 | 0 | 127 | 1 | 13 | 0.2 | 0.65 | 3.5 | 70.5 |
| 160 | 100 | 220 | 50 | 0 | 0 | 22000 | 0.076961 | 617 | 0 | 0 | 0 | 0 | 0 | 148.5 | 2 | 13 | 0.2 | 1.3 | 3.5 | 122.75 |
| 160 | 100 | 220 | 50 | 0 | 0 | 22000 | 0.076961 | 617 | 0 | 0 | 0 | 0 | 0 | 148.5 | 2 | 13 | 0.2 | 1.3 | 2.5 | 209.5 |
| 160 | 100 | 220 | 50 | 0 | 0 | 22000 | 0.076961 | 617 | 0 | 0 | 0 | 0 | 0 | 148.5 | 2 | 13 | 0.2 | 1.3 | 4.3 | 100 |
| 160 | 100 | 220 | 50 | 0 | 0 | 22000 | 0.076961 | 617 | 0 | 0 | 0 | 0 | 0 | 139 | 2 | 13 | 0.2 | 1.3 | 3.5 | 119.25 |
| 160 | 100 | 220 | 50 | 0 | 0 | 22000 | 0.076961 | 617 | 0 | 0 | 0 | 0 | 0 | 130 | 2 | 13 | 0.2 | 1.3 | 3.5 | 111.5 |
| 400 | 60 | 140 | 65 | 140 | 85 | 36000 | 0.051343 | 365 | 0 | 0 | 0 | 0 | 0 | 135.1 | 1 | 9 | 0.175 | 0.582214 | 2.5 | 149 |
| 400 | 60 | 140 | 65 | 140 | 85 | 36000 | 0.051343 | 365 | 0 | 0 | 0 | 0 | 0 | 140 | 1 | 9 | 0.175 | 0.582214 | 2.5 | 93 |
| 400 | 60 | 140 | 65 | 140 | 85 | 36000 | 0.051343 | 365 | 0 | 0 | 0 | 0 | 0 | 135.1 | 1 | 9 | 0.175 | 0.582214 | 2.3 | 114 |
| 400 | 60 | 140 | 65 | 140 | 85 | 36000 | 0.051343 | 365 | 0 | 0 | 0 | 0 | 0 | 140 | 1 | 9 | 0.175 | 0.582214 | 2.3 | 123 |
| 400 | 50 | 150 | 80 | 150 | 80 | 36000 | 0.056071 | 535 | 0 | 0 | 0 | 0 | 0 | 141.05 | 0 | 0 | 0 | 0 | 2.857143 | 91 |
| 400 | 50 | 150 | 80 | 150 | 80 | 36000 | 0.056071 | 535 | 0 | 0 | 0 | 0 | 0 | 147.69 | 0.8 | 13 | 0.2 | 0.52 | 2.857143 | 340 |
| 400 | 50 | 150 | 80 | 150 | 80 | 36000 | 0.056071 | 535 | 0 | 0 | 0 | 0 | 0 | 146.71 | 1.6 | 13 | 0.2 | 1.04 | 2.857143 | 531 |
| 350 | 60 | 200 | 80 | 200 | 80 | 43400 | 0.116667 | 1100 | 0 | 0 | 125 | 0.020933 | 594 | 185.2 | 2 | 15 | 0.2 | 1.5 | 3.2 | 540 |
| 350 | 60 | 200 | 80 | 200 | 80 | 43400 | 0.116667 | 1100 | 0 | 0 | 125 | 0.020933 | 594 | 184.2 | 2 | 15 | 0.2 | 1.5 | 3.2 | 578 |
| 350 | 60 | 200 | 80 | 200 | 80 | 43400 | 0.116667 | 1100 | 0 | 0 | 125 | 0.020933 | 594 | 154.3 | 0 | 0 | 0 | 0 | 3.5 | 337 |
| 350 | 60 | 200 | 80 | 200 | 80 | 43400 | 0.116667 | 1100 | 0 | 0 | 125 | 0.020933 | 594 | 172.9 | 0 | 0 | 0 | 0 | 3.5 | 369 |
| 350 | 60 | 200 | 80 | 200 | 80 | 43400 | 0.116667 | 1100 | 0 | 0 | 200 | 0.013083 | 594 | 169.7 | 2 | 15 | 0.2 | 1.5 | 3.5 | 443 |
| 350 | 60 | 200 | 80 | 200 | 80 | 43400 | 0.116667 | 1100 | 0 | 0 | 200 | 0.013083 | 594 | 162.8 | 2 | 15 | 0.2 | 1.5 | 3.5 | 516 |
| 350 | 60 | 200 | 80 | 200 | 80 | 43400 | 0.116667 | 1100 | 0 | 0 | 200 | 0.013083 | 594 | 169.8 | 1 | 15 | 0.2 | 0.75 | 3.5 | 445 |
| 350 | 60 | 200 | 80 | 200 | 80 | 43400 | 0.116667 | 1100 | 0 | 0 | 200 | 0.013083 | 594 | 168.2 | 0 | 0 | 0 | 0 | 3.5 | 300 |
| 350 | 60 | 200 | 80 | 200 | 80 | 43400 | 0.116667 | 1100 | 0 | 0 | 300 | 0.008722 | 594 | 170.4 | 2 | 15 | 0.2 | 1.5 | 3.5 | 446 |
| 350 | 60 | 200 | 80 | 200 | 80 | 43400 | 0.116667 | 1100 | 0 | 0 | 300 | 0.008722 | 594 | 165.6 | 1 | 15 | 0.2 | 0.75 | 3.5 | 398 |
| 350 | 60 | 200 | 80 | 200 | 80 | 43400 | 0.116667 | 1100 | 0 | 0 | 300 | 0.008722 | 594 | 174.9 | 0 | 0 | 0 | 0 | 3.5 | 253 |
| 350 | 60 | 200 | 80 | 200 | 80 | 43400 | 0.116667 | 1100 | 0 | 0 | 0 | 0 | 0 | 160.4 | 2 | 15 | 0.2 | 1.5 | 3.5 | 251 |
| 350 | 60 | 200 | 80 | 200 | 80 | 43400 | 0.116667 | 1100 | 0 | 0 | 0 | 0 | 0 | 188.4 | 2 | 15 | 0.2 | 1.5 | 3.5 | 320 |
| 350 | 60 | 200 | 80 | 200 | 80 | 43400 | 0.116667 | 1100 | 0 | 0 | 0 | 0 | 0 | 177.5 | 2 | 15 | 0.2 | 1.5 | 3.5 | 358 |
| 350 | 60 | 200 | 80 | 200 | 80 | 43400 | 0.116667 | 1100 | 0 | 0 | 0 | 0 | 0 | 161.9 | 1 | 15 | 0.2 | 0.75 | 3.5 | 266 |
| 350 | 60 | 200 | 80 | 200 | 80 | 43400 | 0.116667 | 1100 | 0 | 0 | 0 | 0 | 0 | 174.8 | 1 | 15 | 0.2 | 0.75 | 3.5 | 199 |
| 350 | 60 | 200 | 80 | 200 | 80 | 43400 | 0.116667 | 1100 | 0 | 0 | 0 | 0 | 0 | 186.5 | 1 | 15 | 0.2 | 0.75 | 3.5 | 308 |
| 350 | 60 | 200 | 80 | 200 | 80 | 43400 | 0.116667 | 1100 | 0 | 0 | 0 | 0 | 0 | 160.8 | 0 | 0 | 0 | 0 | 3.5 | 82 |
| 350 | 60 | 200 | 80 | 200 | 80 | 43400 | 0.116667 | 1100 | 0 | 0 | 0 | 0 | 0 | 169.6 | 0 | 0 | 0 | 0 | 3.5 | 63 |
| 350 | 60 | 200 | 80 | 200 | 80 | 43400 | 0.116667 | 1100 | 0 | 0 | 0 | 0 | 0 | 166.6 | 0 | 0 | 0 | 0 | 3.5 | 51 |
| 300 | 50 | 300 | 35 | 100 | 70 | 27250 | 0.048494 | 394 | 0.021622 | 4.586862 | 0 | 0 | 0 | 137 | 1.6 | 12 | 0.16 | 1.2 | 2 | 193.5 |
| 300 | 50 | 300 | 35 | 100 | 70 | 27250 | 0.048494 | 394 | 0.021622 | 4.586862 | 0 | 0 | 0 | 132 | 1.6 | 12 | 0.16 | 1.2 | 3 | 170 |
| 300 | 50 | 300 | 35 | 100 | 70 | 27250 | 0.048494 | 394 | 0.032432 | 13.76059 | 90 | 0.01256 | 586 | 125 | 1.6 | 12 | 0.16 | 1.2 | 2 | 318.5 |
| 300 | 50 | 300 | 35 | 100 | 70 | 27250 | 0.048494 | 394 | 0.032432 | 13.76059 | 180 | 0.00628 | 586 | 126 | 1.6 | 12 | 0.16 | 1.2 | 2 | 282 |
| 300 | 50 | 300 | 35 | 100 | 70 | 27250 | 0.048494 | 394 | 0.032432 | 6.880294 | 90 | 0.01256 | 586 | 127 | 1.6 | 12 | 0.16 | 1.2 | 2 | 314 |
| 300 | 50 | 300 | 35 | 100 | 70 | 27250 | 0.048494 | 394 | 0.032432 | 6.880294 | 180 | 0.00628 | 586 | 127 | 1.6 | 12 | 0.16 | 1.2 | 2 | 249 |
| 300 | 50 | 300 | 35 | 100 | 70 | 27250 | 0.048494 | 394 | 0.032432 | 0 | 90 | 0.01256 | 586 | 140 | 1.6 | 12 | 0.16 | 1.2 | 2 | 291.5 |
| 300 | 50 | 300 | 35 | 100 | 70 | 27250 | 0.048494 | 394 | 0.032432 | 0 | 180 | 0.00628 | 586 | 140 | 1.6 | 12 | 0.16 | 1.2 | 2 | 250 |
| 300 | 50 | 300 | 35 | 100 | 70 | 27250 | 0.048494 | 394 | 0.032432 | 13.76059 | 90 | 0.01256 | 586 | 127 | 1.6 | 12 | 0.16 | 1.2 | 1.1 | 285.5 |
| 300 | 50 | 300 | 35 | 100 | 70 | 27250 | 0.048494 | 394 | 0.032432 | 13.76059 | 90 | 0.01256 | 586 | 127 | 1.6 | 12 | 0.16 | 1.2 | 3 | 367.5 |
| 500 | 60 | 600 | 55 | 140 | 155 | 72100 | 0.095417 | 389 | 0 | 0 | 100 | 0.00471 | 302 | 152.5 | 1.5 | 13 | 0.22 | 0.886364 | 1 | 755 |
| 500 | 60 | 600 | 55 | 140 | 155 | 72100 | 0.095417 | 389 | 0 | 0 | 100 | 0.00471 | 302 | 145.1 | 1.5 | 13 | 0.22 | 0.886364 | 2 | 556 |
| 500 | 60 | 600 | 55 | 140 | 155 | 72100 | 0.095417 | 389 | 0 | 0 | 100 | 0.00471 | 302 | 132.7 | 1.5 | 13 | 0.22 | 0.886364 | 3 | 410 |
| 500 | 60 | 600 | 55 | 140 | 155 | 72100 | 0.095417 | 389 | 0 | 0 | 100 | 0.00471 | 302 | 144.5 | 1.5 | 13 | 0.22 | 0.886364 | 4 | 360 |
| 500 | 60 | 600 | 55 | 140 | 155 | 72100 | 0.095417 | 389 | 0 | 0 | 100 | 0.008373 | 342 | 127.1 | 1.5 | 13 | 0.22 | 0.886364 | 1 | 695 |
| 500 | 60 | 600 | 55 | 140 | 155 | 72100 | 0.095417 | 389 | 0 | 0 | 200 | 0.002355 | 302 | 134.3 | 1.5 | 13 | 0.22 | 0.886364 | 2 | 500 |
| 500 | 60 | 600 | 55 | 140 | 155 | 72100 | 0.095417 | 389 | 0 | 0 | 0 | 0 | 0 | 148.9 | 1.5 | 13 | 0.22 | 0.886364 | 2 | 484 |
| 500 | 60 | 600 | 55 | 140 | 155 | 72100 | 0.095417 | 389 | 0 | 0 | 150 | 0.00314 | 302 | 154.5 | 1.5 | 13 | 0.22 | 0.886364 | 2 | 507 |
| 500 | 60 | 600 | 55 | 140 | 155 | 72100 | 0.110849 | 389 | 0 | 0 | 150 | 0.00314 | 302 | 143.5 | 1.5 | 13 | 0.22 | 0.886364 | 2 | 539 |
| 500 | 60 | 600 | 55 | 140 | 155 | 72100 | 0.127975 | 389 | 0 | 0 | 150 | 0.00314 | 302 | 151.2 | 1.5 | 13 | 0.22 | 0.886364 | 2 | 566 |
| 500 | 60 | 600 | 55 | 140 | 155 | 72100 | 0.095417 | 389 | 0 | 0 | 100 | 0.00471 | 302 | 133.9 | 1.5 | 13 | 0.22 | 0.886364 | 1 | 556 |
| 500 | 60 | 600 | 55 | 140 | 155 | 72100 | 0.095417 | 389 | 0 | 0 | 100 | 0.00471 | 302 | 128.4 | 1.5 | 13 | 0.22 | 0.886364 | 3 | 456 |
| 250 | 50 | 150 | 50 | 150 | 50 | 22500 | 0.035726 | 469 | 0 | 0 | 0 | 0 | 0 | 145 | 0 | 0 | 0 | 0 | 4.4 | 18.91 |
| 250 | 50 | 150 | 50 | 150 | 50 | 22500 | 0.013956 | 470 | 0 | 0 | 0 | 0 | 0 | 121 | 0 | 0 | 0 | 0 | 3.111111 | 22.845 |
| 250 | 50 | 150 | 50 | 150 | 50 | 22500 | 0.020186 | 470 | 0 | 0 | 0 | 0 | 0 | 121 | 0 | 0 | 0 | 0 | 3.125 | 21.56 |
| 250 | 50 | 150 | 50 | 150 | 50 | 22500 | 0.027598 | 470 | 0 | 0 | 0 | 0 | 0 | 121 | 0 | 0 | 0 | 0 | 3.139013 | 25.3 |
| 250 | 50 | 150 | 50 | 150 | 50 | 22500 | 0.036209 | 470 | 0 | 0 | 0 | 0 | 0 | 121 | 0 | 0 | 0 | 0 | 3.153153 | 33.405 |
| 250 | 50 | 150 | 50 | 150 | 50 | 22500 | 0.027598 | 470 | 0 | 0 | 0 | 0 | 0 | 143 | 2 | 30 | 0.55 | 1.090909 | 3.139013 | 62.655 |
| 250 | 50 | 150 | 50 | 150 | 50 | 22500 | 0.036209 | 470 | 0 | 0 | 0 | 0 | 0 | 143 | 2 | 30 | 0.55 | 1.090909 | 3.153153 | 60.09 |
| 500 | 100 | 300 | 50 | 200 | 115 | 71500 | 0.021102 | 778.4 | 0 | 0 | 0 | 0 | 0 | 133.5 | 2 | 13 | 0.2 | 1.3 | 1.6 | 330.7 |
| 500 | 100 | 300 | 50 | 200 | 115 | 71500 | 0.034608 | 778.4 | 0 | 0 | 0 | 0 | 0 | 133.5 | 2 | 13 | 0.2 | 1.3 | 1.6 | 375.1 |
| 500 | 100 | 300 | 50 | 200 | 115 | 71500 | 0.021102 | 778.4 | 0 | 0 | 0 | 0 | 0 | 133.5 | 2 | 13 | 0.2 | 1.3 | 2.6 | 222.1 |
| 500 | 100 | 300 | 50 | 200 | 115 | 71500 | 0.034608 | 778.4 | 0 | 0 | 0 | 0 | 0 | 133.5 | 2 | 13 | 0.2 | 1.3 | 2.6 | 288.05 |
| 500 | 100 | 300 | 50 | 200 | 115 | 71500 | 0.021102 | 778.4 | 0 | 0 | 150 | 0.006699 | 447.2 | 136.8 | 2 | 13 | 0.2 | 1.3 | 1.6 | 400.7 |
| 500 | 100 | 300 | 50 | 200 | 115 | 71500 | 0.034608 | 778.4 | 0 | 0 | 150 | 0.006699 | 447.2 | 136.8 | 2 | 13 | 0.2 | 1.3 | 1.6 | 487.6 |
| 500 | 100 | 300 | 50 | 200 | 115 | 71500 | 0.034608 | 778.4 | 0 | 0 | 150 | 0.006699 | 447.2 | 136.8 | 2 | 13 | 0.2 | 1.3 | 2.6 | 380.2 |
| 889 | 76.2 | 711 | 128.6 | 711 | 165.1 | 254182.6 | 0 | 0 | 0.073509 | 25 | 0 | 0 | 0 | 137 | 2 | 13 | 0.2 | 1.3 | 3.5 | 1242 |
| 889 | 76.2 | 711 | 128.6 | 711 | 165.1 | 254182.6 | 0 | 0 | 0.073509 | 25 | 0 | 0 | 0 | 158 | 2 | 13 | 0.2 | 1.3 | 3.5 | 1265 |
| 889 | 76.2 | 711 | 128.6 | 711 | 165.1 | 254182.6 | 0 | 0 | 0.055132 | 19.3 | 0 | 0 | 0 | 152 | 2 | 13 | 0.2 | 1.3 | 3.5 | 1236 |
| 889 | 101.6 | 737 | 128.6 | 737 | 165.1 | 276939.4 | 0 | 0 | 0.055132 | 22.9 | 0 | 0 | 0 | 140 | 2 | 13 | 0.2 | 1.3 | 3.5 | 1491 |
| 1092 | 76.2 | 711 | 128.6 | 711 | 165.1 | 269651.2 | 0 | 0 | 0.058819 | 23.5 | 0 | 0 | 0 | 160 | 2 | 13 | 0.2 | 1.3 | 3.5 | 1410 |
| 1092 | 76.2 | 711 | 128.6 | 711 | 165.1 | 269651.2 | 0 | 0 | 0.058819 | 23.5 | 203.1 | 0.012985 | 483.3 | 158 | 2 | 13 | 0.2 | 1.3 | 3.5 | 2567 |
| 400 | 60 | 200 | 60 | 165 | 165 | 49725 | 0 | 0 | 0.0363 | 15.544 | 0 | 0 | 0 | 134 | 0 | 0 | 0 | 0 | 3.8 | 134 |
| 400 | 60 | 200 | 60 | 165 | 165 | 49725 | 0 | 0 | 0.0467 | 18.358 | 0 | 0 | 0 | 134 | 0 | 0 | 0 | 0 | 3.8 | 147 |
| 400 | 60 | 200 | 60 | 165 | 165 | 49725 | 0 | 0 | 0.0467 | 18.304 | 0 | 0 | 0 | 177 | 0.9 | 17.5 | 0.15 | 1.05 | 3.8 | 326 |
| 400 | 60 | 200 | 60 | 165 | 165 | 49725 | 0 | 0 | 0.0467 | 18.513 | 0 | 0 | 0 | 179 | 0.9 | 17.5 | 0.15 | 1.05 | 3.8 | 299 |
| 400 | 60 | 200 | 60 | 165 | 165 | 49725 | 0 | 0 | 0.0467 | 18.7812 | 0 | 0 | 0 | 185 | 0.9 | 17.5 | 0.15 | 1.05 | 4.1 | 301 |
| 400 | 60 | 200 | 60 | 165 | 165 | 49725 | 0 | 0 | 0.0467 | 18.6504 | 0 | 0 | 0 | 174 | 0.9 | 17.5 | 0.15 | 1.05 | 3.5 | 324 |
| 400 | 40 | 200 | 60 | 165 | 165 | 46225 | 0 | 0 | 0.0467 | 18.4808 | 0 | 0 | 0 | 189 | 0.9 | 17.5 | 0.15 | 1.05 | 3.8 | 210 |
| 700 | 40 | 200 | 60 | 165 | 165 | 58225 | 0 | 0 | 0.048 | 17.766 | 0 | 0 | 0 | 175 | 0.9 | 17.5 | 0.15 | 1.05 | 3.8 | 316 |
| 700 | 60 | 200 | 60 | 165 | 165 | 67725 | 0 | 0 | 0.0539 | 18.7502 | 0 | 0 | 0 | 169 | 0.9 | 17.5 | 0.15 | 1.05 | 3.8 | 464 |
| 700 | 60 | 200 | 60 | 165 | 165 | 67725 | 0 | 0 | 0.0539 | 17.787 | 0 | 0 | 0 | 172 | 2.5 | 9 | 0.15 | 1.5 | 3.8 | 521 |
| 700 | 60 | 200 | 60 | 165 | 165 | 67725 | 0 | 0 | 0.0539 | 18.748 | 0 | 0 | 0 | 183 | 0.9 | 30 | 0.4 | 0.675 | 3.8 | 476 |
| 495 | 40 | 250 | 80 | 250 | 150 | 68100 | 0.222534 | 617 | 0 | 0 | 0 | 0 | 0 | 160.1 | 1.5 | 16 | 0.2 | 1.4 | 2.5 | 415.1 |
| 495 | 40 | 250 | 80 | 250 | 150 | 68100 | 0.222534 | 617 | 0 | 0 | 0 | 0 | 0 | 160.1 | 1.5 | 16 | 0.2 | 1.4 | 3.5 | 355.6 |
| 495 | 40 | 250 | 80 | 250 | 150 | 68100 | 0.222534 | 617 | 0 | 0 | 0 | 0 | 0 | 160.1 | 1.5 | 16 | 0.2 | 1.4 | 3.5 | 387.7 |
| 675 | 40 | 250 | 80 | 250 | 150 | 75300 | 0.158444 | 617 | 0 | 0 | 0 | 0 | 0 | 166.6 | 1.5 | 16 | 0.2 | 1.4 | 3.5 | 347.6 |
| 675 | 40 | 250 | 80 | 250 | 150 | 75300 | 0.158444 | 617 | 0 | 0 | 0 | 0 | 0 | 166.6 | 1.5 | 16 | 0.2 | 1.4 | 3.5 | 321.1 |
| 495 | 40 | 250 | 80 | 250 | 150 | 68100 | 0.222534 | 617 | 0 | 0 | 0 | 0 | 0 | 162.4 | 1.5 | 16 | 0.2 | 1.4 | 2.5 | 445.9 |
| 495 | 40 | 250 | 80 | 250 | 150 | 68100 | 0.222534 | 617 | 0 | 0 | 0 | 0 | 0 | 162.4 | 1.5 | 16 | 0.2 | 1.4 | 3.5 | 371.8 |
| 675 | 40 | 250 | 80 | 250 | 150 | 75300 | 0.158444 | 617 | 0 | 0 | 0 | 0 | 0 | 162.4 | 1.5 | 16 | 0.2 | 1.4 | 3.5 | 439.7 |
| 495 | 40 | 250 | 80 | 250 | 150 | 68100 | 0.222534 | 617 | 0 | 0 | 0 | 0 | 0 | 169.9 | 1 | 20 | 0.2 | 1 | 2.5 | 427.7 |
| 495 | 40 | 250 | 80 | 250 | 150 | 68100 | 0.222534 | 617 | 0 | 0 | 0 | 0 | 0 | 169.9 | 1 | 20 | 0.2 | 1 | 3.5 | 340.3 |
| 675 | 40 | 250 | 80 | 250 | 150 | 75300 | 0.158444 | 617 | 0 | 0 | 0 | 0 | 0 | 169.9 | 1 | 20 | 0.2 | 1 | 3.5 | 354.5 |
| 600 | 40 | 225 | 72.5 | 225 | 191 | 72747.5 | 0 | 0 | 0.0544 | 22.3881 | 0 | 0 | 0 | 152.3 | 2.5 | 9 | 0.175 | 1.285714 | 3.5 | 463.8 |
| 600 | 40 | 225 | 72.5 | 225 | 191 | 72747.5 | 0 | 0 | 0.0544 | 11.2676 | 0 | 0 | 0 | 148.2 | 2.11 | 14 | 0.2 | 1.477 | 3.5 | 485.9 |
| 600 | 40 | 225 | 72.5 | 225 | 191 | 72747.5 | 0 | 0 | 0.0544 | 22.4543 | 0 | 0 | 0 | 142.4 | 1.05 | 14 | 0.2 | 0.735 | 3.5 | 467.4 |
| 600 | 40 | 225 | 72.5 | 225 | 191 | 72747.5 | 0 | 0 | 0.0544 | 11.1232 | 0 | 0 | 0 | 166 | 2.11 | 14 | 0.2 | 1.477 | 3.5 | 526.2 |
| 600 | 40 | 225 | 72.5 | 225 | 191 | 72747.5 | 0 | 0 | 0.0544 | 22.365 | 0 | 0 | 0 | 171.8 | 2.11 | 14 | 0.2 | 1.477 | 3.5 | 572.8 |
| 600 | 40 | 225 | 72.5 | 225 | 191 | 72747.5 | 0 | 0 | 0.0544 | 22.3272 | 0 | 0 | 0 | 177.7 | 1.05 | 14 | 0.2 | 0.735 | 3.5 | 379.6 |
| 600 | 40 | 225 | 72.5 | 225 | 191 | 72747.5 | 0 | 0 | 0.0544 | 22.4637 | 250 | 0.005024 | 524 | 145.4 | 2.11 | 14 | 0.2 | 1.477 | 3.5 | 595 |
| 1000 | 40 | 325 | 76.05 | 325 | 191.3 | 116194.8 | 0 | 0 | 0.0458 | 8.8881 | 0 | 0 | 0 | 149.2 | 2.11 | 14 | 0.2 | 1.477 | 3.1 | 794.2 |
| 1000 | 40 | 325 | 76.05 | 325 | 191.3 | 116194.8 | 0 | 0 | 0.0458 | 17.7997 | 0 | 0 | 0 | 138.7 | 2.11 | 14 | 0.2 | 1.477 | 3 | 982.6 |
| 1000 | 40 | 325 | 76.05 | 325 | 191.3 | 116194.8 | 0 | 0 | 0.0458 | 17.741 | 0 | 0 | 0 | 169.8 | 1.05 | 14 | 0.2 | 0.735 | 3 | 638.4 |
| 1000 | 40 | 325 | 76.05 | 325 | 191.3 | 116194.8 | 0 | 0 | 0.0458 | 17.7927 | 0 | 0 | 0 | 139.9 | 1.05 | 14 | 0.2 | 0.735 | 3 | 713.8 |
| 1000 | 40 | 325 | 76.05 | 325 | 191.3 | 116194.8 | 0 | 0 | 0.0458 | 17.754 | 250 | 0.005024 | 524 | 159.4 | 2.11 | 14 | 0.2 | 1.477 | 2.8 | 1083 |
| 300 | 70 | 220 | 50 | 290 | 62.5 | 42250 | 0 | 0 | 0.0659 | 27.072 | 0 | 0 | 0 | 169.2 | 2.5 | 13 | 0.16 | 2.03125 | 5.4 | 260 |
| 300 | 70 | 220 | 50 | 290 | 62.5 | 42250 | 0 | 0 | 0.0659 | 26.9658 | 0 | 0 | 0 | 189.9 | 2.5 | 13 | 0.16 | 2.03125 | 5.2 | 273 |
| 200 | 20 | 200 | 25 | 110 | 45 | 12550 | 0.216851 | 617 | 0 | 0 | 150 | 0.00942 | 625 | 150 | 3 | 13 | 0.2 | 1.95 | 2.8 | 115 |
| 200 | 20 | 200 | 25 | 110 | 45 | 12550 | 0.216851 | 617 | 0 | 0 | 0 | 0 | 0 | 150 | 3 | 13 | 0.2 | 1.95 | 2.8 | 92 |
| 330 | 30 | 200 | 60 | 200 | 60 | 30300 | 0.218056 | 617 | 0 | 0 | 0 | 0 | 0 | 213 | 0 | 0 | 0 | 0 | 4 | 41 |
| 330 | 30 | 200 | 60 | 200 | 60 | 30300 | 0.218056 | 617 | 0 | 0 | 105 | 0.007267 | 731 | 224 | 0 | 0 | 0 | 0 | 4 | 92 |
| 330 | 30 | 200 | 60 | 200 | 60 | 30300 | 0.218056 | 617 | 0 | 0 | 0 | 0 | 0 | 195 | 0 | 0 | 0 | 0 | 4 | 98 |
| 330 | 30 | 200 | 60 | 200 | 60 | 30300 | 0.218056 | 617 | 0 | 0 | 0 | 0 | 0 | 201 | 1 | 13 | 0.175 | 0.742857 | 4 | 109 |
| 330 | 30 | 200 | 60 | 200 | 60 | 30300 | 0.218056 | 617 | 0 | 0 | 0 | 0 | 0 | 208 | 1 | 13 | 0.175 | 0.742857 | 4 | 100 |
| 330 | 30 | 200 | 60 | 200 | 60 | 30300 | 0.218056 | 617 | 0 | 0 | 0 | 0 | 0 | 186 | 1 | 13 | 0.175 | 0.742857 | 4 | 109 |
| 330 | 30 | 200 | 60 | 200 | 60 | 30300 | 0.218056 | 617 | 0 | 0 | 105 | 0.007267 | 731 | 198 | 1 | 13 | 0.175 | 0.742857 | 4 | 147 |
| 330 | 30 | 200 | 60 | 200 | 60 | 30300 | 0.218056 | 617 | 0 | 0 | 105 | 0.007267 | 731 | 197 | 1 | 13 | 0.175 | 0.742857 | 4 | 170 |
| 330 | 30 | 200 | 60 | 200 | 60 | 30300 | 0.218056 | 617 | 0 | 0 | 0 | 0 | 0 | 213 | 0.5 | 13 | 0.175 | 0.371429 | 4 | 78 |
| 330 | 30 | 200 | 60 | 200 | 60 | 30300 | 0.218056 | 617 | 0 | 0 | 105 | 0.007267 | 731 | 211 | 0.5 | 13 | 0.175 | 0.371429 | 4 | 140 |
| 240 | 50 | 120 | 60 | 120 | 60 | 20400 | 0.031888 | 500 | 0 | 0 | 100 | 0.0157 | 500 | 167.5 | 0 | 0 | 0 | 0 | 1 | 106.08 |
| 240 | 50 | 120 | 60 | 120 | 60 | 20400 | 0.031888 | 500 | 0 | 0 | 0 | 0 | 0 | 181.2 | 2 | 13 | 0.2 | 1.3 | 1 | 216.6667 |
| 240 | 50 | 120 | 65 | 120 | 65 | 21100 | 0.027598 | 512 | 0 | 0 | 0 | 0 | 0 | 148 | 2.55 | 25 | 0.4 | 1.234018 | 1.25 | 197.8646 |
| 240 | 50 | 120 | 65 | 120 | 65 | 21100 | 0.027598 | 512 | 0 | 0 | 0 | 0 | 0 | 150 | 2 | 25 | 0.4 | 0.967857 | 1.25 | 187.4507 |
| 240 | 50 | 120 | 65 | 120 | 65 | 21100 | 0.027598 | 512 | 0 | 0 | 0 | 0 | 0 | 136 | 1.5 | 25 | 0.4 | 0.725893 | 1.25 | 160.2143 |
| 240 | 50 | 120 | 65 | 120 | 65 | 21100 | 0.027598 | 512 | 0 | 0 | 0 | 0 | 0 | 154 | 2.55 | 25 | 0.4 | 1.59375 | 1.25 | 187.4507 |
| 240 | 50 | 120 | 65 | 120 | 65 | 21100 | 0.027598 | 512 | 0 | 0 | 0 | 0 | 0 | 155 | 2 | 25 | 0.4 | 1.25 | 1.25 | 174.6336 |
| 240 | 50 | 120 | 65 | 120 | 65 | 21100 | 0.027598 | 512 | 0 | 0 | 0 | 0 | 0 | 138 | 1.5 | 25 | 0.4 | 0.9375 | 1.25 | 128.1714 |
| 240 | 50 | 120 | 65 | 120 | 65 | 21100 | 0.027598 | 512 | 0 | 0 | 0 | 0 | 0 | 148 | 2.55 | 25 | 0.4 | 1.234018 | 1.25 | 190 |
| 240 | 50 | 120 | 65 | 120 | 65 | 21100 | 0.027598 | 512 | 0 | 0 | 0 | 0 | 0 | 136 | 1.5 | 25 | 0.4 | 0.725893 | 1.25 | 165 |
| 240 | 50 | 120 | 65 | 120 | 65 | 21100 | 0.027598 | 512 | 0 | 0 | 0 | 0 | 0 | 154 | 2.55 | 25 | 0.4 | 1.59375 | 1.25 | 190 |
| 240 | 50 | 120 | 65 | 120 | 65 | 21100 | 0.027598 | 512 | 0 | 0 | 0 | 0 | 0 | 155 | 2 | 25 | 0.4 | 1.25 | 1.25 | 177 |
| 240 | 50 | 120 | 65 | 120 | 65 | 21100 | 0.027598 | 512 | 0 | 0 | 0 | 0 | 0 | 138 | 1.5 | 25 | 0.4 | 0.9375 | 1.25 | 140 |
| 300 | 50 | 200 | 40 | 0 | 0 | 21000 | 0.032154 | 400 | 0.0224 | 4.066667 | 0 | 0 | 0 | 171 | 2 | 13 | 0.2 | 1.3 | 1 | 450 |
| 300 | 50 | 200 | 40 | 0 | 0 | 21000 | 0.032154 | 400 | 0.0224 | 4.013333 | 0 | 0 | 0 | 171 | 2 | 13 | 0.2 | 1.3 | 1.5 | 317 |
| 300 | 50 | 200 | 40 | 0 | 0 | 21000 | 0.032154 | 400 | 0.0224 | 6.706667 | 0 | 0 | 0 | 171 | 2 | 13 | 0.2 | 1.3 | 1 | 486 |
| 300 | 50 | 200 | 40 | 0 | 0 | 21000 | 0.032154 | 400 | 0.0224 | 7.08 | 0 | 0 | 0 | 171 | 2 | 13 | 0.2 | 1.3 | 1.5 | 346 |
| 300 | 50 | 200 | 40 | 0 | 0 | 21000 | 0.018086 | 400 | 0.0224 | 6.44 | 0 | 0 | 0 | 171 | 2 | 13 | 0.2 | 1.3 | 1 | 451 |
| 300 | 50 | 200 | 40 | 0 | 0 | 21000 | 0.05024 | 400 | 0.0224 | 7.36 | 0 | 0 | 0 | 179 | 2 | 13 | 0.2 | 1.3 | 1 | 485 |
| 300 | 50 | 200 | 40 | 0 | 0 | 21000 | 0.032154 | 400 | 0.0224 | 6.44 | 0 | 0 | 0 | 163 | 1 | 13 | 0.2 | 0.65 | 1 | 427 |
| 300 | 50 | 200 | 40 | 0 | 0 | 21000 | 0.032154 | 400 | 0.0224 | 6.653333 | 0 | 0 | 0 | 171 | 1.5 | 13 | 0.2 | 0.975 | 1 | 459 |
| 300 | 50 | 200 | 40 | 0 | 0 | 21000 | 0.032154 | 400 | 0.0224 | 6.706667 | 125 | 0.016077 | 400 | 171 | 2 | 13 | 0.2 | 1.3 | 1 | 590 |
| 400 | 50 | 360 | 60 | 0 | 0 | 38600 | 0.011164 | 475 | 0.031111 | 16.6 | 0 | 0 | 0 | 175 | 2.5 | 13 | 0.2 | 1.625 | 1.44 | 510.5 |
| 400 | 50 | 360 | 60 | 0 | 0 | 38600 | 0.011164 | 475 | 0.031111 | 17.5 | 0 | 0 | 0 | 175 | 2.5 | 13 | 0.2 | 1.625 | 2.56 | 263.35 |
| 400 | 50 | 360 | 60 | 0 | 0 | 38600 | 0.011164 | 475 | 0.031111 | 17 | 200 | 0.005652 | 456 | 175 | 2.5 | 13 | 0.2 | 1.625 | 2.56 | 374.5 |
| 400 | 50 | 360 | 60 | 0 | 0 | 38600 | 0.054514 | 475 | 0.031111 | 17.5 | 0 | 0 | 0 | 175 | 2.5 | 13 | 0.2 | 1.625 | 3.67 | 229.2 |
| 350 | 80 | 250 | 60 | 0 | 0 | 38200 | 0.0297 | 668 | 0 | 0 | 0 | 0 | 0 | 108 | 0 | 0 | 0 | 0 | 2 | 174.3 |
| 350 | 80 | 250 | 60 | 0 | 0 | 38200 | 0.0297 | 668 | 0 | 0 | 210 | 0.005981 | 472 | 108 | 0 | 0 | 0 | 0 | 2 | 373.7 |
| 350 | 80 | 250 | 60 | 0 | 0 | 38200 | 0.0297 | 668 | 0 | 0 | 150 | 0.008373 | 472 | 108 | 0 | 0 | 0 | 0 | 2 | 411.7 |
| 350 | 80 | 250 | 60 | 0 | 0 | 38200 | 0.0297 | 668 | 0 | 0 | 120 | 0.010467 | 472 | 108 | 0 | 0 | 0 | 0 | 2 | 476.3 |
| 350 | 80 | 250 | 60 | 0 | 0 | 38200 | 0.0297 | 668 | 0 | 0 | 0 | 0 | 0 | 130 | 1 | 13 | 0.2 | 0.65 | 2 | 403.3 |
| 350 | 80 | 250 | 60 | 0 | 0 | 38200 | 0.0297 | 668 | 0 | 0 | 0 | 0 | 0 | 134 | 2 | 13 | 0.2 | 1.3 | 2 | 580 |
| 350 | 80 | 250 | 60 | 0 | 0 | 38200 | 0.0297 | 668 | 0 | 0 | 0 | 0 | 0 | 138 | 3 | 13 | 0.2 | 1.95 | 2 | 729.7 |
| 350 | 80 | 250 | 60 | 0 | 0 | 38200 | 0.0297 | 668 | 0 | 0 | 0 | 0 | 0 | 134 | 2 | 13 | 0.2 | 1.3 | 1 | 1403 |
| 350 | 80 | 250 | 60 | 0 | 0 | 38200 | 0.0297 | 668 | 0 | 0 | 0 | 0 | 0 | 134 | 2 | 13 | 0.2 | 1.3 | 3 | 363.3 |
